# Design, Synthesis, and Self-Assembly of Amphiphilic 1,4-Dihydropyridines Containing Branched Ester Moieties

**DOI:** 10.3390/molecules31071161

**Published:** 2026-03-31

**Authors:** Davis Lacis, Martins Rucins, Nadiia Pikun, Ruslans Muhamadejevs, Karlis Pajuste, Mara Plotniece, Juris Jansons, Anna Zajakina, Arkadij Sobolev, Aiva Plotniece

**Affiliations:** 1 Latvian Institute of Organic Synthesis, Aizkraukles 21, LV-1006 Riga, Latvia; davis.lacis@osi.lv (D.L.); rucins@osi.lv (M.R.); nadiia@osi.lv (N.P.); muhamadejev@osi.lv (R.M.); kpajuste@osi.lv (K.P.); arkady@osi.lv (A.S.); 2 Faculty of Natural Sciences and Technology, Riga Technical University, P. Valdena 3, LV-1048 Riga, Latvia; mara.plotniece@rtu.lv; 3 Department of Applied Pharmacy, Faculty of Pharmacy, Riga Stradiņš University, Konsula 21, LV-1007 Riga, Latvia; 4 Latvian Biomedical Research and Study Centre, Ratsupites 1 k-1, LV-1067 Riga, Latvia; jansons@biomed.lu.lv (J.J.); anna@biomed.lu.lv (A.Z.)

**Keywords:** 1,4-dihydropyridine amphiphiles, branched hydrophobic moieties, self-assembling properties, nanoparticles, Lipophilicity (AlogP)

## Abstract

Amphiphilic cationic lipids based on the 1,4-dihydropyridine (1,4-DHP) scaffold represent a versatile platform for the development of self-assembling delivery systems. In this work, a series of ten new amphiphilic 1,4-DHP derivatives bearing branched ester substituents at the 3,5-positions and quaternized cationic groups at the 2,6-positions were designed and synthesized. The effect of branched ester chain length and branching on nanoparticle formation was investigated. The self-assembling properties of the synthesized amphiphiles were evaluated by dynamic light scattering using an ethanol injection method. All compounds formed positively charged nanoparticles with hydrodynamic diameters ranging from 52 to 439 nm and polydispersity index from 0.194 to 0.452. Amphiphiles **14b**–**17b** with 2-hexyldecyl substituents formed smaller particles, with an average diameter below 100 nm. Several derivatives exhibited good stability over a 14-day storage period at room temperature. To clarify structure–property relationships, lipophilicity (AlogP), polar surface area (PSA), and pK_a_ values were calculated using Schrödinger computational tools. The compounds displayed high lipophilicity AlogP 8.98–19.32, while PSA values remained within a narrow range. The calculated pK_a_ values ranged from 7.20 to 10.99. The results demonstrate that both the length and architecture of branched ester chains significantly influence nanoparticle size, homogeneity, and stability, highlighting branched-chain 1,4-DHP amphiphiles as promising synthetic lipid candidates for further development of delivery systems after evaluation of biological properties.

## 1. Introduction

Liposomes and lipid nanoparticles (LNPs) have become central components of contemporary drug and gene delivery technologies, particularly following their successful application in mRNA vaccines [1,2,3,4]. LNPs are capable of spontaneous self-assembly and efficient encapsulation of therapeutic cargos while simultaneously protecting nucleic acids from enzymatic degradation. Additionally, LNPs form a versatile nanocarrier platform of growing interest in oncology for theranostic applications [5,6,7]. Liposomal antimicrobials are employed in the treatment of bacterial and fungal infections [8,9,10].

The physicochemical properties of lipid nanoparticles are governed largely by the architecture of their hydrophobic domains. Alkyl chain length, degree of saturation, and molecular branching influence lipid packing, membrane fluidity, and nanoparticle morphology [11,12,13]. In particular, branching within the hydrophobic region can increase the effective molecular volume and disrupt ordered lipid packing, effects that have been associated with enhanced endosomal escape and improved transfection efficiency in RNA delivery systems [14,15]. Additionally, such branching has been correlated with heightened transfection efficiency [16,17]. Despite these advances, systematic studies addressing the role of branched hydrophobic substituents in non-classical lipid scaffolds remain comparatively limited.

Most investigations of branched hydrophobic tails have been conducted within conventional phospholipid or ionizable lipid architectures derived from glycerol or tertiary amine frameworks, where branching has been optimized primarily to enhance membrane fusion, endosomal escape, and transfection efficiency [18,19]. As a result, the effects of hydrophobic branching have been evaluated largely within structurally similar backbones, and comparatively little attention has been devoted to understanding how such architectural modifications behave in alternative, heterocycle-based amphiphilic systems. Although these studies have established general principles linking branching to altered packing and fusogenicity, it remains unclear whether these relationships are transferable to non-classical lipid scaffolds with distinct molecular geometry and rigidity. In this context, the 1,4-dihydropyridine (1,4-DHP) core represents a structurally defined heterocyclic platform in which hydrophobic ester substituents at the 3,5-positions and cationic headgroups at the 2,6-positions are symmetrically arranged around a rigid framework [20,21]. This architecture enables systematic modulation of hydrophobic volume and headgroup characteristics while preserving the overall molecular geometry, thereby providing a suitable model for probing structure–assembly relationships beyond conventional glycerol-based lipid systems.

**Figure 1:**
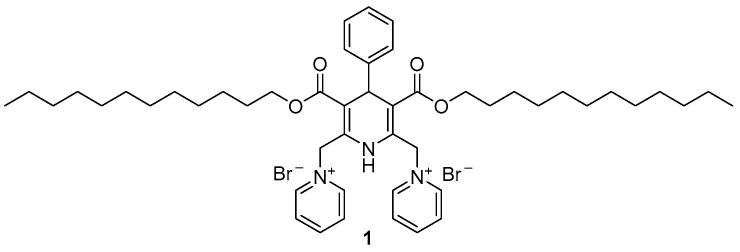
Structure of cationic 1,4-dihydropyridine amphiphile **1**.

Our previous studies demonstrated that amphiphilic 1,4-dihydropyridine (1,4-DHP) derivatives bearing pyridinium headgroups exhibit self-assembling behavior in aqueous media and form liposome-like nanoparticles. Several representatives, including compound **1** (Figure 1), showed higher DNA delivery activity than the commercial transfection agents DOTAP and PEI (25 kDa), indicating the potential of this scaffold for transmembrane delivery applications [20,21]. The 1,4-DHP fragment serves as a functional linker and is also a key structural motif found in numerous biologically active compounds [22,23,24]. Pyridinium-containing 2,3,4,5-tetrahydropyridine derivatives have also demonstrated the ability to form liposomes [25]. Amphiphilic derivatives have shown antiproliferative activity [26].

Building on these findings, we aimed to study the influence of branched ester substituents at the 3,5-positions of the 1,4-DHP core on self-assembly behavior and nanoparticle characteristics. Herein, a series of new amphiphilic 1,4-DHP derivatives incorporating branched alkyl ester moieties, such as 2-hexyldecyl and 2-ethylhexyl, were designed and synthesized. Their self-assembling properties were systematically evaluated by dynamic light scattering, with particular emphasis on nanoparticle size, size distribution, surface charge, and stability. The results provide new insights into structure–assembly relationships within branched-chain 1,4-DHP amphiphiles and support their further development as synthetic lipid components for delivery systems.

## 2. Results and Discussion

### 2.1. Synthesis of 1,4-DHPs

**Scheme 1:**
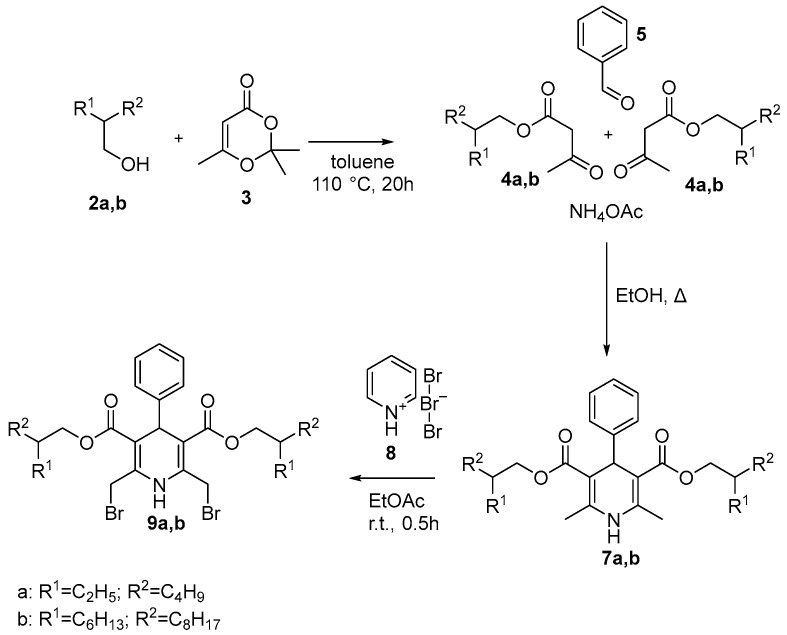
Synthesis of 2,6-bis(bromomethyl)-1,4-DHPs **9a**,**b**.

To study the effect of branched alkyl chain length on the self-assembly behavior of 1,4-DHP amphiphiles, a series of compounds bearing branched ester substituents at the 3,5-positions was synthesized. The corresponding β-keto esters **4a**,**b** were obtained by reaction of 2,2,6-trimethyl-4*H*-1,3-dioxin-4-one with the appropriate alcohols in toluene, affording the desired products in 71% and 78% yield, respectively (Scheme 1). Subsequent Hantzsch cyclization of the β-keto esters **4a**,**b** with benzaldehyde **5** and ammonium acetate afforded the intermediate 1,4-DHP derivatives **7a**,**b** in 60% and 53% yield, respectively (Scheme 1).

The selection of branched ester moieties was guided by both structural considerations and relevance to lipid nanoparticle design. In particular, the 2-hexyldecyl substituent was chosen based on its structural similarity to branched lipids employed in clinically relevant mRNA delivery systems [27], while the shorter 2-ethylhexyl group enabled assessment of hydrophobic chain length effects. Racemic alcohols were used because the main focus was the comparison of amphiphiles with linear and branched alkyl chains, particularly the length of the branched groups, rather than the effects of chirality. These two alcohols were selected to introduce branched hydrophobic chains with different lengths and bulkiness. The 2-ethylhexyl substituent represents a moderately branched chain, whereas the 2-hexyldecyl substituent provides a larger hydrophobic volume. This design enables evaluation of the effect of hydrophobic chain length and branching on amphiphile self-assembly and physicochemical properties and allows comparison with previously reported amphiphiles bearing linear ester moieties. The structure of compound **4a** was confirmed by the ^1^H-NMR spectra, which agrees with what has been previously reported in the literature [28]. Structures of compounds **4b** and 1,4-DHPs **7a**,**b** were confirmed based on ^1^H-NMR, ^13^C-NMR spectra, and HRMS data (Figures S1–S8, Supplementary Materials).

Bromination of the 2,6-methyl groups of 1,4-DHPs **7a**,**b** using pyridinium bromide–perbromide afforded the corresponding 2,6-bis(bromomethyl) derivatives **9a**,**b** in high yields within short reaction times. Compared to conventional N-bromosuccinimide-based protocols [21], this method [29] provided efficient and reproducible access to the desired intermediates under mild conditions. The desired 2,6-bis(bromomethyl)-1,4-DHPs **9a**,**b** were successfully obtained with yields of 83% and 84%, respectively (Scheme 1).

The structures of formed 2,6-bis(bromomethyl)-1,4-DHP **9a**,**b** were confirmed by ^1^H-NMR, ^13^C-NMR spectroscopy, and HRMS analysis. Notably, the characteristic proton signal in ^1^H-NMR spectra corresponding to the methyl groups of 1,4-DHP **7a**,**b**, typically observed as a singlet at 2.34–2.35 ppm (Figures S3 and S6, Supplementary Materials), disappeared after bromination. Instead, new signals, corresponding to an AB system characteristic for the 2,6-bis(bromomethyl)-1,4-DHP group, were observed. A similar transformation has been reported in our previous studies [21,30], providing further confirmation of the successful formation of 2,6-bis(bromomethyl)-1,4-DHP compounds. For 2,6-bis(bromomethyl)-1,4-DHPs **9b** with 2-hexyldecyl ester moieties, AB system protons appeared as typical AB system doublets at 4.94 and 4.64 ppm with a coupling constant of 11.5 Hz (Figure S12, Supplementary Materials). For 2,6-bis(bromomethyl)-1,4-DHPs **9a** with a shorter 2-ethylhexyl ester group, the bromomethylene group proton signals of the AB system exhibited additional splitting. The corresponding signals were observed as a doublet of multiplets at 4.96–4.91 ppm and a doublet of triplets at 4.63 ppm (Figure S9, Supplementary Materials). This indicates further interactions, presumably with the 3,5-position ester −OCH_2_ group, as reported in similar structures [30,31]. For compounds **9a**,**b,** ^1^H-NMR, ^13^C-NMR spectra, and HRMS data are provided in Supplementary Materials (Figures S9–S11, S13 and S14, Supplementary Materials).

### 2.2. Synthesis of Cationic 1,4-DHP Amphiphiles

**Scheme 2:**
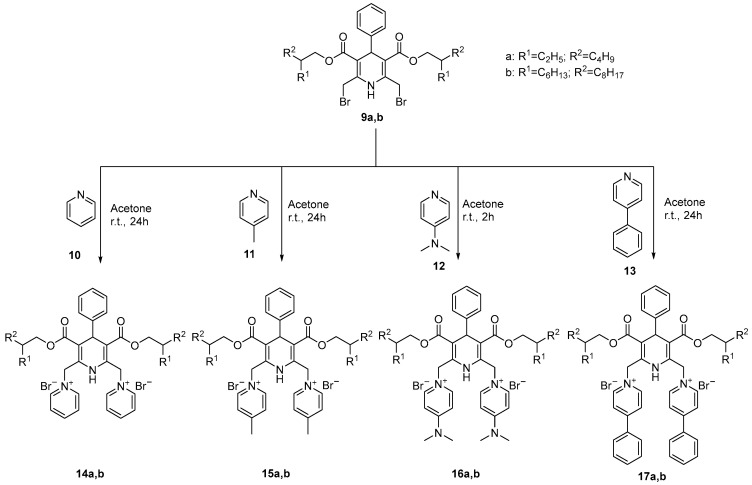
Synthesis of 1,4-DHP amphiphiles **14a**,**b**, **15a**,**b**, **16a**,**b**, and **17a**,**b**.

In our previous studies, we investigated the self-assembling properties of diverse cationic 3,5-bis(dodecyloxycarbonyl)-1,4-DHP derivatives, which characterized their formed liposomes and demonstrated their potential as gene delivery agents [21,32]. Building on these findings, a new series of 1,4-DHP amphiphiles bearing branched ester chains at positions 3 and 5 and unsubstituted or substituted pyridinium moieties at 2,6-positions was synthesized. Synthetic approach and the structures of 1,4-DHP amphiphiles **14a**,**b**, **15a**,**b**, **16a**,**b**, and **17a,b** were described in Scheme 2 and Table 1. This expansion of the compound library was undertaken to enable the assessment of structure–activity relationships.

The target 1,4-DHP amphiphiles **14a**,**b**, **15a**,**b**, **16a**,**b**, and **17a**,**b** were synthesized via bromine nucleophilic substitution of bromomethyl groups of 1,4-DHP **9a**,**b** with selected pyridine derivatives (Scheme 2). Pyridine (**10**), 4-methylpyridine (**11**), and 4-dimethylaminopyridine (**12**) were selected based on the previously reported results, where amphiphiles with the mentioned cationic moieties demonstrated the highest transfection efficiency [21]. 4-phenylpyridine (**13**) was selected to increase the lipophilicity of the cationic moiety.

The bromine nucleophilic substitution reactions of 2,6-bis(bromomethyl)-1,4-DHP **9a**,**b** with a range of pyridine derivatives were performed in acetone, resulting in the formation of the target 1,4-DHP amphiphiles **14a**,**b**, **15a**,**b**, **16a**,**b**, and **17a**,**b** (Scheme 2, Table 1, entries 1–8). The reactions proceeded smoothly in acetone at room temperature (r.t.), with isolated yields ranging from moderate to good. Notably, substitution with 4-dimethylaminopyridine (**12**) proceeded more rapidly, and the corresponding 1,4-DHP amphiphiles **16a**,**b** were obtained after 2 h (Table 1, entries 5 and 6), while other reactions were completed in 24 h. The increase in the reaction rate is likely associated with the strong electron-donating dimethylamino group, which increases the nucleophilicity of the pyridine nitrogen and facilitates quaternization. The obtained yields of 1,4-DHP amphiphiles ranged from 22% to 72%, depending on the nucleophilicity of pyridines **10–13** and ester substituents at the 3,5-positions of 1,4-DHP **9a**,**b**. The lower yield observed for compound **14a**, only 22%, is associated with good solubility of the compound in purification, rather than incomplete reaction conversion. Details regarding the pyridine derivatives, reaction times, and product yields are provided in Table 1.

The structures of compounds **14a**,**b**, **15a**,**b**, **16a**,**b**, and **17a**,**b** were confirmed by ^1^H-NMR, ^13^C-NMR spectra, and HRMS data (Figures S15–S38, Supplementary Materials). The ^1^H-NMR spectra revealed a notable downfield shift of the AB system at the 2,6-bis(methylene) positions due to quaternization to higher chemical shifts. In CDCl_3_, the signals shifted from 4.63 and 4.93 ppm to 6.36 and 5.96 ppm, whereas in DMSO-*d*_6_, they appeared in the range of 5.59–6.17 and 5.23–5.63 ppm, depending on the pyridinium substituent. These downfield shifts are consistent with the formation of quaternized nitrogen-containing headgroups in compounds **14a**,**b**, **15a**,**b**, **16a**,**b**, and **17a**,**b**.

According to high-performance liquid chromatography (HPLC) data, the purities of the studied compounds **14a**,**b**, **15a**,**b**, **16a**,**b**, and **17a**,**b** were at least 97%.

### 2.3. Evaluation of Self-Assembling Properties, Lipophilicity (AlogP), Polar Surface Area (PSA), and Acid Ionization Constant (pKa) of Amphiphiles

**Figure 2:**
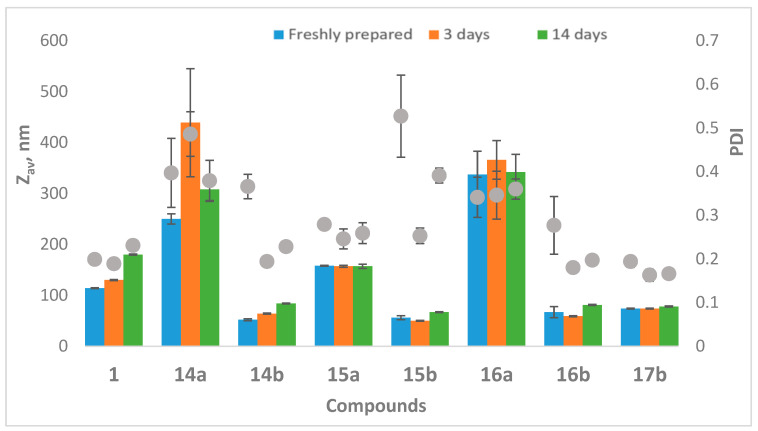
Values of Z-average diameter (Z_av_) (colored bars) and polydispersity index (PDI) (grey dots) of nanoparticles formed by branched ester moieties containing 1,4-DHP amphiphiles **14a**,**b**, **15a**,**b**, **16a**,**b** and **17b** in water; data obtained by DLS measurements for freshly prepared samples and after storage for 3 and 14 days at r.t., concentration of samples 0.5 mM. Error bars smaller than data point size are not visible.

Self-assembly is a characteristic feature of lipid-like compounds, including cationic 1,4-DHP amphiphiles. Previously, particles formed by structurally related compounds, including comp. **1,** were confirmed using microscopy techniques [21,33]. Therefore, we assume that similar particles are also formed by the present compounds and analyze the structure–property relationships based on dynamic light scattering data. The average hydrodynamic diameter (Z_av_), polydispersity index (PDI), zeta potential (Z_pot_), and stability of nanoparticles formed by branched alkyl ester chain containing 1,4-DHP amphiphiles **14a**,**b**, **15a**,**b**, **16a**,**b**, and **17a,b** were determined by the dynamic light scattering (DLS) method. All DLS measurements were performed under standardized conditions in deionized water at 25 °C. The solutions were prepared using the same protocol to ensure reproducibility and give the possibility to compare a data with the results of previously studied structurally related amphiphiles. The obtained results were compared with those of compound **1**, which served as a reference compound due to its previously reported self-assembling properties. Nanoparticles were prepared via the rapid and adaptable ethanol injection method [34]. This method has also been applied in our group for various lipid-like compounds [25,32]. The final concentration of amphiphiles in all samples was 0.5 mM. DLS measurements were performed immediately after sample preparation, and nanoparticle stability was evaluated after storage at r.t. for 3 and 14 days. The results are presented in Figure 2 (data from Table S1 and Figures S39–S45 in Supplementary Materials).

The average diameter of nanoparticles formed by branched ester chains containing 1,4-DHP derivatives **14a**,**b**, **15a**,**b**, **16a**,**b**, and **17b** in aqueous medium ranged from 52 to 337 nm for the freshly prepared samples. In the case of sample **17a,** dispersion of the stock solution in water resulted in aggregation during preparation; therefore, this compound was excluded from further studies. The obtained results indicate that, when the cationic substituents were kept constant, increasing the length of the branched ester chains influenced the properties of nanoparticles formed by the 1,4-DHP amphiphiles. Thus, the average diameter of nanoparticles formed from amphiphiles with 2-hexyldecyl ester chains **14b**, **15b,** and **16b** (Table S1, Supplementary Materials) was three to five times smaller than that formed by the corresponding 2-ethylhexyl derivatives **14a**, **15a,** and **16a**. This suggests that longer hydrophobic chains promote more compact self-assembly, leading to smaller nanoparticles. Similar observations have been reported for sphingomyelins, where variation of the amide-linked acyl chain length (C16–C24) influenced lipid packing and membrane properties [35]. The reference 1,4-DHP amphiphile **1** formed nanoparticles with an average diameter of 114 nm for the freshly prepared sample. Upon storage, particle diameters increased to 130 nm after 3 days and to 180 nm after 14 days, while PDI values remained in the range of 0.199 to 0.231, indicating relatively good stability. A comparable trend was observed in our earlier studies [33].

The average diameters of the nanoparticles formed by the studied 1,4-DHP amphiphiles after 3 days of storage ranged from 50 to 439 nm, whereas after 14 days of storage, they ranged from 67 to 342 nm. It was observed that 2-hexyldecyl ester chains containing 1,4-DHP amphiphiles **14b**, **15b**, **16b**, and 1**7b** formed homogeneous particles with average diameters in the range from 50 to 85 nm and PDI values below 0.35 (Table S1 and Figures S40, S42, S44 and S45, Supplementary Materials). These samples remained stable for 14 days of storage. Nanoparticles formed by 2-ethylhexyl ester chains containing 1,4-DHP amphiphile **16a** also showed good stability during storage, maintaining initial parameters—average diameters around 350 nm and PDI values around 0.350. In contrast, nanoparticles formed by amphiphile 14a exhibited significant variation in the average nanoparticle diameter over the 14-day storage period, indicating a lack of stability. Thus, the average diameter increased from 250 nm in freshly prepared samples to 439 nm after 3 days of storage, followed by a decrease to 308 nm after 14 days of storage. Larger hydrodynamic diameters around 400 nm observed for compounds **14a** and **16a** may indicate the formation of vesicle-like aggregates, whereas smaller particles below 100 nm observed for amphiphiles **14b**–**17b** with 2-hexyldecyl substituents may correspond to smaller particles. Previous studies also demonstrated the effect of structural parameters of 1,4-DHP amphiphiles on particle size [21,32].

Morphology of the samples of particles formed by selected 1,4-DHP amphiphiles was studied using the transmission electron microscopy (TEM) technique. Representative TEM images of samples are given in Figure S53 (Supplementary Materials). According to the obtained data, all samples contained almost spherical in shape particles. The obtained TEM data is also in agreement with the DLS measurement results, confirming smaller particles below 100 nm for amphiphiles 14b and 15b.

PDI is a measure of the distribution of particle sizes within a sample. In drug delivery applications using lipid-based carriers, such as liposome formulations, a PDI of 0.3 and below is acceptable and indicates a homogeneous population of particles [36]. From the obtained data, the PDI values for freshly prepared samples are below 0.3 in most of the cases, except for samples of compounds **14a**,**b**, **15b**, and **16a**. After 3 days of storage, a decrease in PDI values was observed for all samples with 2-hexyldecyl ester chains, compounds **14b**, **15b**, and **16b**. After 14 days of storage, these samples exhibited only minor changes in PDI values. PDI values of samples prepared from 1,4-DHP amphiphiles **14b**, **15a**, **16b**, and **17b** remained constant over the 14-day storage period, with only minimal changes observed within the margin of errors, indicating good stability.

Comparing the data for amphiphiles containing branched ester chains with the linear analog, the most similar results were observed for compounds **15a** and **1**, with average diameter sizes of 155–157 nm and 115–180 nm and PDI values of approximately 0.25 and 0.23, respectively. Zeta potential serves as a critical parameter in characterizing liposomes, providing insights into the surface charge of the particles and their propensity for aggregation or dispersion within a formulation. Zeta potential values for amphiphilic 1,4-DHP containing branched ester groups are presented in Table 2. For compounds **14a**,**b**, **15a**,**b**, **16a**,**b**, and **17a**,**b**, AlogP and polar surface area (PSA) values were calculated using the Schrödinger Maestro interactive properties module (Schrödinger, LLC, New York, NY, USA) [37]. The pKa values were calculated with Jaguar pKa (Schrödinger, LLC) [38]. For the calculation of the AlogP, PSA, and pKa values, all compounds were taken as cations (all anions were removed) to allow the calculation to be done for a single molecule. The calculated AlogP, PSA, and pKa values are presented in Table 2.

Typically, liposomes exhibiting zeta potentials greater than +30 mV or less than −30 mV are regarded as stable, as these values reflect sufficient electrostatic repulsion to prevent aggregation and enhance colloidal stability [39,40]. In our study, all compound nanoparticles, **14a**,**b**, **15a**,**b**, **16a**,**b**, and **17b**, exhibited positive surface charges, with zeta potential **b** values ranging from 16.1 to 47.7 mV (Table 2 and Figures S46–S52, Supplementary Materials), while the zeta potential value for nanoparticles of comp. **1** was higher, around 75 mV. Notably, the zeta potentials of most compounds exceeded the 30 mV threshold, suggesting good stability against aggregation. However, amphiphiles **14a** and **16a** exhibited zeta potentials below 30 mV, indicating lower surface charge and potentially reduced colloidal stability compared to the other compounds. The comparatively low zeta potential values may be associated with the larger average nanoparticle diameter for these compounds, leading to the initial formation of larger particles.

Lipophilicity reflects a molecule’s affinity for lipophilic environments and is expressed as logP [41,42]. Polar surface area (PSA), the sum of the surfaces of polar atoms, is used to assess molecular polarity and predict drug transport properties [43]. Calculated topological PSA also indicates blood–brain barrier permeability and helps guide further development of pharmaceutically active compounds [44]. In our studies, no significant differences in PSA values were observed among the compounds. The PSA value of comp. **1** is around 70.64 Å^2^, whereas for new 1,4-DHP amphiphiles containing branched ester moieties, PSA values are 72.39 Å^2^ or 78.87 Å^2^. It should be noted that the main differences are related not to the structure of the branched alkyl chains but to the structure of the polar moiety. For example, the dimethylamino pyridinium-containing amphiphiles **16a**,**b** exhibit slightly higher PSA values. AlogP values that surpass 5, according to Lipinski’s Rule of Five, characterize compounds as lipophilic [45]. According to the obtained data for the tested 1,4-DHP amphiphiles, AlogP values are in the range from 8.98 to 19.32. A comparison of 2-ethylhexyl-substituted 1,4-DHP amphiphiles **14a**–**17a** with their 2-hexyldecyl-substituted counterparts **14b**–**17b** reveals an approximately 7-unit increase in AlogP values for compounds containing identical cationic moieties due to the contribution of the longer branched alkyl chains.

In drug delivery systems, the acid dissociation constant (pKa) is a critical parameter because it determines the ionization state of a compound at physiological and intracellular pH, directly influencing solubility, membrane interaction, stability, and cargo release [46,47]. In general, the human physiological pH for most tissues is around 7~7.4, with some exceptions [48]. According to the obtained data, pKa values of the branched ester group containing 1,4-DHP amphiphiles are in the range from 7.20 to 10.99, which is in good agreement with the experimentally determined pKa value ~7.4 for compound **1**, possessing linear dodecyl ester moieties at 3,5-positions and pyridinium moieties at 2,6-positions of 1,4-DHP. Introducing cationic moieties at the 2,6-positions of the 1,4-DHP ring significantly increases the acidity of the NH proton, possessing pKa ≈ 7.2–11, whereas 1,4-DHPs with 2,6-dimethyl substituents exhibit much higher pKa values in the range of 19–20 [49].

## 3. Materials and Methods

### 3.1. General

All reagents were purchased from Thermo Fisher Scientific Chemicals (Waltham, MA, USA), Merck KGaA (Darmstadt, Germany), and BLDpharm (Shanghai, China) and were used without further purification. TLC was performed on silica gel 60 F_254_ aluminum sheets 20 cm × 20 cm (Merck KGaA, Darmstadt, Germany) and visualized by UV (254 nm or 365 nm) fluorescence. Silica gel column chromatography was performed using Merck silica gel (35–70 µm) with an ARMEN instrument SPOT Liquid Chromatography FLASH system (Armen Instrument, Saint-Avé, France). ^1^H-NMR spectra were recorded with a Bruker Avance Neo (600 MHz or 400 MHz) or Bruker Fourier (300 MHz) spectrometer, and ^13^C-NMR spectra were recorded with a Bruker Avance Neo (151 MHz or 101 MHz) spectrometer (Bruker BioSpin GmbH, Rheinstetten, Germany). ^1^H-NMR spectra were calibrated to the residual solvent peaks of undeuterated chloroform in CDCl_3_ or undeuterated dimethyl sulfoxide in DMSO-d_6_ (CDCl_3_: δ 7.26 ppm or DMSO-d_6_: δ 2.50 ppm). ^13^C-NMR spectra were calibrated to the ^13^C solvent signal (CDCl_3_: δ 77.16 ppm or DMSO-d_6_: δ 39.52 ppm). Chemical shifts (δ) are given in parts per million (ppm), and coupling constants are given in hertz (Hz). Abbreviations: s—singlet, br s—broad singlet, d—doublet, t—triplet, m—multiplet, dt—doublet of triplets, qd—quartet of doublets, and ddm—doublet of doublet of multiplets. High-resolution mass spectra (HRMS) were determined on an Acquity UPLC H-Class system (Waters, Milford, MA, USA) connected to a Waters Synapt GII Q-ToF operating in the ESI positive ion mode on a Waters Acquity UPLC^®^ BEH C18 column (1.7 µm, 2.1 mm × 50 mm, using gradient elution with acetonitrile (0.1% formic acid) in water (0.1% formic acid). Low-resolution mass spectra (MS) were determined on an Acquity UPLC system (Waters, Milford, MA, USA) connected to a Waters SQ Detector-2 operating in the electrospray ionization (ESI) positive or negative ion mode on a Waters Acquity UPLC^®^ BEH C18 column (1.7 µm, 2.1 × 50 mm, using gradient elution with acetonitrile (0.01% formic acid) in water (0.01% formic acid). Melting points (m.p.) were determined on an OptiMelt (Stanford Research Systems, Sunnyvale, CA, USA) and are uncorrected.

### 3.2. Self-Assembling Properties of Compounds by Dynamic Light Scattering Measurements

The self-assembling properties of the synthesized compounds were evaluated according to a previously reported procedure with minor modifications [21,50]. Briefly, stock solutions of compounds **1**, **14a**,**b**, **15a**,**b**, **16a**,**b**, and **17b** in EtOH were prepared at a concentration of 5 mM. Aliquots of the stock solutions of the compounds (0.2 mL, 5 mM in EtOH (96%)) were rapidly injected into deionized water (1.8 mL) under vigorous vortex mixing (IKA Vortex 2, IKA, Staufen, Germany), yielding samples with a final compound concentration of 0.5 mM. After which, samples were sonicated for 60 min using a bath-type sonicator (EMAG Emmi-H22 (EMAG, Mörfelden-Walldorf, Germany)). Before DLS measurements, all samples were allowed to cool to r.t.

The DLS measurements of the prepared aqueous solutions of the nanoparticles were carried out on a Zetasizer Nano ZSP (Malvern Panalytical Ltd., Malvern, UK) instrument with Malvern Instruments Ltd. Software 8.01.4906, using the following specifications: medium: water; refractive index: 1.33; viscosity: 0.8872 cP; temperature: 25 °C; dielectric constant: 78.5; nanoparticles: liposomes; refractive index of materials: 1.60; detection angle: 173°; and wavelength: 633 nm. The data were analyzed using multimodal number distribution software provided with the instrument. To ensure reproducibility, the measurements were repeated five times.

### 3.3. Determination of AlogP, PSA, and pKa

For compounds **14a**,**b**–**17a**,**b**, AlogP and polar surface area (PSA) values were calculated using Schrödinger Maestro interactive properties module (Schrödinger, LLC) [37]. For the calculation of the AlogP values, all amphiphilic compounds were taken as cations (all anions were removed), and the calculation was done for a single molecule. pKa values were calculated with Jaguar pKa (Schrödinger, LLC) [38].

### 3.4. Transmission Electron Microscopy (TEM)

The morphology of nanoparticles formed by selected 1,4-DHP amphiphiles **14a**,**b** and **15a**,**b** was studied by TEM. Samples of 1,4-DHP were prepared according to the procedure described in Section 3.2. The EM visualization was performed with uranyl acetate negative staining. First, 5 µL of the sample was absorbed on carbon formvar-coated 300 Mesh Copper grids (Agar Scientific, Stansted, UK; 2 grids per sample were prepared), incubated for 3 min, and negatively stained with 0.5% uranyl acetate aqueous solution. The grids were analyzed with a JEM-1230 electron microscope (JEOL, Tokyo, Japan) at an accelerating voltage of 100 kV.

### 3.5. General Procedure for Synthesis of 3-Oxobutanoates ***4a***,***b***

A mixture of alcohol **2a**,**b** (1.0 eq) and 2,2,6-trimethyl-4*H*-1,3-dioxin-4-one (**3**, 2.0 eq) in toluene was refluxed for 20 h. Reaction progress was monitored by TLC and visualized using iodine vapor staining. After completion, the mixture was concentrated under reduced pressure, and the crude product was purified by flash column chromatography using a gradient of ethyl acetate (EtOAc) in petroleum ether (PE) to afford the desired 3-oxobutanoates **4a**,**b**.

*2-Ethylhexyl 3-oxobutanoate* (**4a**): Following the general procedure, 2-ethylhexan-1-ol (**2a**, 2.0 g, 15.2 mmol, 2.4 mL), 2,2,6-trimethyl-4*H*-1,3-dioxin-4-one (**3**, 4.32 g, 30.4 mmol, 4.0 mL), and toluene (15 mL) were used. Compound **4a** (2.31 g, 71%) was obtained as a colorless oil after purification by flash column chromatography using a gradient of EtOAc in PE (0 → 15%). ^1^H-NMR (400 MHz, CDCl_3_) δ 4.08–4.03 (m, 2H), 3.45 (s, 2H), 2.27 (s, 3H), 1.61–1.56 (m, 1H), 1.37–1.26 (m, 8H), 0.91–0.87 (m, 6H) ppm. ^1^H-NMR spectrum data were in agreement with data reported in the literature [28].

*2-Hexyldecyl 3-oxobutanoate* (**4b**): Following the general procedure, 2-hexyldecan-1-ol (**2b**, 1.5 g, 6.2 mmol, 1.79 mL), 2,2,6-trimethyl-4*H*-1,3-dioxin-4-one (**3**, 1.76 g, 12.3 mmol, 1.63 mL), and toluene (10 mL) were used. Compound **4b** (1.57 g, 78%) was obtained as a colorless oil after purification by flash column chromatography using a gradient of EtOAc in PE (0 → 10%). ^1^H-NMR (400 MHz, DMSO-*d*_6_) δ 3.96 (d, *J* = 5.7 Hz, 2H), 3.58 (s, 2H), 2.17 (s, 3H), 1.62–1.55 (m, 1H), 1.24 (s, 24H), 0.88–0.83 (m, 6H) ppm. ^13^C-NMR (101 MHz, DMSO-*d*_6_) δ 201.4, 167.3, 66.8, 49.6, 36.6, 31.3, 31.2, 30.4, 30.3, 30.1, 29.3, 28.9, 28.8, 28.6, 25.9, 22.1, 22.0, 13.9 ppm.

### 3.6. General Procedure for Synthesis of 1,4-Dihydropyridines ***7a***,***b***

A mixture of 3-oxobutanoate **4a**,**b** (2.0 eq), benzaldehyde (**5**, 1.1 eq), and ammonium acetate (1.1 eq) in EtOH was heated at 100 °C in a sealed pressure vessel for 48 h. Reaction progress was monitored by TLC. After completion, the mixture was concentrated under reduced pressure, and the crude product was purified by flash column chromatography using a gradient of EtOAc in PE (0 → 20%) to yield 1,4-dihydropyridines **7a**,**b**.

*Bis(2-ethylhexyl) 2,6-dimethyl-4-phenyl-1,4-dihydropyridine-3,5-dicarboxylate* (**7a**): Following the general procedure, 2-ethylhexyl 3-oxobutanoate (**4a**, 1.5 g, 6.9 mmol), benzaldehyde (**5**, 403 mg, 3.8 mmol, 386 μL), ammonium acetate (**6**, 293 mg, 3.8 mmol), and EtOH (55 mL) were used. Compound **7a** (1.04 g, 60%) was obtained as a pale yellow oil. ^1^H-NMR (400 MHz, CDCl_3_) δ 7.28–7.25 (m, 2H, overlaps with CDCl_3_), 7.21–7.16 (m, 2H), 7.14–7.08 (m, 1H), 5.57 (br s, 1H), 5.03 (s, 1H), 4.04–3.89 (m, 4H), 2.35 (s, 6H), 1.54–1.50 (m, 2H), 1.37–1.30 (m, 4H), 1.29–1.25 (m, 12H), 0.90–0.82 (m, 12H) ppm. ^13^C-NMR (101 MHz, CDCl_3_) δ 168.0, 147.5, 144.2, 144.1, 128.1, 127.8, 126.3, 104.4, 104.4, 66.2, 66.1, 39.3, 39.1, 39.0, 30.6, 29.1, 29.0, 24.1, 24.0, 23.1, 19.8, 14.2, 11.2, 11.1 ppm. HRMS (TOF MS ES+): Calculated [C_31_H_47_NO_4_ + H]^+^ 498.3583; found: 498.3576.

*Bis(2-hexyldecyl) 2,6-dimethyl-4-phenyl-1,4-dihydropyridine-3,5-dicarboxylate* (**7b**): Following the general procedure, 2-hexyldecyl 3-oxobutanoate (**4b**, 1.5 g, 4.6 mmol), benzaldehyde (**5**, 268 mg, 2.5 mmol, 257 μL), ammonium acetate (**6**, 193 mg, 2.5 mmol), and EtOH (30 mL) were used. Compound **7b** (848 mg, 53%) was obtained as a pale yellow oil. ^1^H-NMR (400 MHz, CDCl_3_) δ 7.28–7.25 (m, 2H, overlaps with CDCl_3_), 7.20–7.16 (m, 2H), 7.12–7.08 (m, 1H), 5.64 (br s, 1H), 5.03 (s, 1H), 4.02–3.89 (m, 4H), 2.34 (s, 6H), 1.61–1.56 (m, 2H), 1.32–1.22 (m, 48H), 0.90–0.86 (m, 12H) ppm. ^13^C-NMR (101 MHz, CDCl_3_) δ 168.0, 147.5, 144.2, 128.1, 127.8, 126.3, 104.3, 66.5, 39.3, 37.6, 32.1, 32.0, 31.5, 31.4, 30.2, 30.1, 29.8, 29.7, 29.5, 26.9, 26.9, 26.9, 26.8, 22.8, 19.8, 14.3 ppm. HRMS (TOF MS ES+): Calculated [C_47_H_79_NO_4_ − 2H + H]^+^ 720.5931; found: 720.5931.

### 3.7. General Procedure for Synthesis of 2,6-bis(Bromomethyl)-1,4-Dihydropyridines ***9a***,***b***

To a stirred solution of compound **7a**,**b** (1.0 eq) in EtOAc, a solution of pyridinium bromide–perbromide (**8**, 2.0 eq) in EtOAc was added dropwise at r.t. The reaction mixture was stirred at r.t. for 30 min, and completion of the reaction was monitored by TLC. The reaction mixture was concentrated under reduced pressure, and the crude product was purified by flash column chromatography using a gradient of EtOAc in PE (0 → 10%) to yield compounds **9a**,**b**.

*Bis(2-ethylhexyl) 2,6-bis(bromomethyl)-4-phenyl-1,4-dihydropyridine-3,5-dicarboxylate* (**9a**): Following the general procedure, 1,4-DHP **7a** (780 mg, 1.56 mmol) in EtOAc (120 mL) and pyridinium bromide–perbromide (**8**, 1.0 g, 3.13 mmol) in EtOAc (80 mL) were used. Compound **9a** (830 mg, 83%) was obtained as a brown oil. ^1^H-NMR (400 MHz, CDCl_3_) δ 7.25–7.14 (m, 5H, overlaps with CDCl_3_), 6.56 (br s, 1H), 5.05 (s, 1H), 4.96–4.91 (dm, AB-system, *J* = 11.5 Hz, 2H), 4.63 (dt, AB-system, *J* = 11.5, 2.4 Hz, 2H), 4.09–3.95 (m, 4H), 1.59–1.53 (m, 2H), 1.40–1.31 (m, 4H), 1.29–1.20 (m, 12H), 0.91–0.84 (m, 12H) ppm. ^13^C-NMR (101 MHz, CDCl_3_) δ 166.6, 145.5, 142.0, 141.9, 128.4, 127.9, 127.1, 106.2, 106.2, 67.1, 66.9, 40.0, 39.9, 39.8, 39.0, 38.9, 30.6, 29.1, 29.0, 27.5, 24.0, 23.9, 23.1, 14.2, 11.2, 11.1 ppm. HRMS (TOF MS ES+): Calculated [C_31_H_45_NO_4_Br_2_ + H]^+^ 654.1794; found: 654.1784.

*Bis(2-hexyldecyl) 2,6-bis(bromomethyl)-4-phenyl-1,4-dihydropyridine-3,5-dicarboxylate* (**9b**): Following the general procedure, 1,4-DHP **7b** (850 mg, 1.18 mmol) in EtOAc (100 mL) and pyridinium bromide-perbromide (**8**, 752 mg, 2.36 mmol) in EtOAc (60 mL) were used. Compound **9b** (870 mg, 84%) was obtained as a pale yellow oil. ^1^H-NMR (600 MHz, CDCl_3_) δ 7.25–7.14 (m, 5H, overlaps with CDCl_3_), 6.49 (br s, 1H), 5.05 (s, 1H), 4.94 (d, AB-system, *J* = 11.5 Hz, 2H) and 4.64 (d, AB-system, *J* = 11.5 Hz, 2H), 4.00 (qd, *J* = 11.1, 5.4 Hz, 4H), 1.63–1.59 (m, 2H), 1.31–1.21 (m, 48H), 0.90–0.86 (m, 12H) ppm. ^13^C-NMR (151 MHz, CDCl_3_) δ 166.6, 145.4, 141.8, 128.5, 127.8, 127.1, 106.2, 67.3, 40.0, 37.5, 32.1, 32.0, 32.1, 31.5, 31.4, 30.2, 30.1, 29.8, 29.5, 27.8, 26.9, 26.9, 26.8, 26.7, 22.9, 22.8 14.3, 14.2 ppm. HRMS (TOF MS ES^+^): Calculated [C_47_H_77_NO_4_Br_2_ + Na]^+^ 900.4117; found: 900.4106.

### 3.8. General Procedure for Synthesis of 1,4-Dihydropyridine Amphiphiles ***14a***,***b***, ***15a***,***b***, ***16a***,***b***, and ***17a***,***b***

To a stirred solution of 2,6-bis(bromomethyl)-1,4-dihydropyridine **9a**,**b** (1.0 eq) in acetone, the corresponding pyridine derivative **10–13** (2.0 eq) was added, and the resulting mixture was stirred at r.t. The reaction progress was monitored by TLC. After completion, the mixture was cooled to 4 °C, and the precipitates were filtered off.

*1,1′-((3,5-Bis(((2-ethylhexyl)oxy)carbonyl)-4-phenyl-1,4-dihydropyridine-2,6-diyl)bis(methylene))bis(pyridin-1-ium) dibromide* (**14a**): Following the general procedure, bromide **9a** (50 mg, 0.08 mmol), pyridine (**10**, 12 mg, 0.15 mmol, 12 μL), and acetone (3 mL) were stirred for 24 h. Compound **14a** (14 mg, 22%) was obtained as a white solid with m.p. 172–175 °C (decomp.). ^1^H-NMR (400 MHz, CDCl_3_) δ 9.33–9.29 (m, 4H), 8.67 (t, *J* = 7.7 Hz, 2H), 8.22 (t, *J* = 7.7 Hz, 4H), 7.27–7.18 (m, 5H, overlaps with CDCl_3_), 6.36 (d, AB-system, *J* = 13.8 Hz, 2H), 5.96 (d, AB-system, *J* = 13.8 Hz, 2H), 5.05 (s, 1H), 4.10–3.89 (m, 4H), 1.57–1.52 (m, 2H), 1.38–1.30 (m, 4H), 1.28–1.19 (m, 12H), 0.91–0.81 (m, 12H) ppm. ^13^C-NMR (151 MHz, CDCl_3_) 166.8, 147.0, 144.9, 138.5, 129.1, 128.9, 127.9, 127.8, 110.3, 67.9, 67.8, 57.4, 39.5, 38.9, 38.8, 30.4, 30.4, 29.1, 29.0, 23.9, 23.7, 23.1, 14.2, 11.1, 11.0 ppm. HRMS (TOF MS ES^+^): Calculated [C_41_H_55_N_3_O_4_]^2+^ 326.7091; found: 326.7106.

*1,1′-((3,5-Bis(((2-hexyldecyl)oxy)carbonyl)-4-phenyl-1,4-dihydropyridine-2,6-diyl)bis(methylene))bis(pyridin-1-ium) dibromide* (**14b**): Following the general procedure, bromide **9b** (80 mg, 0.09 mmol), pyridine (**10**, 14 mg, 0.18 mmol, 14 μL), and acetone (4 mL) were stirred for 24 h. Compound **14b** (67 mg, 70%) was obtained as a yellow solid with m.p. 178–180 °C. ^1^H-NMR (600 MHz, DMSO-*d*_6_) δ 10.38 (br s, 1H), 8.98 (d, *J* = 6.3 Hz, 4H), 8.59 (t, *J* = 7.7 Hz, 2H), 8.11 (t, *J* = 7.7 Hz, 4H), 7.28–7.19 (m, 5H), 6.08 (d, AB-system, *J* = 14.5 Hz, 2H) and 5.63 (d, AB-system, *J* = 14.5 Hz, 2H), 5.03 (s, 1H), 3.97–3.88 (m, 4H), 1.57–1.51 (m, 2H), 1.28–1.14 (m, 48H), 0.88–0.83 (m, 12H) ppm. ^13^C-NMR (151 MHz, DMSO-*d*_6_) δ 165.9, 146.4, 144.7, 139.1, 128.3, 127.9, 127.3, 108.0, 66.8, 57.7, 36.7, 31.4, 31.3, 31.2, 30.8, 30.7, 30.6, 30.5, 29.3, 29.1, 29.0, 28.7, 26.3, 26.2, 26.1, 26.0, 22.1, 13.9 ppm. HRMS (TOF MS ES^+^): Calculated [C_57_H_87_N_3_O_4_]^2+^ 438.8343; found: 438.8357.

*1,1′-((3,5-Bis(((2-ethylhexyl)oxy)carbonyl)-4-phenyl-1,4-dihydropyridine-2,6-diyl)bis(methylene))bis(4-methylpyridin-1-ium) dibromide* (**15a**): Following the general procedure, bromide **9a** (100 mg, 0.15 mmol), 4-methylpyridine (11, 28 mg, 0.31 mmol, 30 μL), and acetone (5 mL) were stirred for 24 h. Compound **15a** (84 mg, 68%) was obtained as a yellow solid with m.p. 194–196 °C (decomp.). ^1^H-NMR (600 MHz, DMSO-*d*_6_) δ 10.39 (br s, 1H), 8.82 (d, *J* = 6.6 Hz, 4H), 7.94 (d, *J* = 6.6 Hz, 4H), 7.29–7.20 (m, 5H), 6.00 (d, AB-system, *J* = 14.8 Hz, 2H), 5.54 (d, AB-system, *J* = 14.8 Hz, 2H), 5.02 (s, 1H), 4.00–3.89 (m, 4H), 2.61 (s, 6H), 1.49–1.45 (m, 2H), 1.27–1.15 (m, 16H), 0.85–0.76 (m, 12H) ppm. ^13^C-NMR (151 MHz, DMSO-*d*_6_) 165.8, 159.8, 143.6, 139.1, 128.4, 128.1, 127.4, 127.2, 108.1, 66.4, 66.2, 56.9, 38.2, 38.2, 29.8, 28.4, 28.3, 23.3, 23.1, 22.4, 22.3, 21.4, 13.9, 13.8, 10.9, 10.8 ppm. HRMS (TOF MS ES^+^): Calculated [C_43_H_59_N_3_O_4_]^2+^ 340.7247; found: 340.7253.

*1,1′-((3,5-Bis(((2-hexyldecyl)oxy)carbonyl)-4-phenyl-1,4-dihydropyridine-2,6-diyl)bis(methylene))bis(4-methylpyridin-1-ium) dibromide* (**15b**): Following the general procedure, bromide **9b** (80 mg, 0.09 mmol), 4-methylpyridine (**11**, 17 mg, 0.18 mmol, 18 μL), and acetone (4 mL) were stirred for 24 h. Compound **15b** (70 mg, 72%) was obtained as a pale yellow solid with m.p. 179–181 °C. ^1^H-NMR (600 MHz, DMSO-*d*_6_) δ 10.53 (br s, 1H), 8.85–8.84 (m, 4H), 7.95 (d, *J* = 6.8 Hz, 4H), 7.29–7.19 (m, 5H), 6.01 (d, AB-system, *J* = 14.6 Hz, 2H), 5.59 (d, AB-system, *J* = 14.6 Hz, 2H), 5.01 (s, 1H), 3.96–3.86 (m, 4H), 2.62 (s, 6H), 1.56–1.53 (m, 2H), 1.28–1.14 (m, 48H), 0.87–0.83 (m, 12H) ppm. ^13^C-NMR (151 MHz, DMSO-*d*_6_) δ 165.9, 159.8, 145.2, 143.7, 139.3, 128.3, 128.1, 127.3, 127.2, 107.9, 66.7, 56.8, 36.7, 31.3, 31.3, 31.2, 30.8, 30.7, 30.6, 30.5, 29.3, 29.0, 28.7, 26.3, 26.2, 26.1, 26.0, 22.1, 21.4, 13.9 ppm. HRMS (TOF MS ES+): Calculated [C_59_H_91_N_3_O_4_]^2+^ 452.8499; found: 452.8513.

*1,1′-((3,5-Bis(((2-ethylhexyl)oxy)carbonyl)-4-phenyl-1,4-dihydropyridine-2,6-diyl)bis(methylene))bis(4-(dimethylamino)pyridin-1-ium) dibromide* (**16a**): Following the general procedure, bromide **9a** (100 mg, 0.15 mmol), 4-dimethylaminopyridine (**13**, 37 mg, 0.30 mmol), and acetone (5 mL) were stirred for 2 h. Compound **16a** (98 mg, 71%) was obtained as a white solid with m.p. 217–219 °C (decomp.). ^1^H-NMR (300 MHz, CDCl_3_) δ 10.71 (br s, 1H), 8.34–8.32 (m, 4H), 7.29–7.26 (m, 2H, overlaps with CDCl_3_), 7.23–7.19 (m, 3H), 7.02 (d, *J* = 7.4 Hz, 4H), 5.86–5.83 (ddm, AB-system, *J* = 13.6, 4.7 Hz, 2H), 5.48–5.44 (dt, AB-system, *J* = 13.6, 4.7 Hz, 2H) 5.00 (s, 1H), 4.10–3.99 (m, 2H), 3.94–3.87 (m, 2H), 3.34 (s, 12H), 1.60–1.56 (m, 2H), 1.39–1.36 (m, 4H), 1.31–1.22 (m, 12H), 0.90–0.86 (m, 12H) ppm. ^13^C-NMR (151 MHz, CDCl_3_) δ 166.9, 156.7, 145.7, 141.8, 139.9, 128.8, 128.0, 127.9, 127.8, 127.6, 109.2, 109.1, 108.5, 67.6, 67.4, 53.4, 41.3, 39.4, 38.9, 38.8, 30.5, 30.4, 29.1, 29.0, 24.0, 23.8, 23.1, 14.3, 14.2, 11.2, 11.1 ppm. HRMS (TOF MS ES+): Calculated [C_45_H_65_N_5_O_4_]^2+^ 369.7513; found: 369.7513.

*1,1′-((3,5-Bis(((2-hexyldecyl)oxy)carbonyl)-4-phenyl-1,4-dihydropyridine-2,6-diyl)bis(methylene))bis(4-(dimethylamino)pyridin-1-ium) dibromide* (**16b**): Following the general procedure, bromide **9b** (30 mg, 0.03 mmol), 4-dimethylaminopyridine (**13**, 9 mg, 0.07 mmol), and acetone (2 mL) were stirred for 2 h. Product **16b** (20 mg, 60%) was obtained as a pale yellow solid with m.p. 181–183 °C (decomp.). ^1^H-NMR (600 MHz, DMSO-*d*_6_) δ 10.29 (br s, 1H), 8.21 (d, *J* = 7.6 Hz, 4H), 7.23–7.18 (m, 5H), 6.97 (d, *J* = 7.6 Hz, 4H), 5.59 (d, AB-system, *J* = 14.6 Hz, 2H), 5.23 (d, AB-system, *J* = 14.6 Hz, 2H), 5.00 (s, 1H), 3.99–3.96 (m, 2H), 3.89–3.86 (m, 2H), 3.18 (s, 12H), 1.59–1.54 (m, 2H), 1.27–1.16 (m, 48H), 0.86–0.83 (m, 12H) ppm. ^13^C-NMR (151 MHz, DMSO-*d*_6_) δ 166.0, 155.9, 141.8, 128.3, 127.1, 107.4, 66.5, 36.7, 31.3, 31.3, 31.2, 30.8, 30.8, 30.7, 30.6, 29.4, 29.3, 29.0, 29.0, 28.7, 28.6, 26.2, 26.1, 26.0, 22.1, 13.9 ppm. HRMS (TOF MS ES+): Calculated [C_61_H_97_N_5_O_4_]^2+^ 481.8765; found: 481.8769.

*1,1′-((3,5-Bis(((2-ethylhexyl)oxy)carbonyl)-4-phenyl-1,4-dihydropyridine-2,6-diyl)bis(methylene))bis(4-phenylpyridin-1-ium) dibromide* (**17a**): Following the general procedure, bromide **9a** (100 mg, 0.15 mmol), 4-phenylpyridine (**12**, 47 mg, 0.30 mmol), and acetone (5 mL) were stirred for 24 h. Compound **17a** (79 mg, 54%) was obtained as a yellow solid with m.p. 178–180 °C (decomp.). ^1^H-NMR (300 MHz, CDCl_3_) δ 10.85 (br s, 1H), 9.20 (d, *J* = 6.6 Hz, 4H), 8.34 (d, *J* = 6.6 Hz, 4H), 7.78 (d, *J* = 7.6 Hz, 4H), 7.54–7.19 (m, 11H, overlaps with CDCl_3_), 6.43 (d, AB-system, *J* = 13.8 Hz, 2H), 5.80 (d, AB-system, *J* = 13.8 Hz, 2H), 5.18 (s, 1H), 4.11–3.94 (m, 4H), 1.62–1.53 (m, 2H), 1.40–1.20 (m, 16H), 0.90–0.84 (m, 12H) ppm. ^13^C-NMR (151 MHz, CDCl_3_) δ 166.0, 155.9, 141.8, 128.3, 122.1, 107.4, 66.5, 53.8, 48.6, 36.7, 31.3, 31.2, 30.8, 30.7, 30.6, 29.3, 29.0, 28.7, 26.2, 26.1, 22.1, 13.9 ppm. HRMS (TOF MS ES+): Calculated [C_53_H_63_N_3_O_4_]^2+^ 402.7404; found: 402.7420.

*1,1′-((3,5-Bis(((2-hexyldecyl)oxy)carbonyl)-4-phenyl-1,4-dihydropyridine-2,6-diyl)bis(methylene))bis(4-phenylpyridin-1-ium) dibromide* (**17b**): Following the general procedure, bromide **9b** (80 mg, 0.09 mmol), 4-phenylpyridine (**12**, 28 mg, 0.18 mmol), and acetone (4 mL) were stirred for 24 h. Compound **17b** (70 mg, 68%) was obtained as a white solid with m.p. 179–181 °C. ^1^H-NMR (400 MHz, DMSO-*d*_6_) δ 10.42 (br s, 1H), 9.01 (d, *J* = 6.6 Hz, 4H), 8.44 (d, *J* = 6.6 Hz, 4H), 7.93 (d, *J* = 7.3 Hz, 4H), 7.67–7.62 (m, 2H), 7.56–7.52 (m, 4H), 7.34–7.21 (m, 5H), 6.17 (d, AB-system, *J* = 14.9 Hz, 2H), 5.60 (d, AB-system, *J* = 14.9 Hz, 2H), 5.07 (s, 1H), 4.01–3.93 (m, 4H), 1.60–1.52 (m, 2H), 1.27–1.14 (m, 48H), 0.86–0.81 (m, 12H) ppm. ^13^C-NMR (101 MHz, DMSO-*d*_6_) δ 166.0, 155.3, 144.8, 139.0, 133.0, 132.4, 129.6, 128.3, 128.0, 127.3, 124.1, 108.4, 66.8, 56.7, 36.7, 31.2, 31.2, 30.8, 30.7, 30.6, 30.5, 29.3, 29.0, 28.9, 28.7, 26.3, 26.1, 26.1, 22.1, 13.9, 13.8 ppm. HRMS (TOF MS ES+): Calculated [C_69_H_95_N_3_O_4_]^2+^ 514.8659; found: 514.8667.

## 4. Conclusions

A series of amphiphilic 1,4-dihydropyridine (1,4-DHP) derivatives bearing branched ester moieties at the 3,5-positions and cationic pyridinium substituents at the 2,6-positions was synthesized. The target compounds were obtained via Hantzsch cyclization, bromination of the 2,6-dimethyl groups, and subsequent bromine nucleophilic substitution with selected pyridine derivatives.

The self-assembling properties of the prepared amphiphiles in aqueous medium were evaluated by dynamic light scattering (DLS). All studied derivatives formed positively charged nanoparticles with average diameters ranging from 52 to 439 nm and PDI values between 0.194 and 0.527. The results demonstrated a clear influence of the hydrophobic ester chains on nanoparticle size and homogeneity. In particular, amphiphiles containing 2-hexyldecyl ester chains formed smaller and more uniform nanoparticles compared to the corresponding 2-ethylhexyl analogues. Several derivatives also exhibited good colloidal stability over 14 days of storage at r.t.

Computational evaluation of AlogP, PSA, and pK_a_ values provided additional insight into the structure–property relationships. Extension of the branched ester chains significantly increased lipophilicity, whereas PSA values were mainly influenced by the structure of the polar headgroup. In particular, comparison of 2-ethylhexyl-substituted derivatives with their 2-hexyldecyl analogs revealed an approximately 7-unit increase in AlogP for compounds bearing identical cationic moieties, reflecting the substantial contribution of the longer branched alkyl chains to overall hydrophobicity. The calculated pK_a_ values confirm that the quaternized headgroups remain positively charged, ranging from 7.20 to 10.99. Introduction of cationic moieties at the 2,6-positions of the 1,4-DHP ring significantly increases the acidity of the NH proton compared to 1,4-DHP derivatives bearing 2,6-dimethyl substituents. The results indicate that hydrophobic volume and branching architecture at the 3,5-positions represent dominant determinants of nanoparticle dimensions within this scaffold, while electrostatic stabilization arises primarily from the nature of the cationic substituents. These findings establish the 1,4-DHP framework as a controllable model system for investigating how branched hydrophobic domains govern amphiphile self-assembly, thereby extending structure–assembly principles beyond conventional glycerol-based lipid platforms.

## Figures and Tables

**Table 1:** Nucleophilic substitution of bromine in 2,6-bis(bromomethyl)-1,4-DHPs **9a,b** with pyridines **10–13:** reagents, reaction times, and yields of amphiphiles **14a**,**b**, **15a**,**b**, **16a**,**b**, and **17a**,**b**.

Entry	1,4-DHP	R^1^	R^2^	Py	Time, h	Product	Yield, %
1	**9a**	C_2_H_5_	C_4_H_9_	**10**	24	**14a**	22
2	**9b**	C_6_H_13_	C_8_H_17_	**10**	24	**14b**	70
3	**9a**	C_2_H_5_	C_4_H_9_	**11**	24	**15a**	68
4	**9b**	C_6_H_13_	C_8_H_17_	**11**	24	**15b**	72
5	**9a**	C_2_H_5_	C_4_H_9_	**12**	2	**16a**	71
6	**9b**	C_6_H_13_	C_8_H_17_	**12**	2	**16b**	60
7	**9a**	C_2_H_5_	C_4_H_9_	**13**	24	**17a**	54
8	**9b**	C_6_H_13_	C_8_H_17_	**13**	24	**17b**	68

**Table 2:** Values of zeta potential (Z_pot_) of nanoparticles formed by 1,4-DHP amphiphiles **1**, **14a**,**b**, **15a**,**b**, **16a**,**b**, and **17b** obtained by DLS measurements; AlogP values, polar surface area (PSA), and pKa values of 1,4-DHP amphiphiles **1** and **14a**,**b**–**17a**,**b** (for cations).

Comp.	1	14a	14b	15a	15b	16a	16b	17a	17b
Z_pot_., mV	75.4 ± 7.5 [32]	16.1 ± 0.6	43.9 ± 2.5	38.3 ± 3.8	47.7 ± 1.2	22.1 ± 1.7	40.1 ± 1.3	nd	31.0 ± 0.5
AlogP	10.97 [26]	8.98	16.28	9.96	17.26	9.31	16.61	12.02	19.32
PSA, Å^2^	70.64 [26]	72.39	72.39	72.39	72.39	78.87	78.87	72.39	72.39
pKa	~7.4 [20]	8.33	7.20	7.52	8.51	9.38 − 4.8 *	8.05 − 4.5 *	10.22	10.99

nd—not determined; *—pKa values for dimethylamino group.

## Data Availability

The original contributions presented in this study are included in the article/Supplementary Material. Further inquiries can be directed to the corresponding author.

## Supplementary Materials

The following supporting information can be downloaded at: https://www.mdpi.com/article/10.3390/molecules31071161/s1. Characterization data for products **4b**, **7a**,**b**, **9a**,**b**, **14a**,**b**, **15a**,**b**, **16a**,**b**, and **17a**,**b**, including ^1^H- and ^13^C-NMR spectra and HRMS. Figures S1 and S2 for comp. **4b**; Figures S3–S5 for comp. **7a**; Figures S6–S8 for comp. **7b**; Figures S9–S11: for comp. **9a**; Figures S12–S14 for comp. **9b**; Figures S15–S17 for comp. **14a**; Figures S18–S20 for comp. **14b**; Figures S21–S23 for comp. **15a**; Figures S24–S26 for comp. **15b**; Figures S27–S29 for comp. **16a**; Figures S30–S32 for comp. **16b**; Figures S33–S35 for comp. **17a**; Figures S36–S38 for comp. **17b**; Table S1 DLS measurement data; Figures S39–S45 DLS size distribution profiles for 1,4-DHP amphiphiles **14a**,**b**–**16a**,**b** and **17b**; Figures S46–S52 DLS zeta potential profiles for 1,4-DHP amphiphiles **14a**,**b**–**16a**,**b** and **17b**; Figure S53 TEM images of formed particles of **14a**,**b** and **15a**,**b**.
