# Supplementary material for: Design, Synthesis, and Self-Assembly of Amphiphilic 1,4-Dihydropyridines Containing Branched Ester Moieties
Source: Molecules. 2026 Mar 31;31(7):1161. doi: 10.3390/molecules31071161 (PMC13074329; doi:10.3390/molecules31071161)
Supplement: Supplementary file 1 [file molecules-31-01161-s001.zip › molecules-4201321-supplementary.pdf]

*Supplementary Materials*

# Design, Synthesis, and Self-Assembly of Amphiphilic 1,4-Dihydropyridines Containing Branched Ester Moieties

Davis Lacis[1,2], Martins Rucins[1], Nadiia Pikun[1], Ruslans Muhamadejevs[1], Karlis Pajuste[1], Mara Plotniece[2,3], Juris Jansons[4], Anna Zajakina[4], Arkadij Sobolev[1], Aiva Plotniece[1,3,*]

[1] Latvian Institute of Organic Synthesis, Aizkraukles 21, Riga LV-1006, Latvia; davis.lacis@osi.lv (D.L.), rucins@osi.lv (M.R.), nadiia@osi.lv (N.P.), muhamadejev@osi.lv (R.M.), kpajuste@osi.lv (K.P.), arkady@osi.lv (A.S.), aiva@osi.lv (A.P)

[2] Faculty of Natural Sciences and Technology, Riga Technical University, P. Valdena 3, Riga LV-1048, Latvia; mara.plotniece@rtu.lv (M.P.)

[3] Department of Applied Pharmacy, Faculty of Pharmacy, Riga Stradiņš University, Konsula 21, LV-1007 Riga, Latvia

[4] Latvian Biomedical Research and Study Centre, Ratsupites 1 k-1, Riga, LV-1067, Latvia; jansons@biomed.lu.lv (J.J.), anna@biomed.lu.lv (A.Z.)

* Correspondence: aiva@osi.lv

## Table of Contents

$^{1}$H-NMR spectrum of compound **4b** ... 2
$^{13}$C-NMR spectrumof compound **4b** ... 2
$^{1}$H-NMR spectrum of compound **7a** ... 3
$^{13}$C-NMR spectrum of compound **7a** ... 3
HRMS data of compound **7a** ... 4
$^{1}$H-NMR spectrum of compound **7b** ... 4
$^{13}$C-NMR spectrum of compound **7b** ... 5
HRMS data of compound **7b** ... 5
$^{1}$H-NMR spectrum of compound **9a** ... 6
$^{13}$C-NMR spectrum of compound **9a** ... 6
HRMS data of compound **9a** ... 7
$^{1}$H-NMR spectrum of compound **9b** ... 7
$^{13}$C-NMR spectrum of compound **9b** ... 8
HRMS data of compound **9b** ... 8
$^{1}$H-NMR spectrum of compound **14a** ... 9
$^{13}$C-NMR spectrum of compound **14a** ... 9
HRMS data of compound **14a** ... 10
$^{1}$H-NMR spectrum of compound **14b** ... 10
$^{13}$C-NMR spectrum of compound **14b** ... 11
HRMS data of compound **14b** ... 11
$^{1}$H-NMR spectrum of compound **15a** ... 12
$^{13}$C-NMR spectrum of compound **15a** ... 12
HRMS data of compound **15a** ... 13
$^{1}$H-NMR spectrum of compound **15b** ... 13
$^{13}$C-NMR spectrum of compound **15b** ... 14
HRMS data of compound **15b** ... 14
$^{1}$H-NMR spectrum of compound **16a** ... 15
$^{13}$C-NMR spectrum of compound **16a** ... 15
HRMS data of compound **16a** ... 16
$^{1}$H-NMR spectrum of compound **16b** ... 16
$^{13}$C-NMR spectrum of compound **16b** ... 17
HRMS data of compound **16b** ... 17
$^{1}$H-NMR spectrum of compound **17a** ... 18
$^{13}$C-NMR spectrum of compound **17a** ... 18
HRMS data of compound **17a** ... 19
$^{1}$H-NMR spectrum of compound **17b** ... 19
$^{13}$C-NMR spectrum of compound **17b** ... 20
HRMS data of compound **17b** ... 20
Table S1. $Z_{av}$ and PDI values for 1,4-DHP amphiphiles **1**, **14a**,**b**-**16a**,**b** and **17b** determined by DLS ... 21
DLS size distribution profile for 1,4-DHP amphiphiles **14a**,**b**-**16a**,**b** and **17b** ... 21
DLS zeta potential profile for 1,4-DHP amphiphiles **14a**,**b**-**16a**,**b** and **17b** ... 24
TEM images of formed nanostructures of **14a**,**b**; **15a**,**b** ... 26

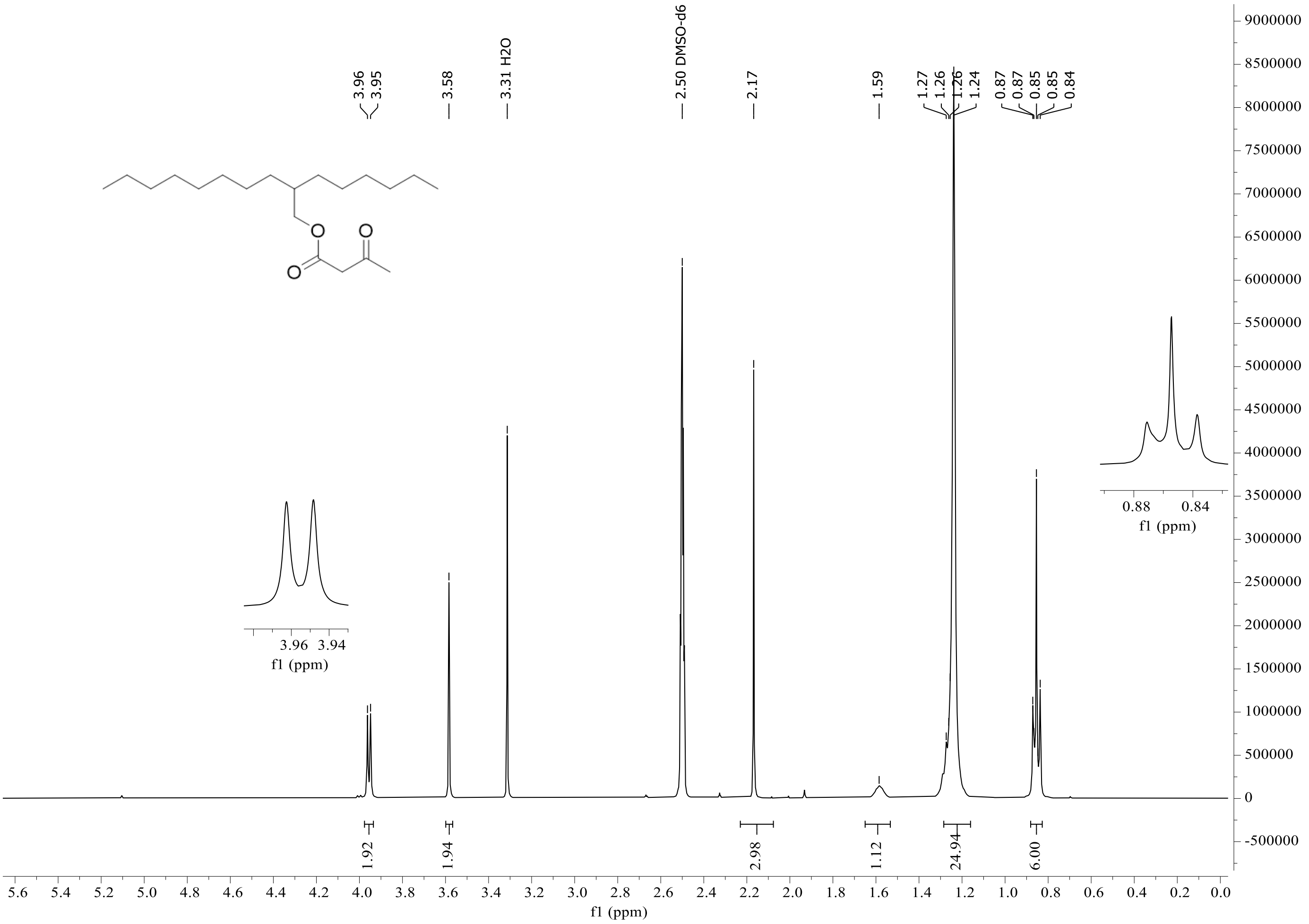


**Figure S1.** $^{1}$H NMR spectrum of tetraethyl 2-hexyldecyl 3-oxobutanoate (**4b**) in DMSO-$d_6$.

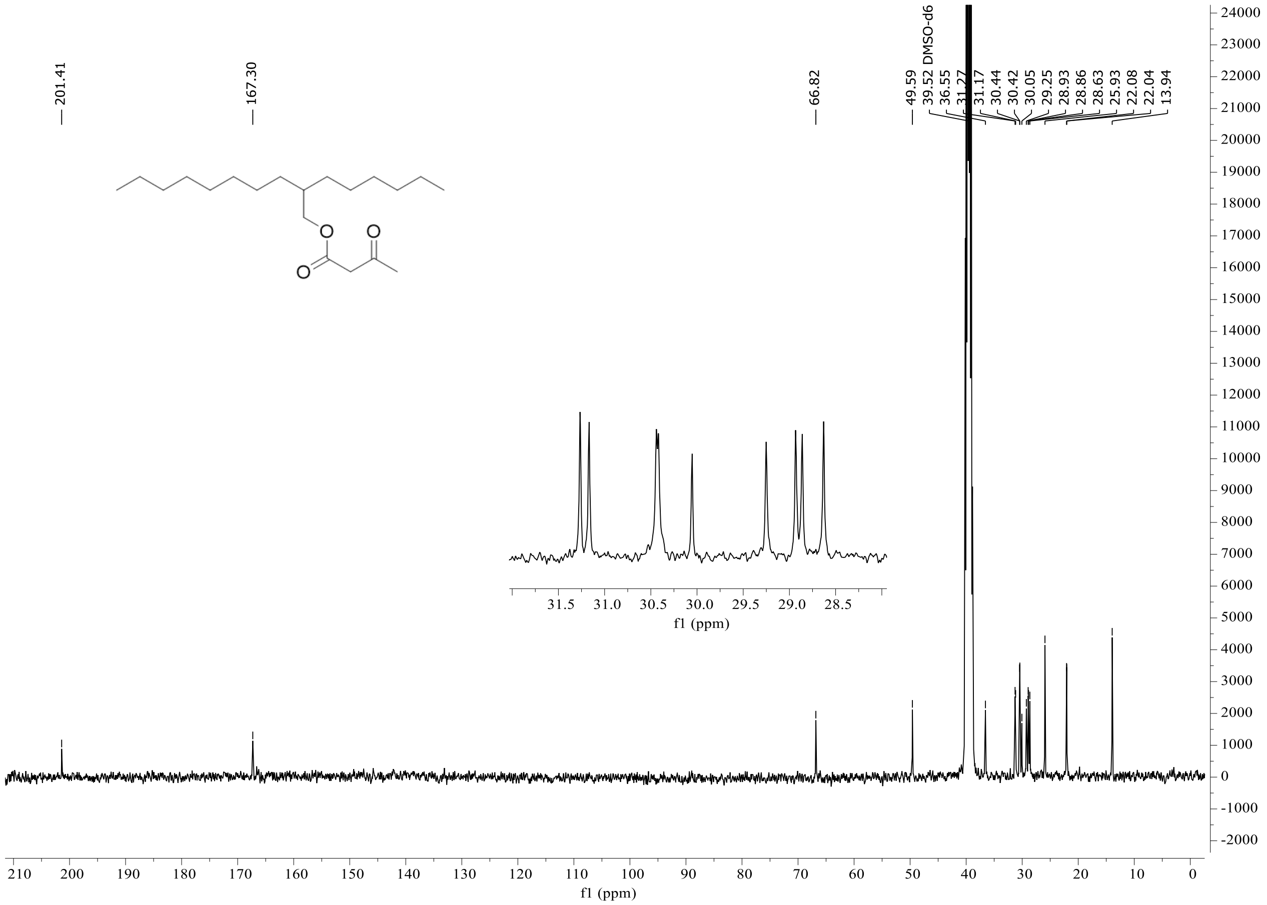


**Figure S2.** $^{13}$C NMR spectrum of 2-hexyldecyl 3-oxobutanoate (**4b**) in DMSO-$d_6$.

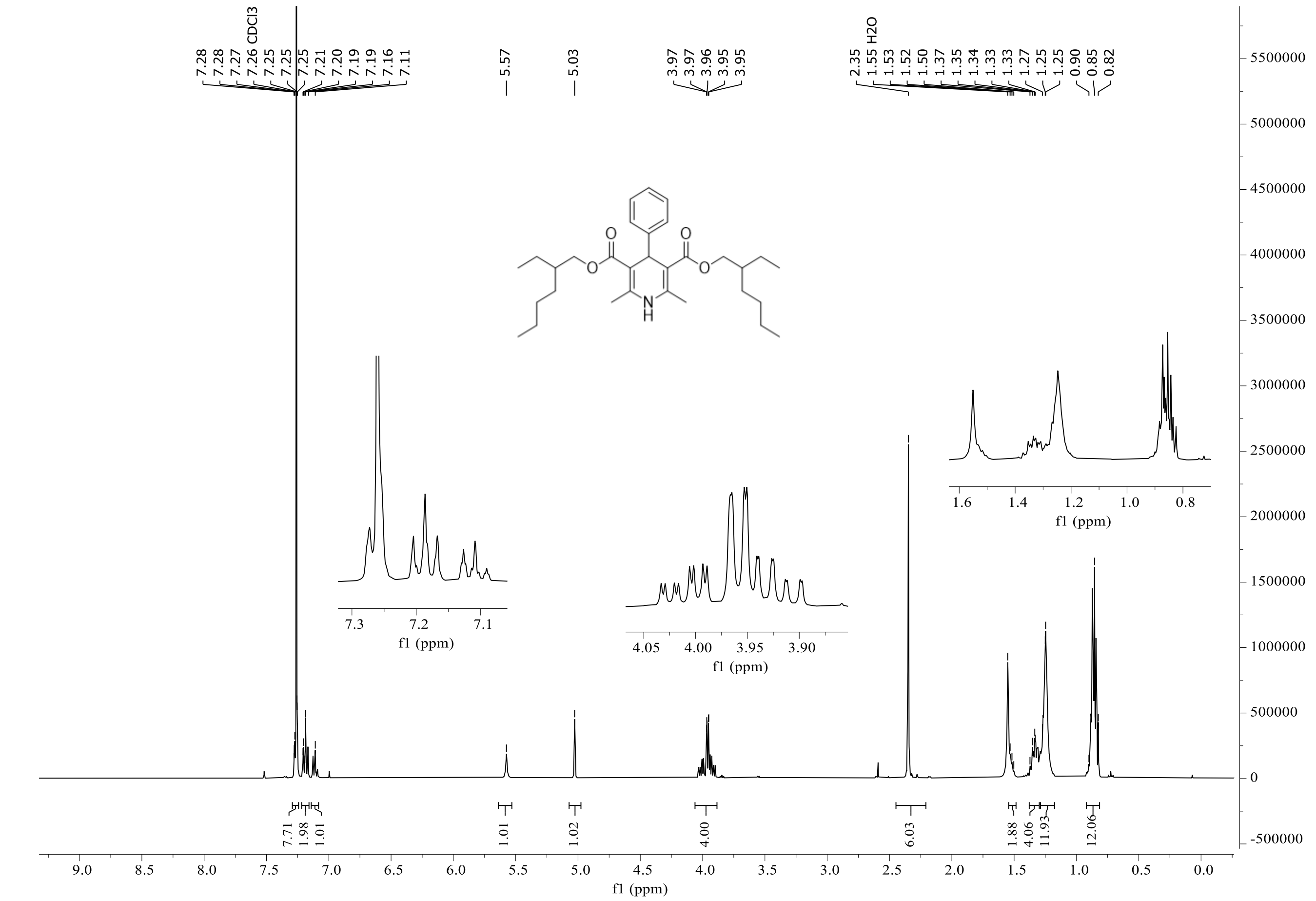


**Figure S3.** $^{1}$H-NMR spectrum of bis(2-ethylhexyl) 2,6-dimethyl-4-phenyl-1,4-dihydropyridine-3,5-dicarboxylate (**7a**) in $CDCl_3$.

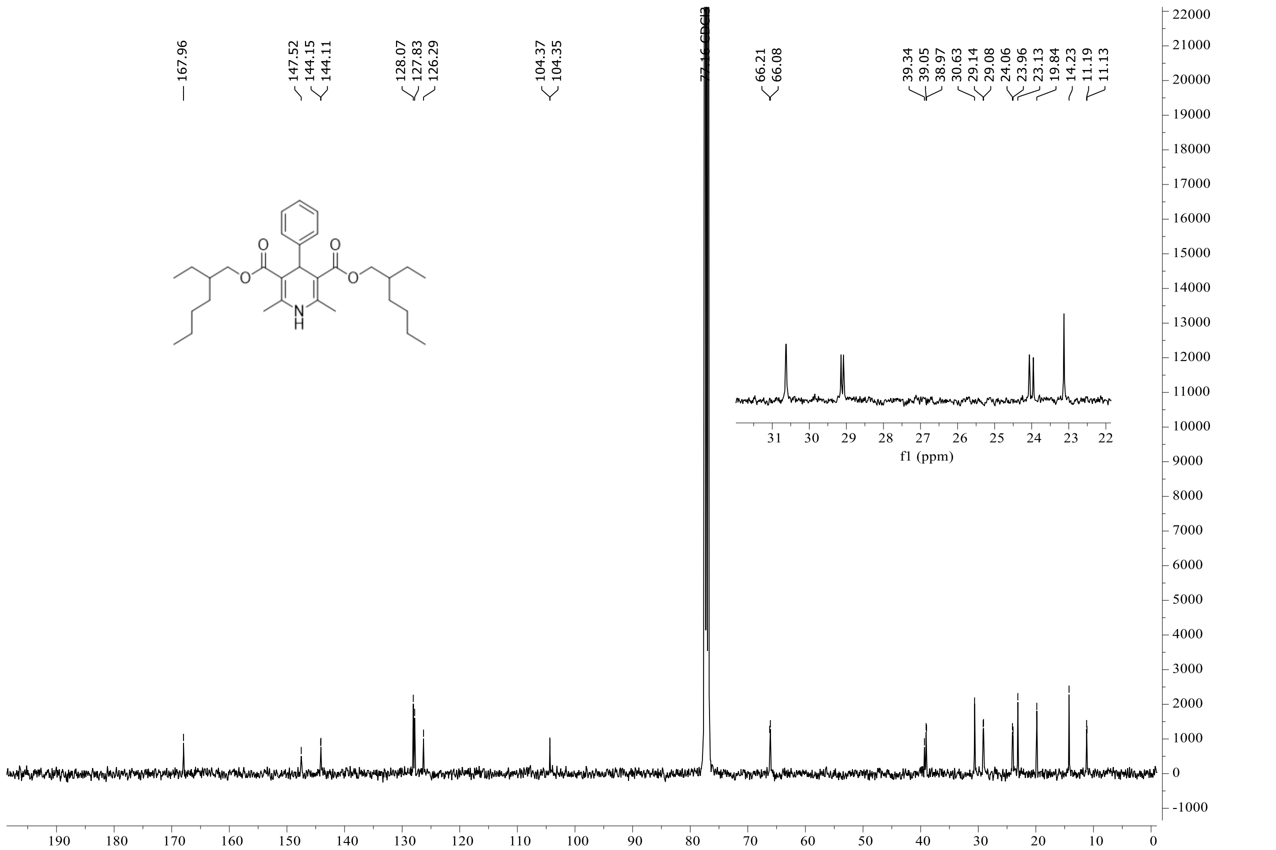


**Figure S4.** $^{13}$C-NMR spectrum of bis(2-ethylhexyl) 2,6-dimethyl-4-phenyl-1,4-dihydropyridine-3,5-dicarboxylate (**7a**) in $CDCl_3$.

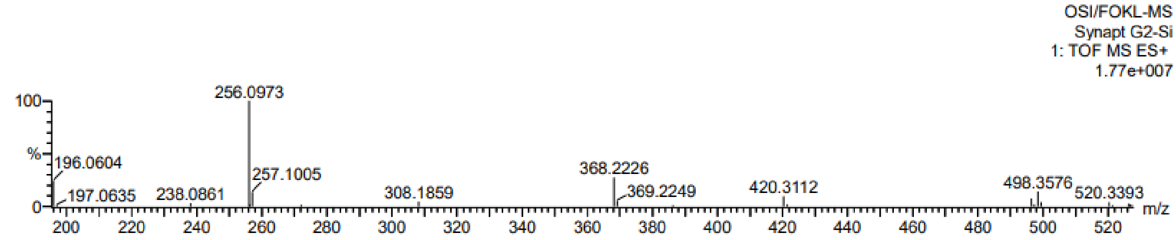


| | | | | | | | | |
|---|---|---|---|---|---|---|---|---|
| Minimum: | | | | -0.5 | | | | |
| Maximum: | | 10.0 | 3.0 | 200.0 | | | | |
| Mass | Calc. Mass | mDa | PPM | DBE | i-FIT | Norm | Conf(%) | Formula |
| 498.3576 | 498.3583 | -0.7 | -1.4 | 8.5 | 60.0 | 0.922 | 39.78 | C31 H48 N O4 |
| | 498.3588 | -1.2 | -2.4 | 1.5 | 59.7 | 0.600 | 54.88 | C16 H44 N13 O5 |
| | 498.3562 | 1.4 | 2.8 | 2.5 | 62.0 | 2.929 | 5.34 | C12 H40 N19 O3 |

**Figure S5** HRMS data of bis(2-ethylhexyl) 2,6-dimethyl-4-phenyl-1,4-dihydropyridine-3,5-dicarboxylate (**7a**).

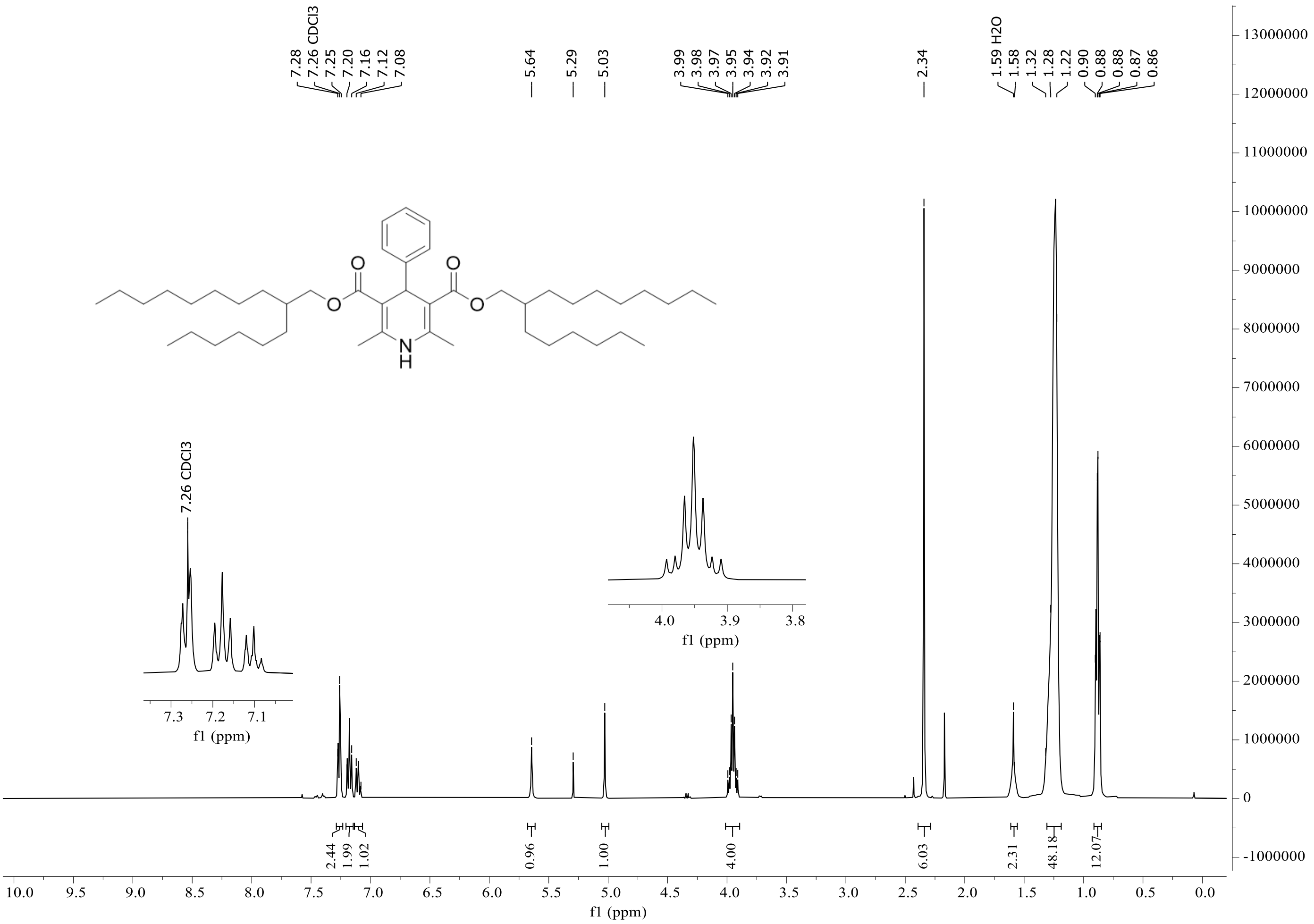


**Figure S6.** $^{1}$H-NMR spectrum of bis(2-hexyldecyl) 2,6-dimethyl-4-phenyl-1,4-dihydropyridine-3,5-dicarboxylate (**7b**) in $CDCl_3$.

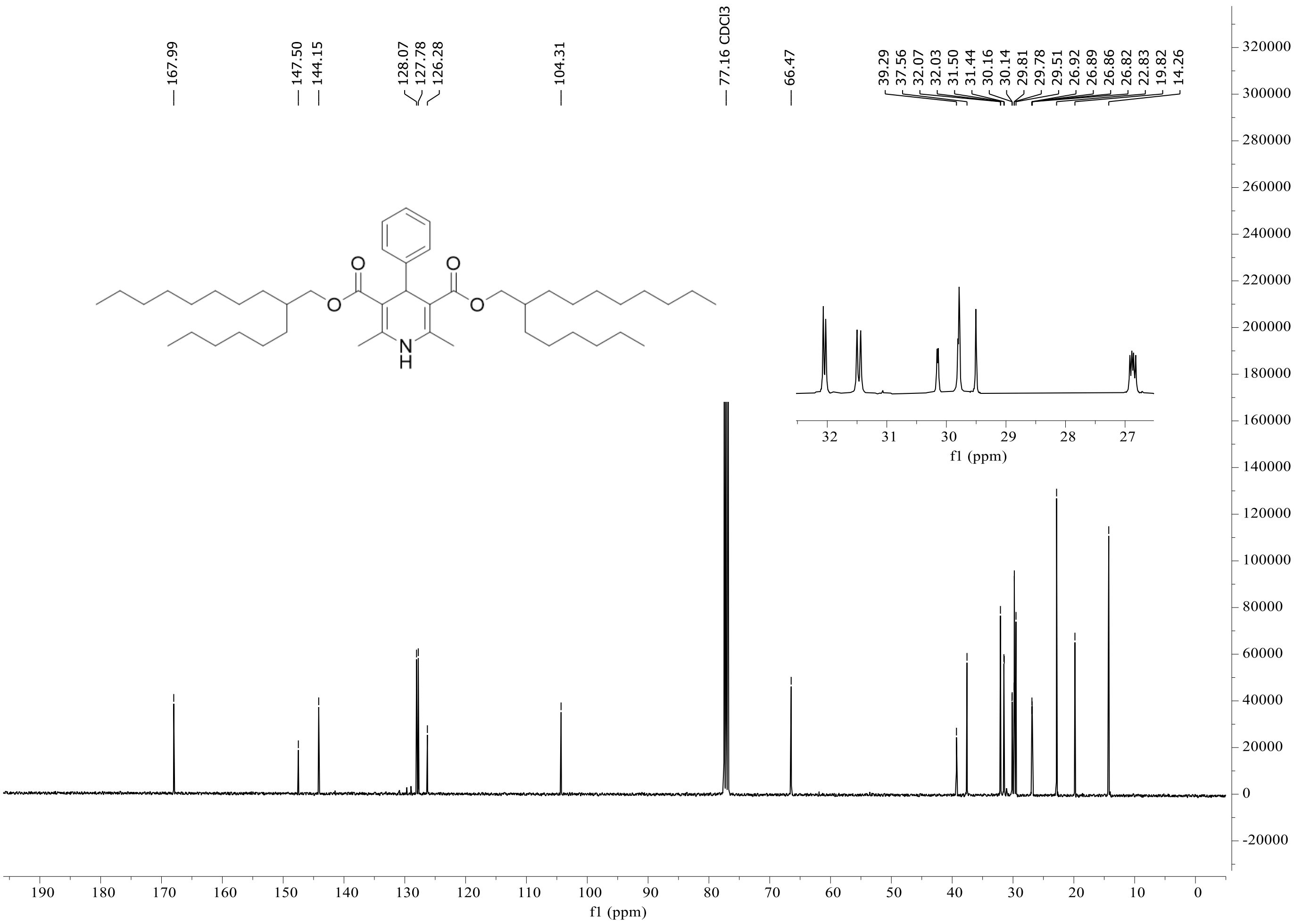


**Figure S7.** $^{13}$C-NMR spectrum of bis(2-hexyldecyl) 2,6-dimethyl-4-phenyl-1,4-dihydropyridine-3,5-dicarboxylate (**7b**) in $CDCl_3$.

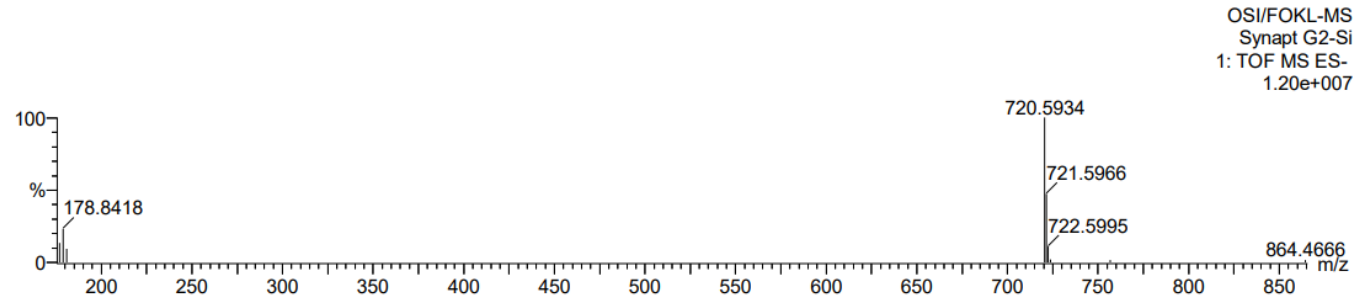


| | | | | | | | | |
|---|---|---|---|---|---|---|---|---|
| Minimum: | | | | -0.5 | | | | |
| Maximum: | | 10.0 | 3.0 | 200.0 | | | | |
| Mass | Calc. Mass | mDa | PPM | DBE | i-FIT | Norm | Conf(%) | Formula |
| 720.5934 | 720.5936 | -0.2 | -0.3 | 2.5 | 64.3 | 0.378 | 68.55 | C32 H74 N13 O5 |
| | 720.5931 | 0.3 | 0.4 | 9.5 | 65.4 | 1.433 | 23.86 | C47 H78 N O4 |
| | 720.5944 | -1.0 | -1.4 | 14.5 | 66.5 | 2.578 | 7.59 | C48 H74 N5 |

**Figure S8.** HRMS data of bis(2-hexyldecyl) 2,6-dimethyl-4-phenyl-1,4-dihydropyridine-3,5-dicarboxylate (**7b**).

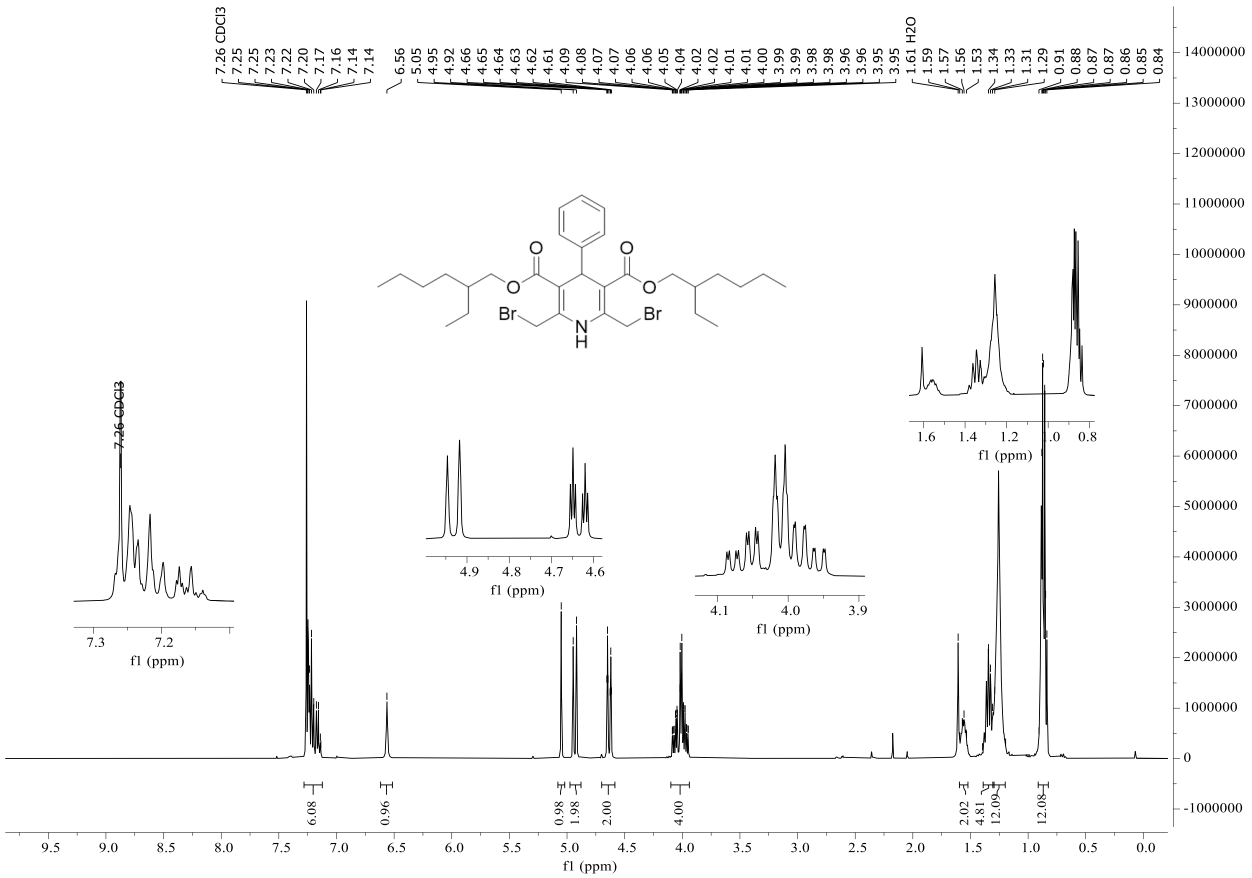

**Figure S9.** $^{1}H$-NMR spectrum of bis(2-ethylhexyl) 2,6-bis(bromomethyl)-4-phenyl-1,4-dihydropyridine-3,5-dicarboxylate (**9a**) in $CDCl_3$.

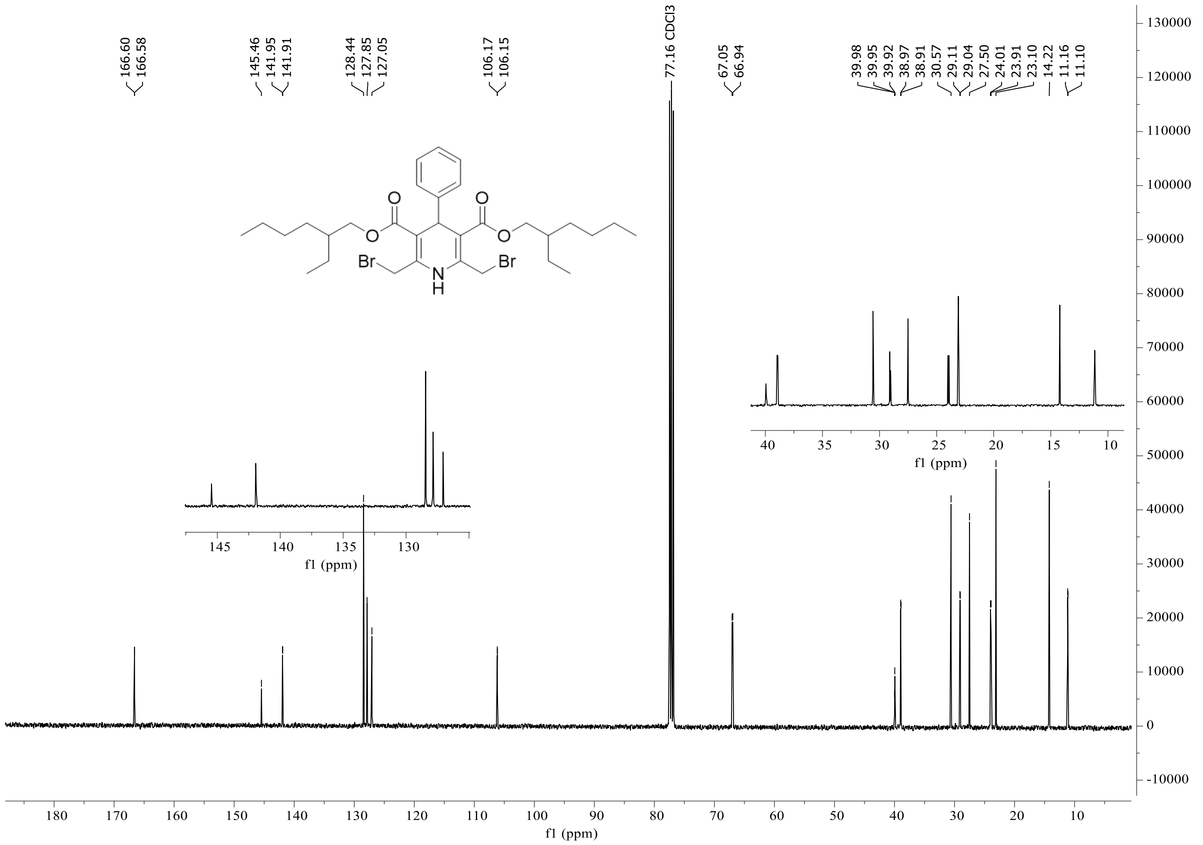

**Figure S10.** $^{13}C$-NMR spectrum of bis(2-ethylhexyl) 2,6-bis(bromomethyl)-4-phenyl-1,4-dihydropyridine-3,5-dicarboxylate (**9a**) in $CDCl_3$.

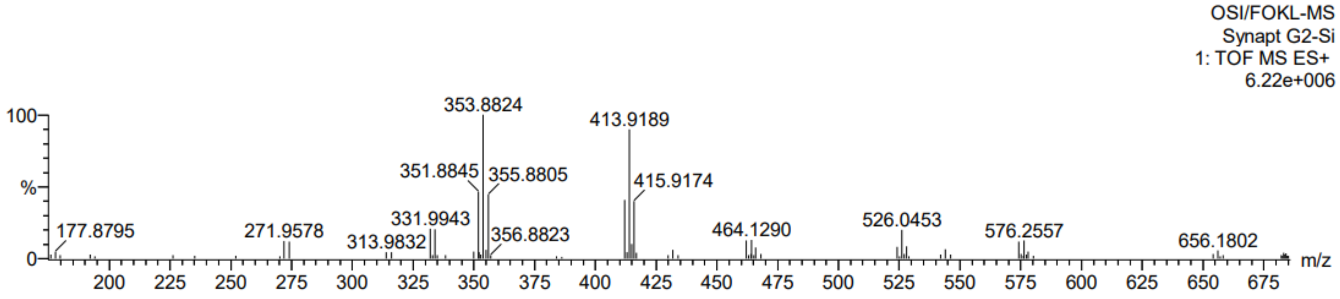


| | Calc. Mass | mDa | PPM | DBE | i-FIT | Norm | Conf(%) | Formula |
|---|---|---|---|---|---|---|---|---|
| Minimum: | | | | -0.5 | | | | |
| Maximum: | | 10.0 | 3.0 | 200.0 | | | | |
| Mass | Calc. Mass | mDa | PPM | DBE | i-FIT | Norm | Conf(%) | Formula |
| 654.1784 | 654.1794 | -1.0 | -1.5 | 8.5 | 60.2 | 0.187 | 82.96 | C31 H46 N O4 Br2 |
| | 654.1767 | 1.7 | 2.6 | 9.5 | 61.8 | 1.770 | 17.04 | C27 H42 N7 O2 Br2 |

**Figure S11.** HRMS data of bis(2-ethylhexyl) 2,6-bis(bromomethyl)-4-phenyl-1,4-dihydropyridine-3,5-dicarboxylate (**9a**).

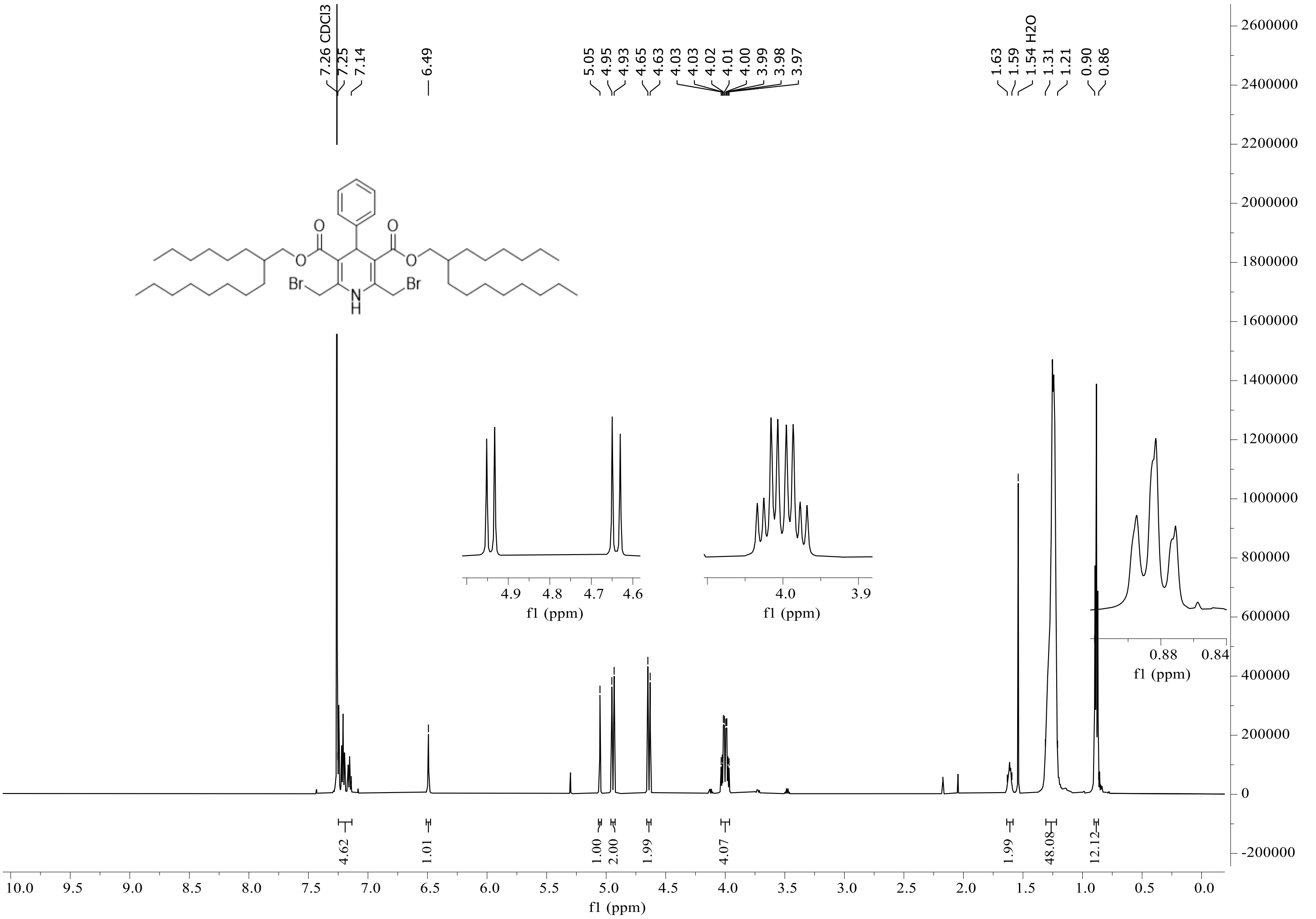


**Figure S12.** $^{1}$H-NMR spectrum of bis(2-hexyldecyl) 2,6-bis(bromomethyl)-4-phenyl-1,4-dihydropyridine-3,5-dicarboxylate (**9b**) in $CDCl_3$.

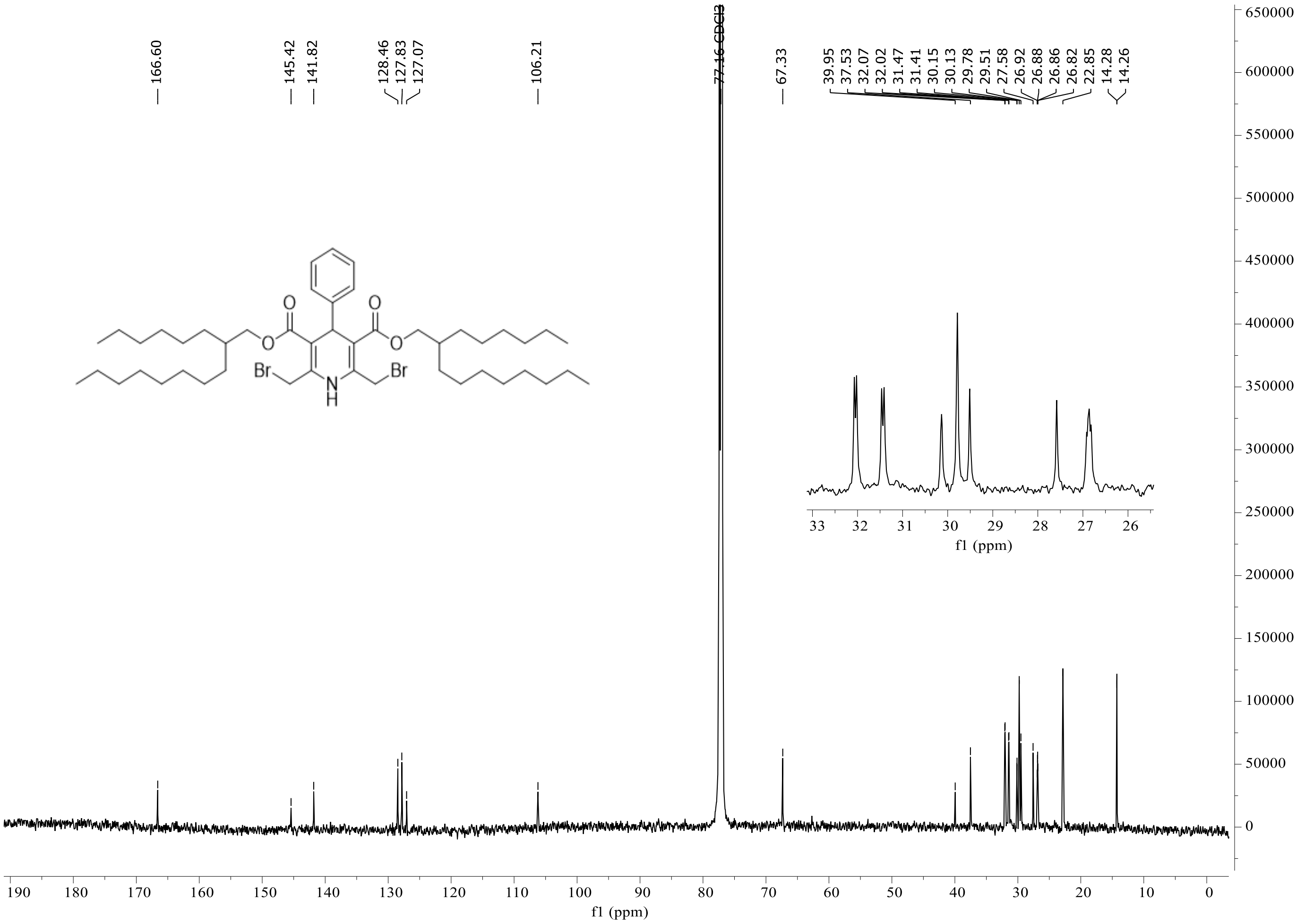

**Figure S13.** $^{13}$C-NMR spectrum of bis(2-hexyldecyl) 2,6-bis(bromomethyl)-4-phenyl-1,4-dihydropyridine-3,5-dicarboxylate (**9b**) in $CDCl_3$.

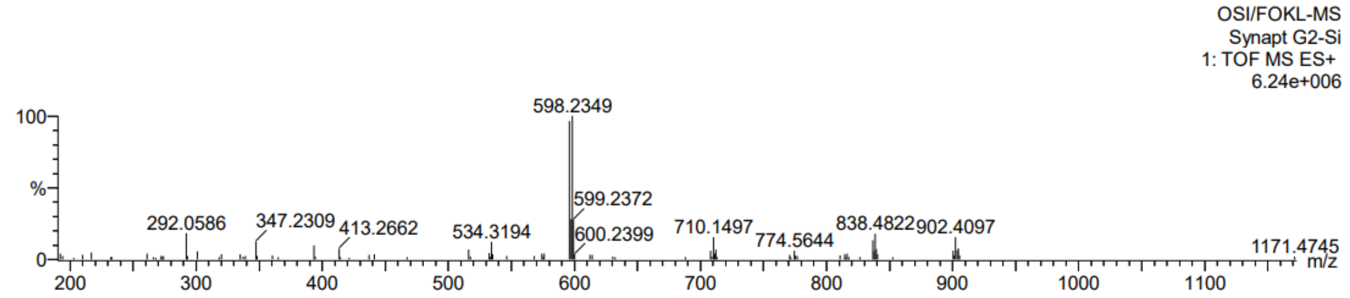

```
Minimum:                              -0.5
Maximum:                10.0    3.0   200.0

Mass      Calc. Mass   mDa     PPM    DBE    i-FIT   Norm    Conf(%)  Formula

900.4106  900.4117     -1.1    -1.2   8.5    44.7    0.342   71.00    C47 H77 N O4 Na Br2
          900.4090     1.6     1.8    9.5    45.9    1.528   21.69    C43 H73 N7 O2 Na Br2
          900.4130     -2.4    -2.7   13.5   47.0    2.617   7.30     C48 H73 N5 Na Br2
```

**Figure S14.** HRMS data of bis(2-hexyldecyl) 2,6-bis(bromomethyl)-4-phenyl-1,4-dihydropyridine-3,5-dicarboxylate (**9b**).

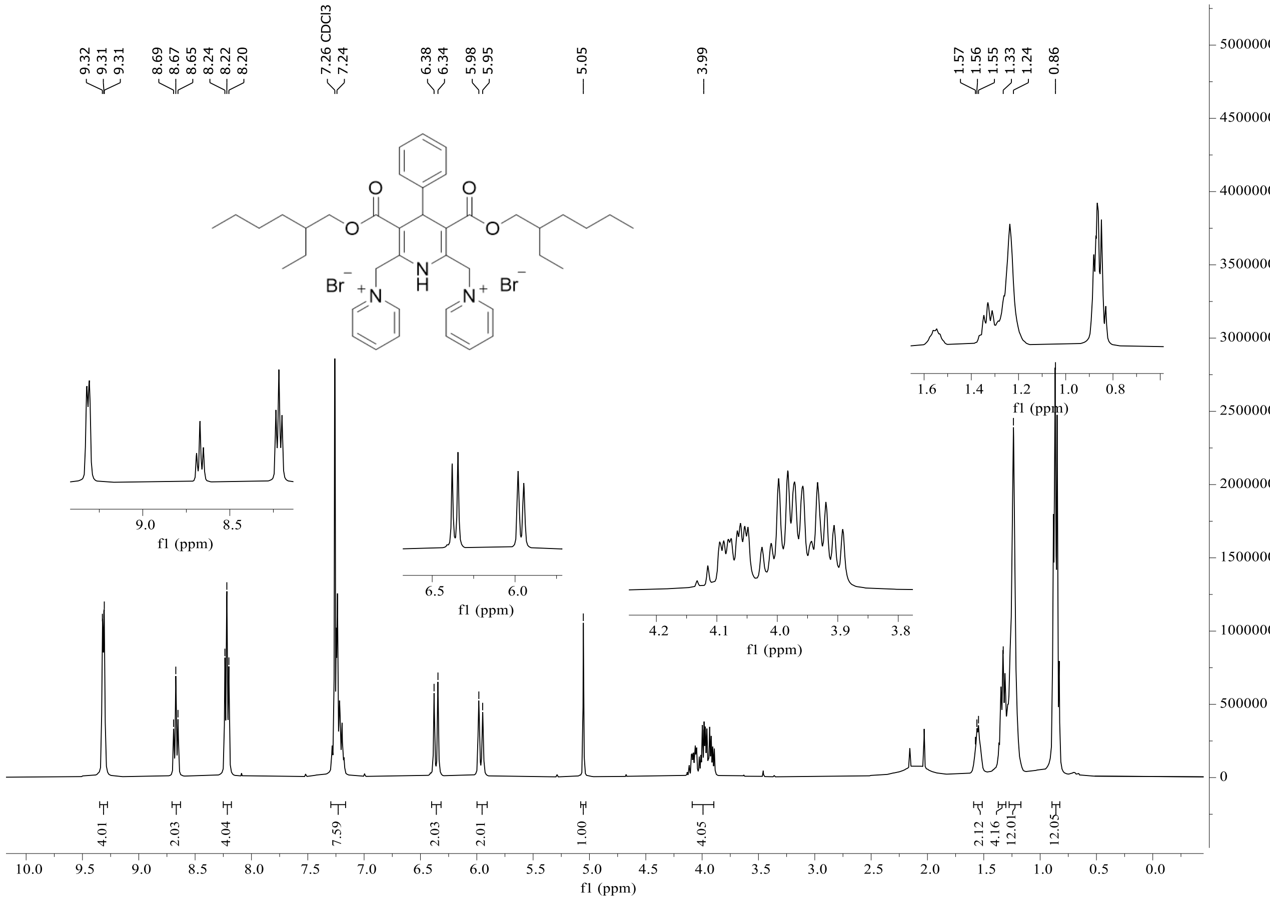


**Figure S15.** $^{1}$H-NMR spectrum of 1,1'-((3,5-bis(((2-ethylhexyl)oxy)carbonyl)-4-phenyl-1,4-dihydropyridine-2,6-diyl)bis(methylene))bis(pyridin-1-ium) dibromide (**14a**) in $CDCl_3$.

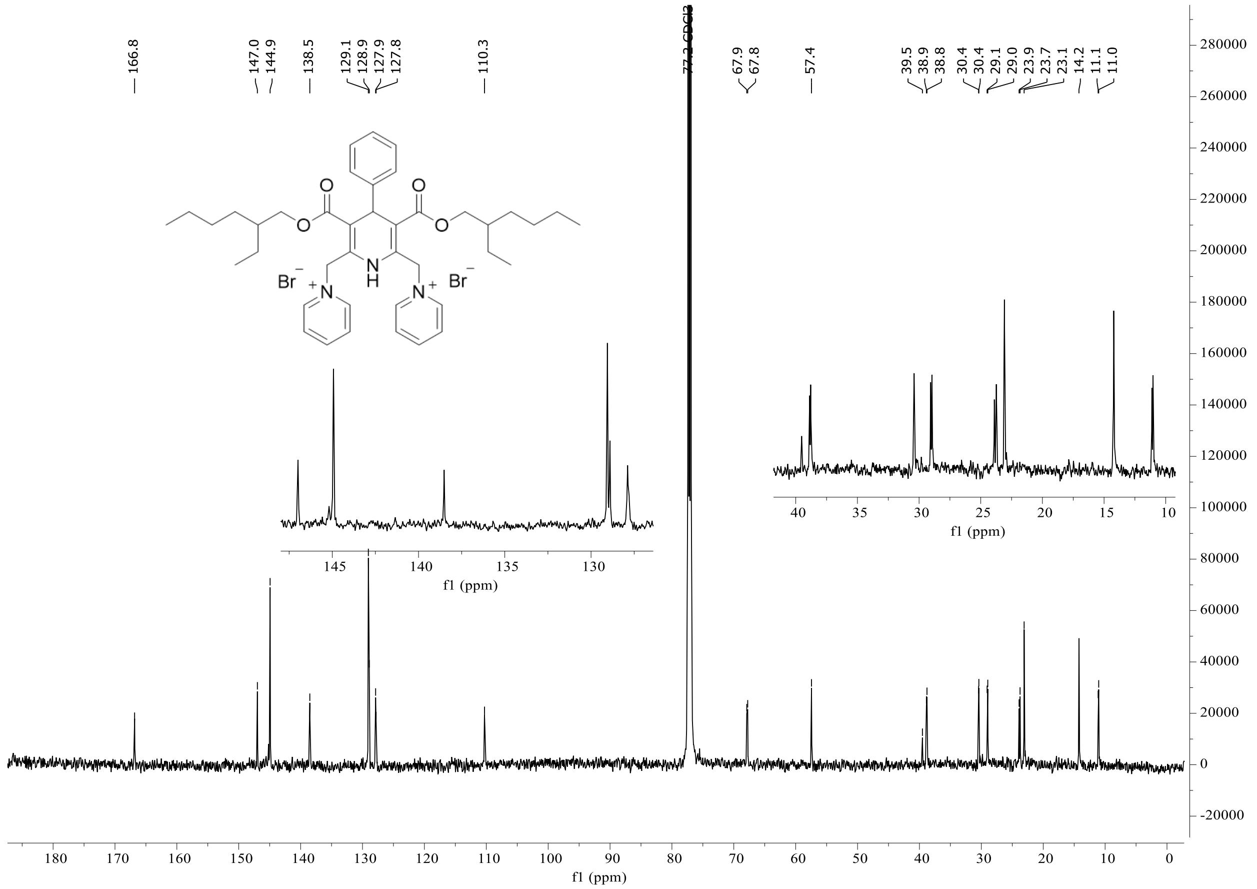


**Figure S16.** $^{13}$C-NMR spectrum of 1,1'-((3,5-bis(((2-ethylhexyl)oxy)carbonyl)-4-phenyl-1,4-dihydropyridine-2,6-diyl)bis(methylene))bis(pyridin-1-ium) dibromide (**14a**) in $CDCl_3$.

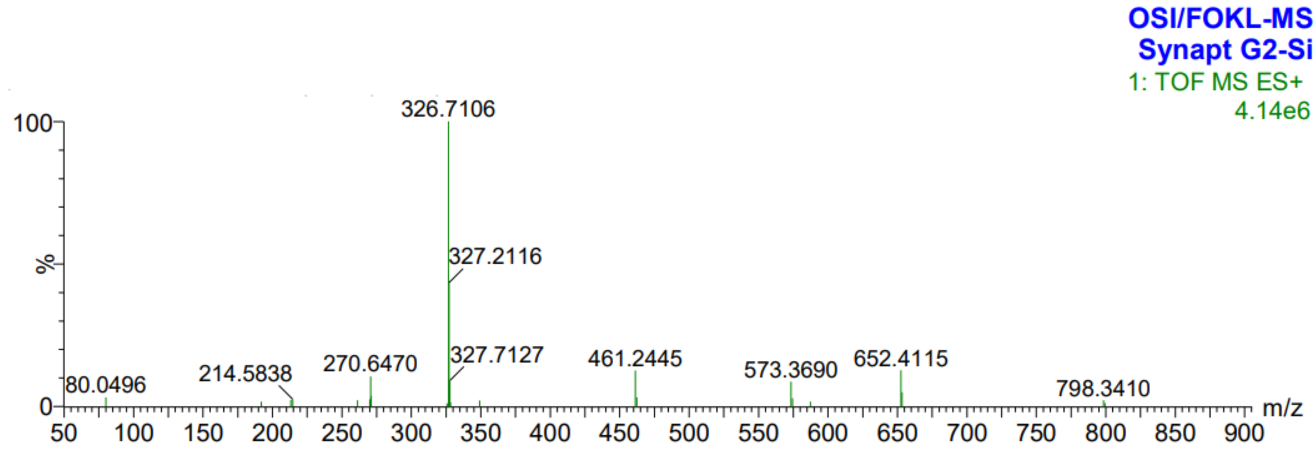


| | |
|---|---|
| Mass $[M]^{2+}$: | 326.7106 |
| Calculated mass $[M]^{2+}$: | 326.7091 |
| Δ mDa: | 1.5 |

**Figure S17.** HRMS data of 1,1'-((3,5-bis(((2-ethylhexyl)oxy)carbonyl)-4-phenyl-1,4-dihydropyridine-2,6-diyl)bis(methylene))bis(pyridin-1-ium) dibromide (**14a**).

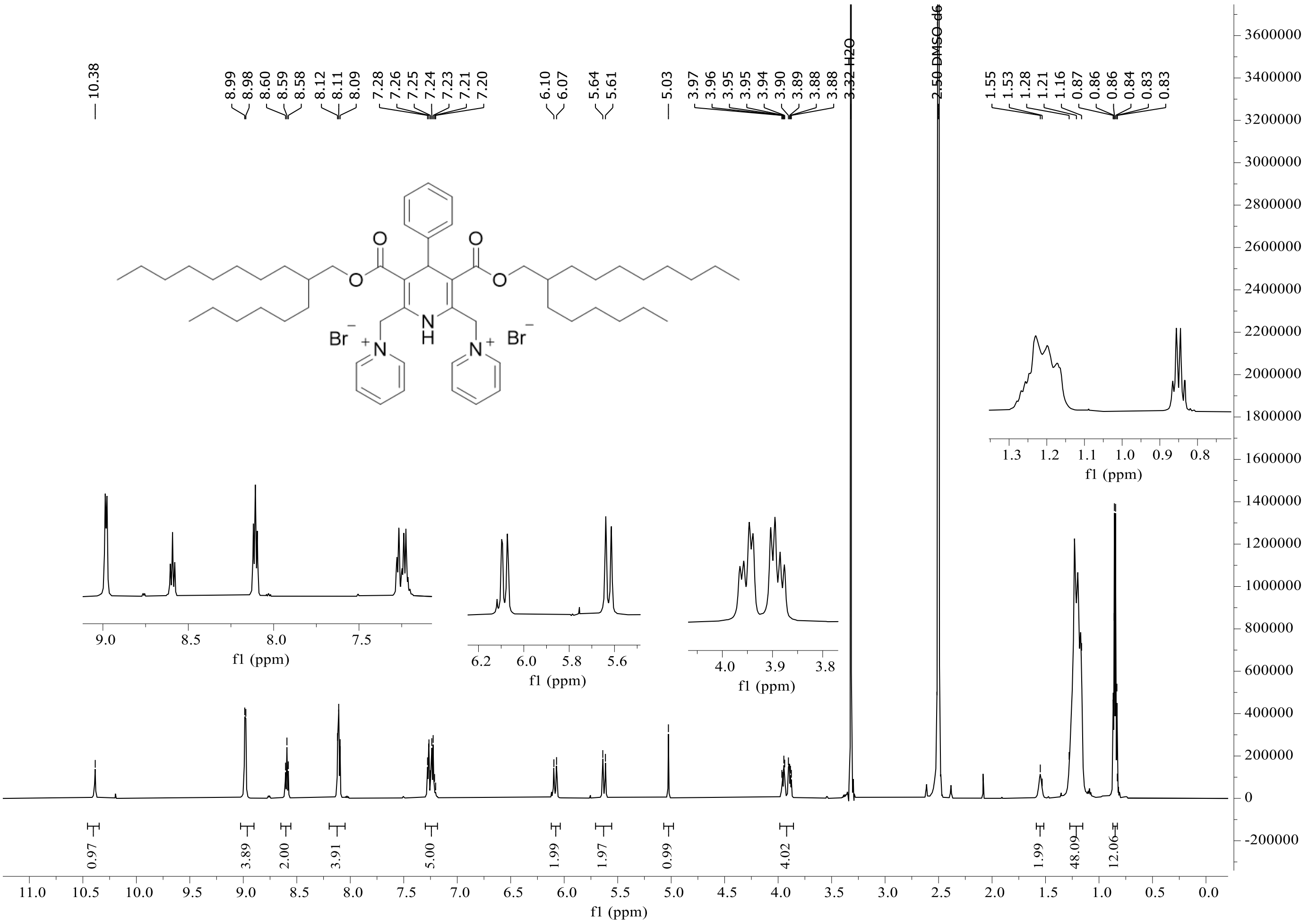


**Figure S18.** $^1$H-NMR spectrum of 1,1'-((3,5-bis(((2-hexyldecyl)oxy)carbonyl)-4-phenyl-1,4-dihydropyridine-2,6-diyl)bis(methylene))bis(pyridin-1-ium) dibromide (**14b**) in DMSO-d$_6$.

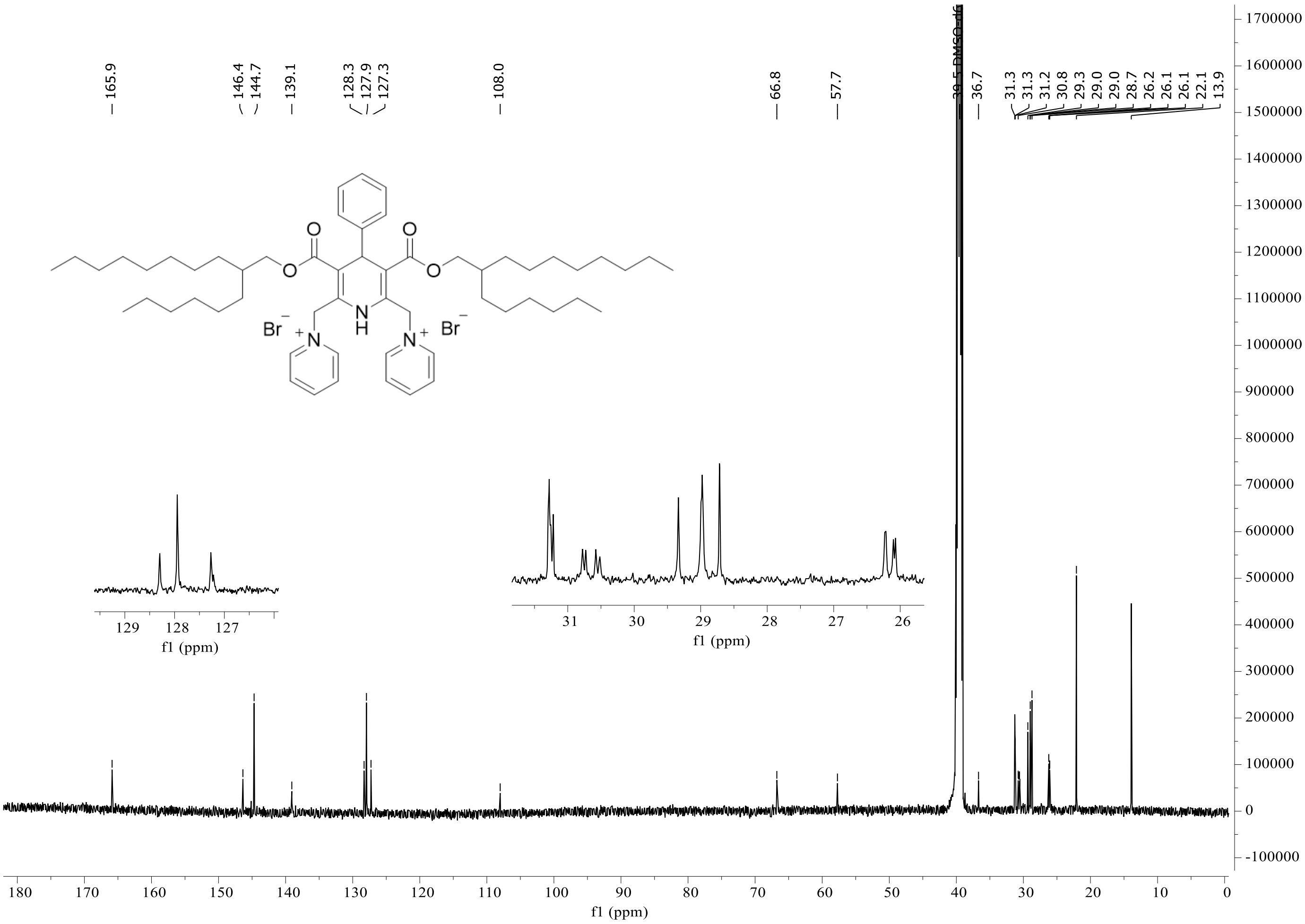

**Figure S19.** $^{13}$C-NMR spectrum of of 1,1'-((3,5-bis(((2-hexyldecyl)oxy)carbonyl)-4-phenyl-1,4-dihydropyridine-2,6-diyl)bis(methylene))bis(pyridin-1-ium) dibromide (**14b**) in DMSO-d₆.

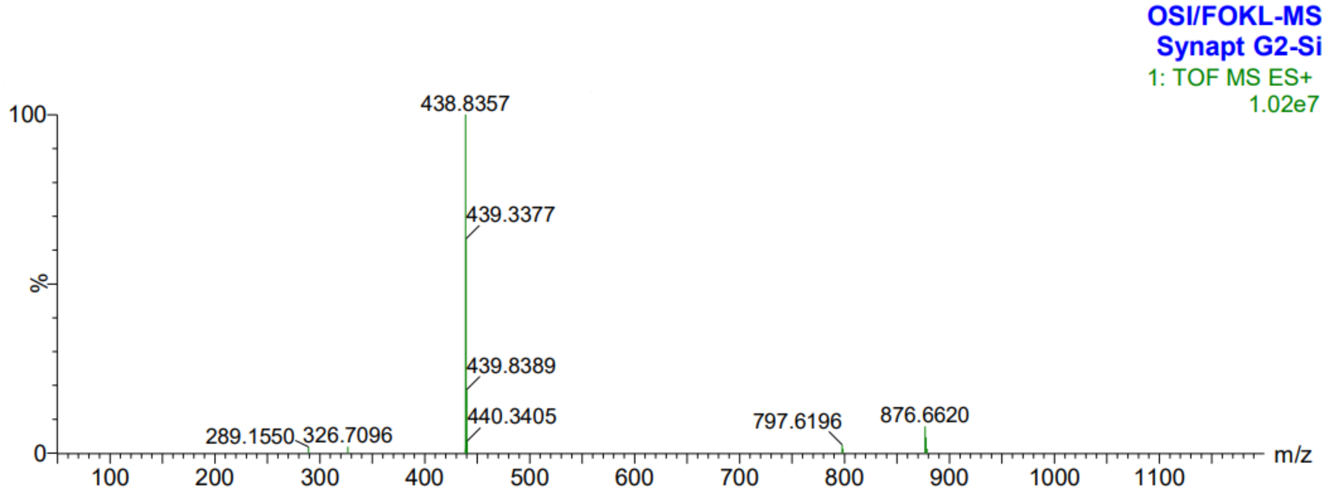


Mass $[M]^{2+}$: 438.8357

Calculated mass $[M]^{2+}$: 438.8343

Δ mDa: 1.4

**Figure S20.** HRMS data of 1,1'-((3,5-bis(((2-hexyldecyl)oxy)carbonyl)-4-phenyl-1,4-dihydropyridine-2,6-diyl)bis(methylene))bis(pyridin-1-ium) dibromide (**14b**).

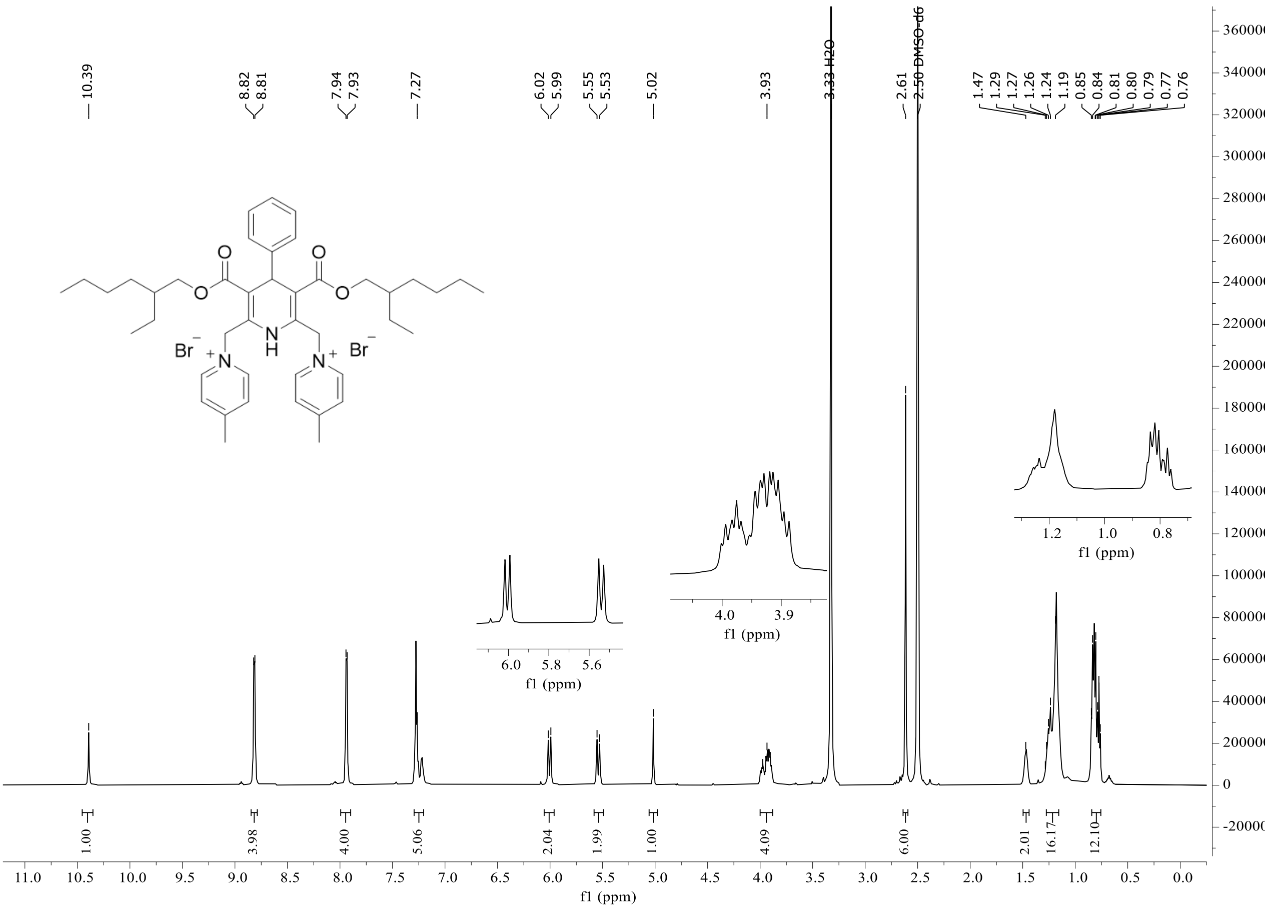

**Figure S21.** $^{1}H$-NMR spectrum of 1,1'-((3,5-bis(((2-ethylhexyl)oxy)carbonyl)-4-phenyl-1,4-dihydropyridine-2,6-diyl)bis(methylene))bis(4-methylpyridin-1-ium) dibromide (**15a**).

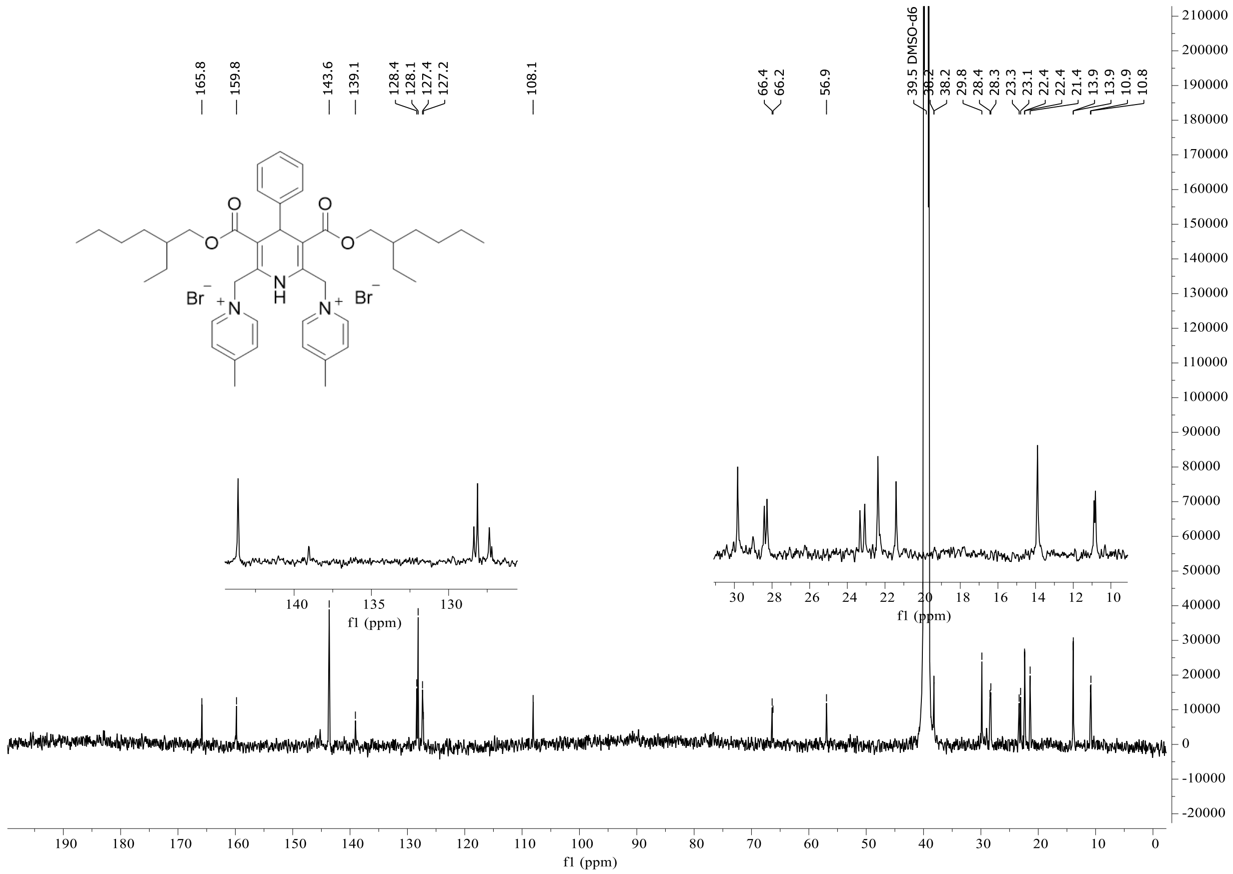

**Figure S22.** $^{13}C$-NMR spectrum of 1,1'-((3,5-bis(((2-ethylhexyl)oxy)carbonyl)-4-phenyl-1,4-dihydropyridine-2,6-diyl)bis(methylene))bis(4-methylpyridin-1-ium) dibromide (**15a**) in DMSO-$d_6$.

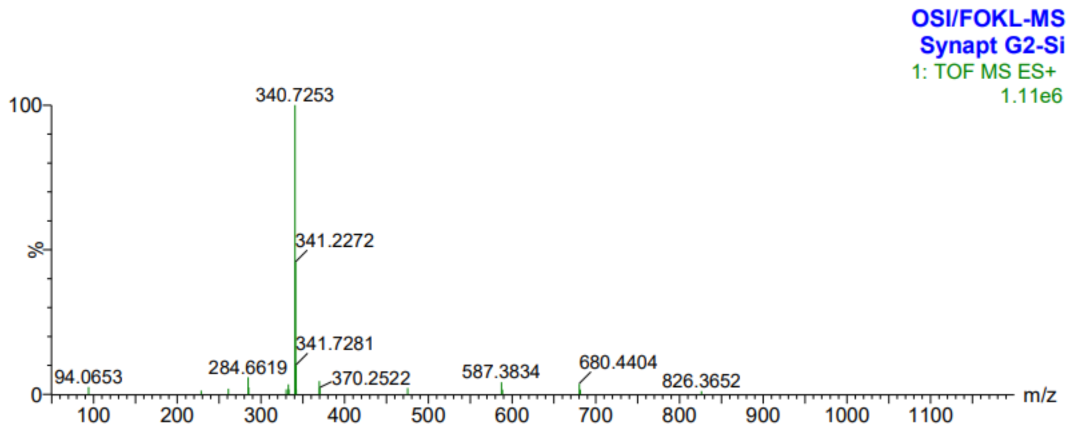


Mass $[M]^{2+}$: 340.7253
Calculated mass $[M]^{2+}$: 340.7247
Δ mDa: 0.6

**Figure S23.** HRMS data of 1,1'-((3,5-bis(((2-ethylhexyl)oxy)carbonyl)-4-phenyl-1,4-dihydropyridine-2,6-diyl)bis(methylene))bis(4-methylpyridin-1-ium) dibromide (**15a**).

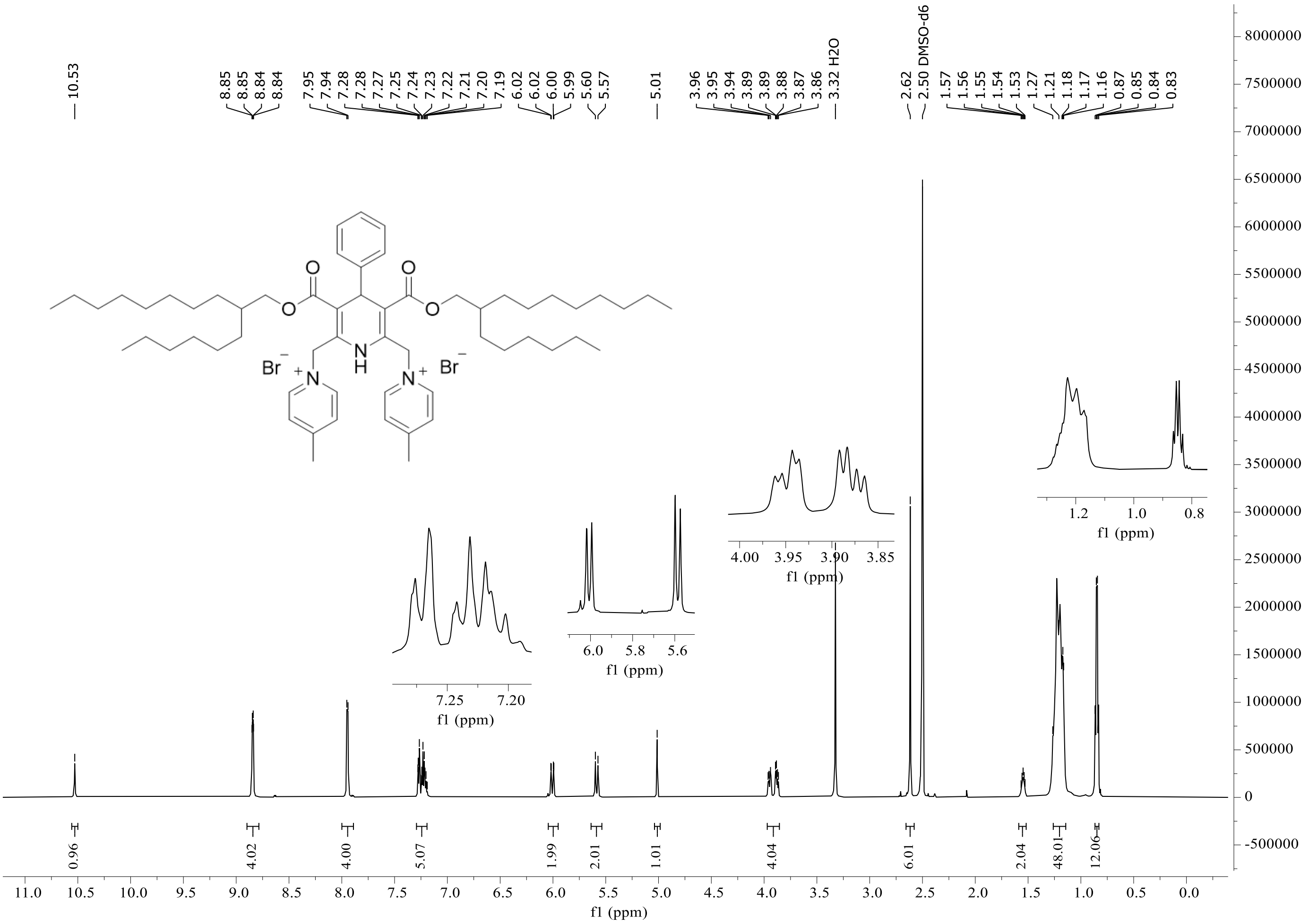


**Figure S24.** ¹H-NMR spectrum of 1,1'-((3,5-bis(((2-hexyldecyl)oxy)carbonyl)-4-phenyl-1,4-dihydro-pyridine-2,6-diyl)bis(methylene))bis(4-methylpyridin-1-ium) dibromide (**15b**) in DMSO-d6.

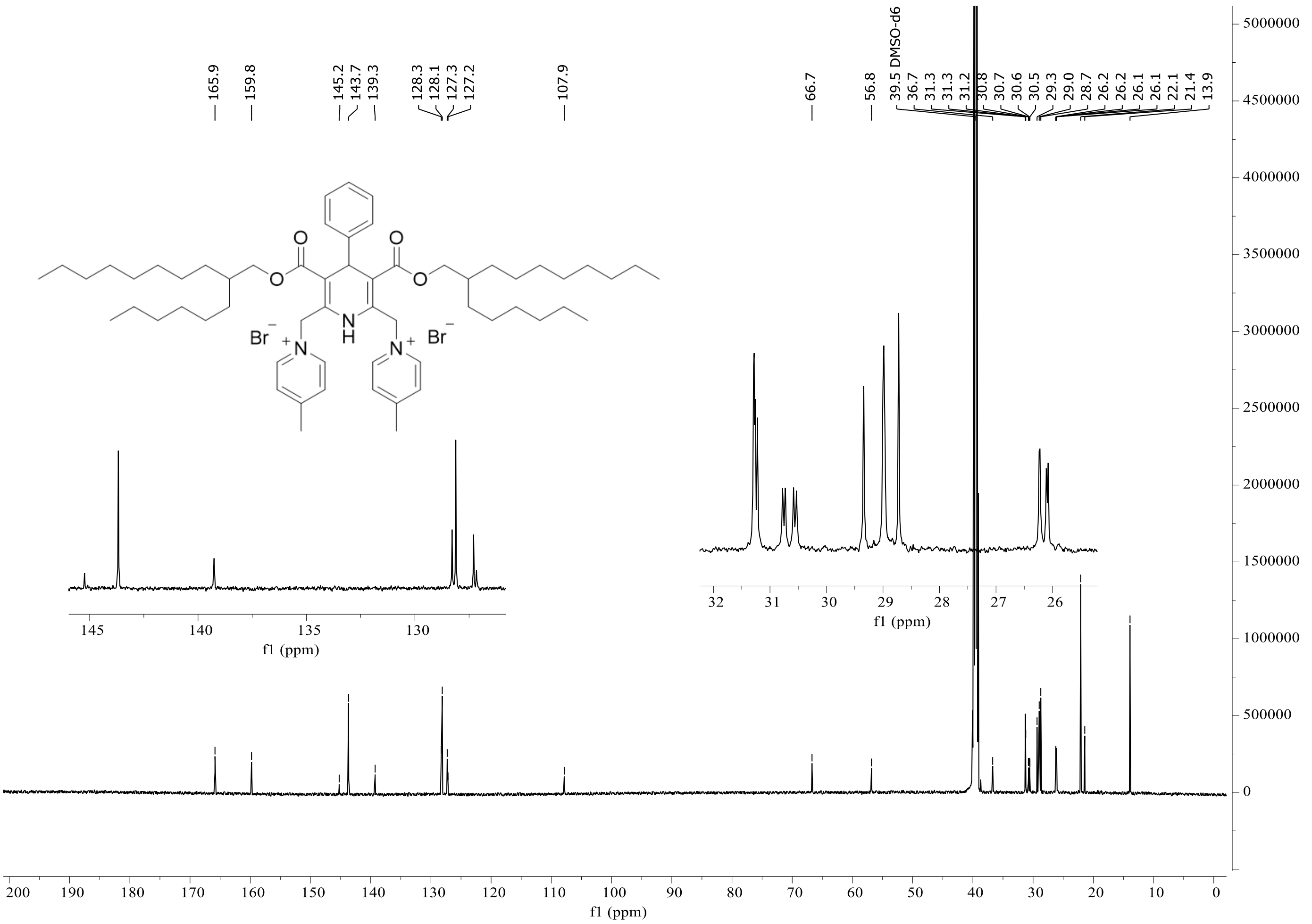


**Figure S25.** $^{13}$C-NMR spectrum of 1,1'-((3,5-bis(((2-hexyldecyl)oxy)carbonyl)-4-phenyl-1,4-dihydropyridine-2,6-diyl)bis(methylene))bis(4-methylpyridin-1-ium) dibromide (**15b**) in DMSO-d$_6$.

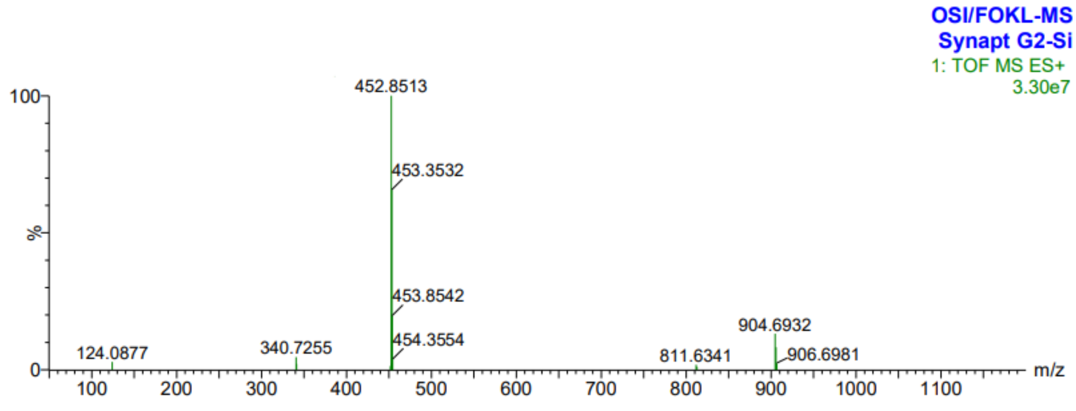


Mass $[M]^{2+}$: 452.8513
Calculated mass $[M]^{2+}$: 452.8499
Δ mDa: 1.4

**Figure S26.** HRMS data of 1,1'-((3,5-bis(((2-hexyldecyl)oxy)carbonyl)-4-phenyl-1,4-dihydropyridine-2,6-diyl)bis(methylene))bis(4-methylpyridin-1-ium) dibromide (**15b**).

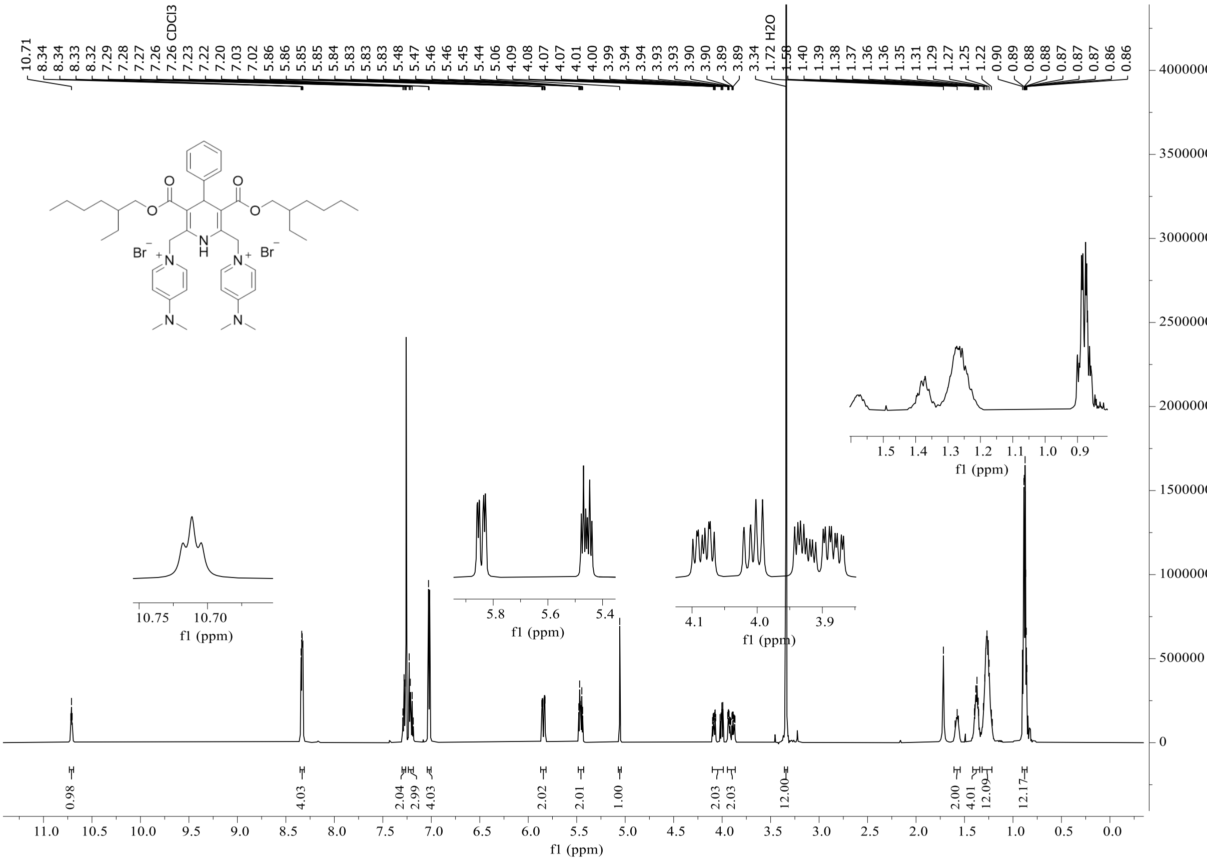

**Figure S27.** $^{1}$H-NMR spectrum of 1,1'-((3,5-bis(((2-ethylhexyl)oxy)carbonyl)-4-phenyl-1,4-dihydro-pyridine-2,6-diyl)bis(methylene))bis(4-(dimethylamino)pyridin-1-ium) dibromide (**16a**) in $CDCl_3$.

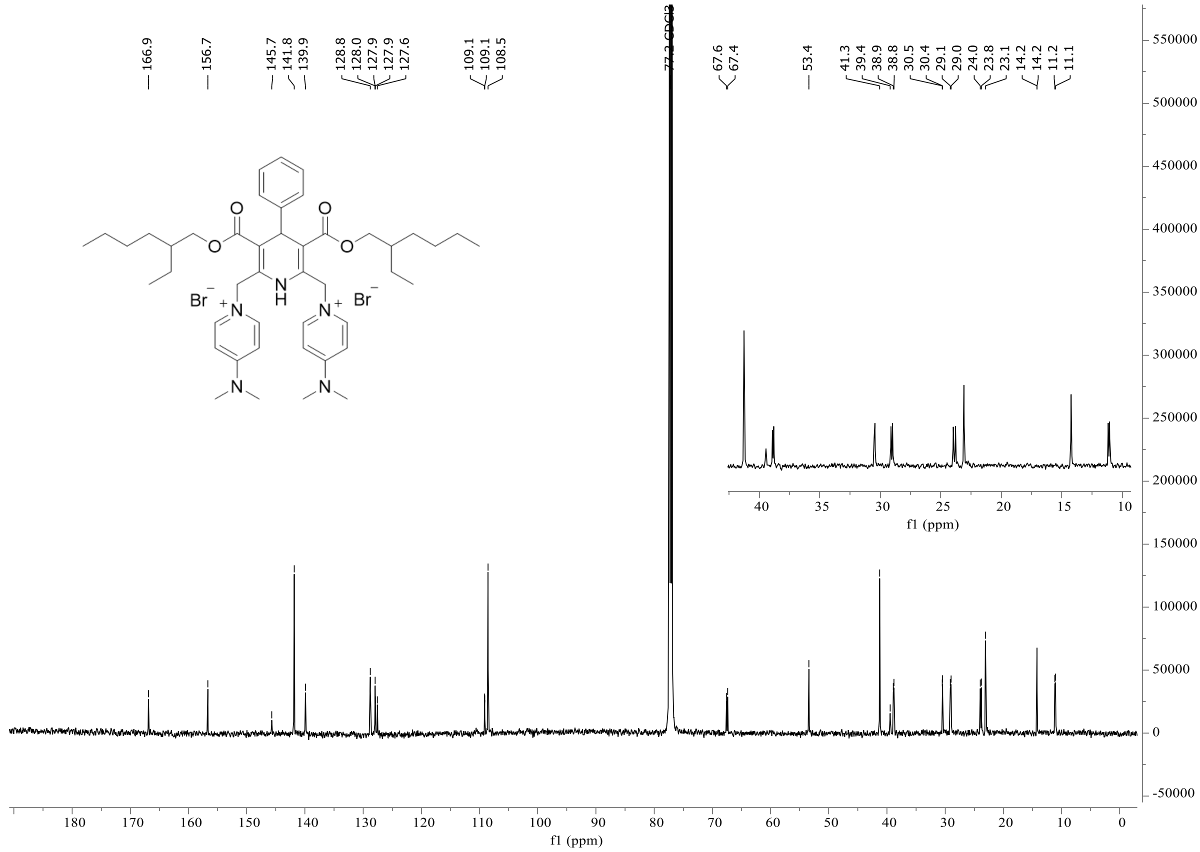

**Figure S28.** $^{13}$C-NMR spectrum of 1,1'-((3,5-bis(((2-ethylhexyl)oxy)carbonyl)-4-phenyl-1,4-dihydro-pyridine-2,6-diyl)bis(methylene))bis(4-(dimethylamino)pyridin-1-ium) dibromide (**16a**) in $CDCl_3$.

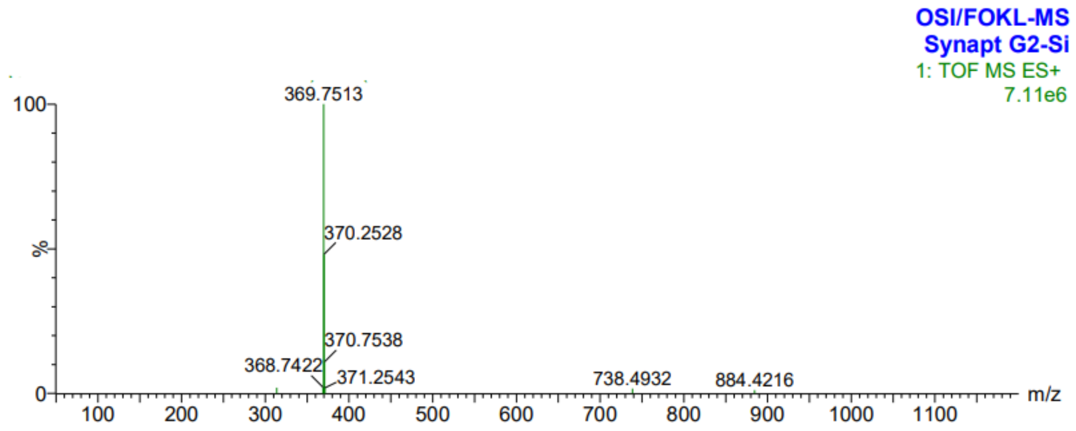


Mass $[M]^{2+}$: 369.7513
Calculated mass $[M]^{2+}$: 369.7513
Δ mDa: 0.0

**Figure S29.** HRMS data of 1,1'-((3,5-bis(((2-ethylhexyl)oxy)carbonyl)-4-phenyl-1,4-dihydropyridine-2,6-diyl)bis(methylene))bis(4-(dimethylamino)pyridin-1-ium) dibromide (**16a**).

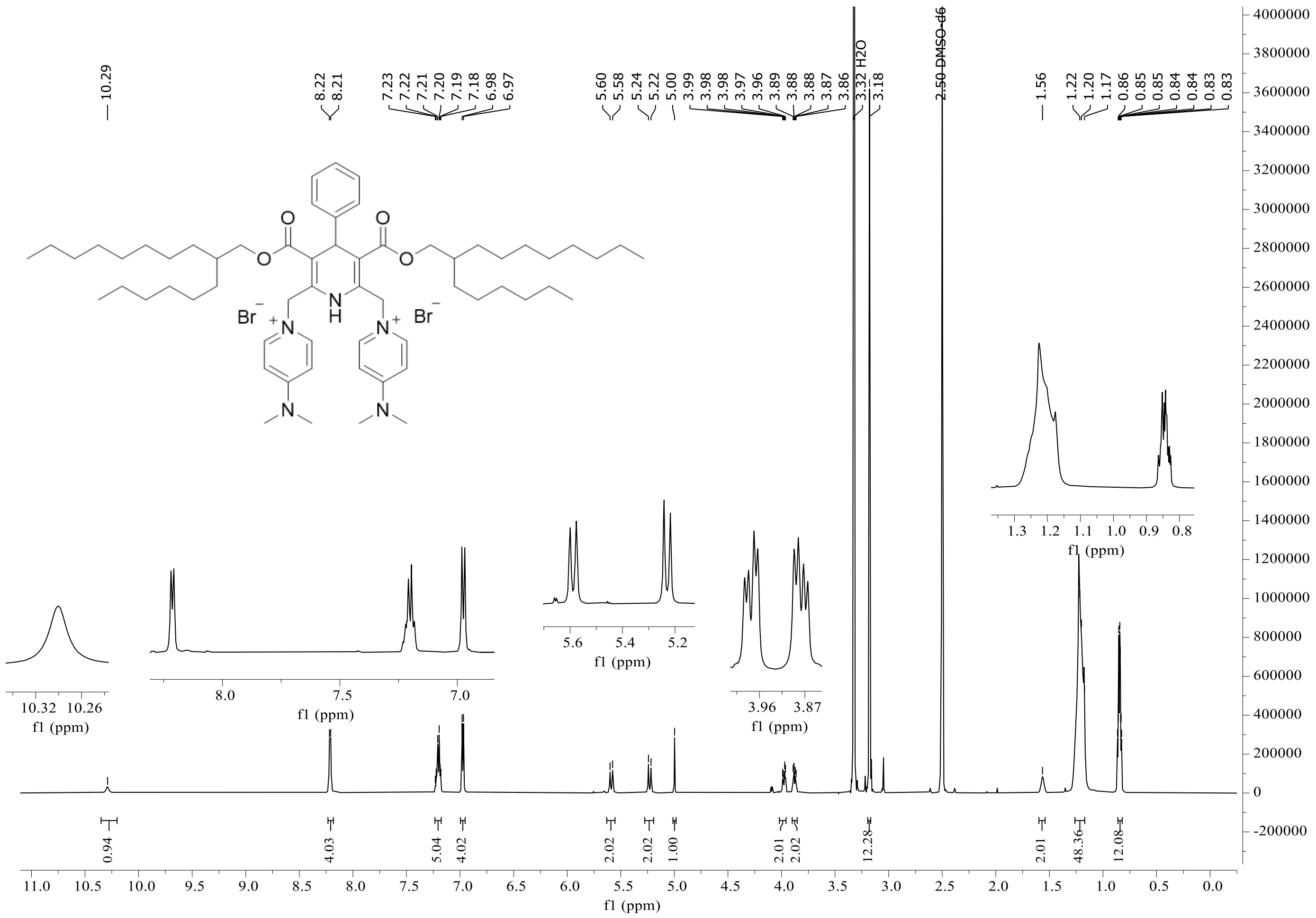


**Figure S30.** $^{1}$H-NMR spectrum of 1,1'-((3,5-bis(((2-hexyldecyl)oxy)carbonyl)-4-phenyl-1,4-dihydropyridine-2,6-diyl)bis(methylene))bis(4-(dimethylamino)pyridin-1-ium) dibromide (**16b**) in DMSO-$d_6$.

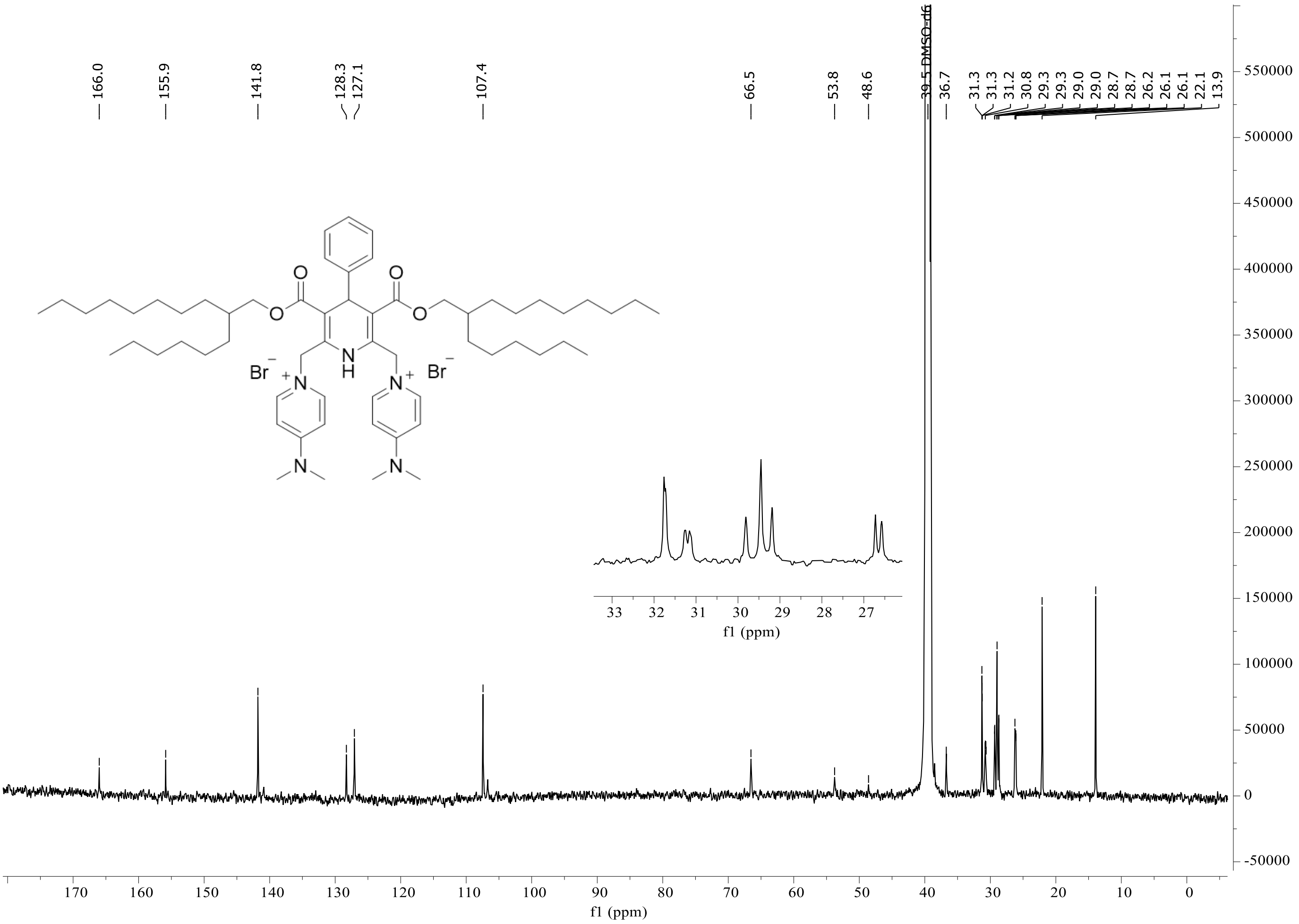


**Figure S31.** $^{13}$C-NMR spectrum of 1,1'-((3,5-bis(((2-hexyldecyl)oxy)carbonyl)-4-phenyl-1,4-dihydropyridine-2,6-diyl)bis(methylene))bis(4-(dimethylamino)pyridin-1-ium) dibromide (**16b**) in DMSO-$d_6$.

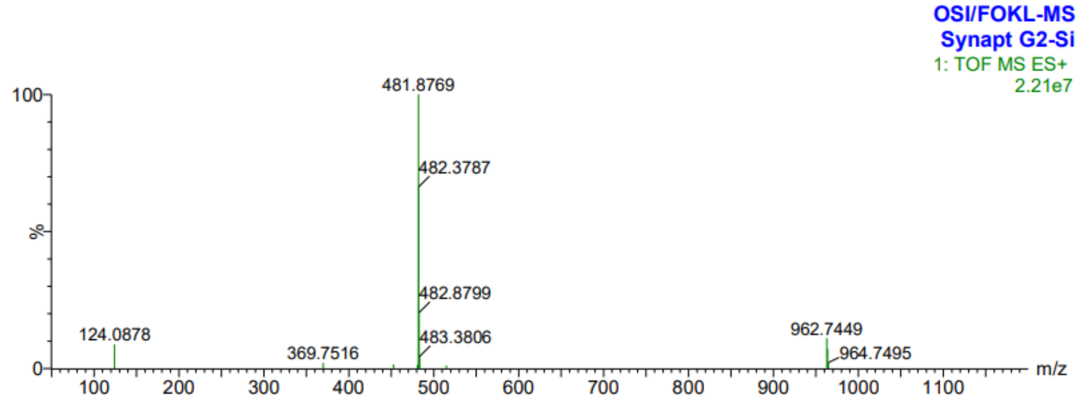


| | |
|---|---|
| Mass [M]$^{2+}$: | 481.8769 |
| Calculated mass [M]$^{2+}$: | 481.8765 |
| Δ mDa: | 0.4 |

**Figure S32.** HRMS data of 1,1'-((3,5-bis(((2-hexyldecyl)oxy)carbonyl)-4-phenyl-1,4-dihydropyridine-2,6-diyl)bis(methylene))bis(4-(dimethylamino)pyridin-1-ium) dibromide (**16b**).

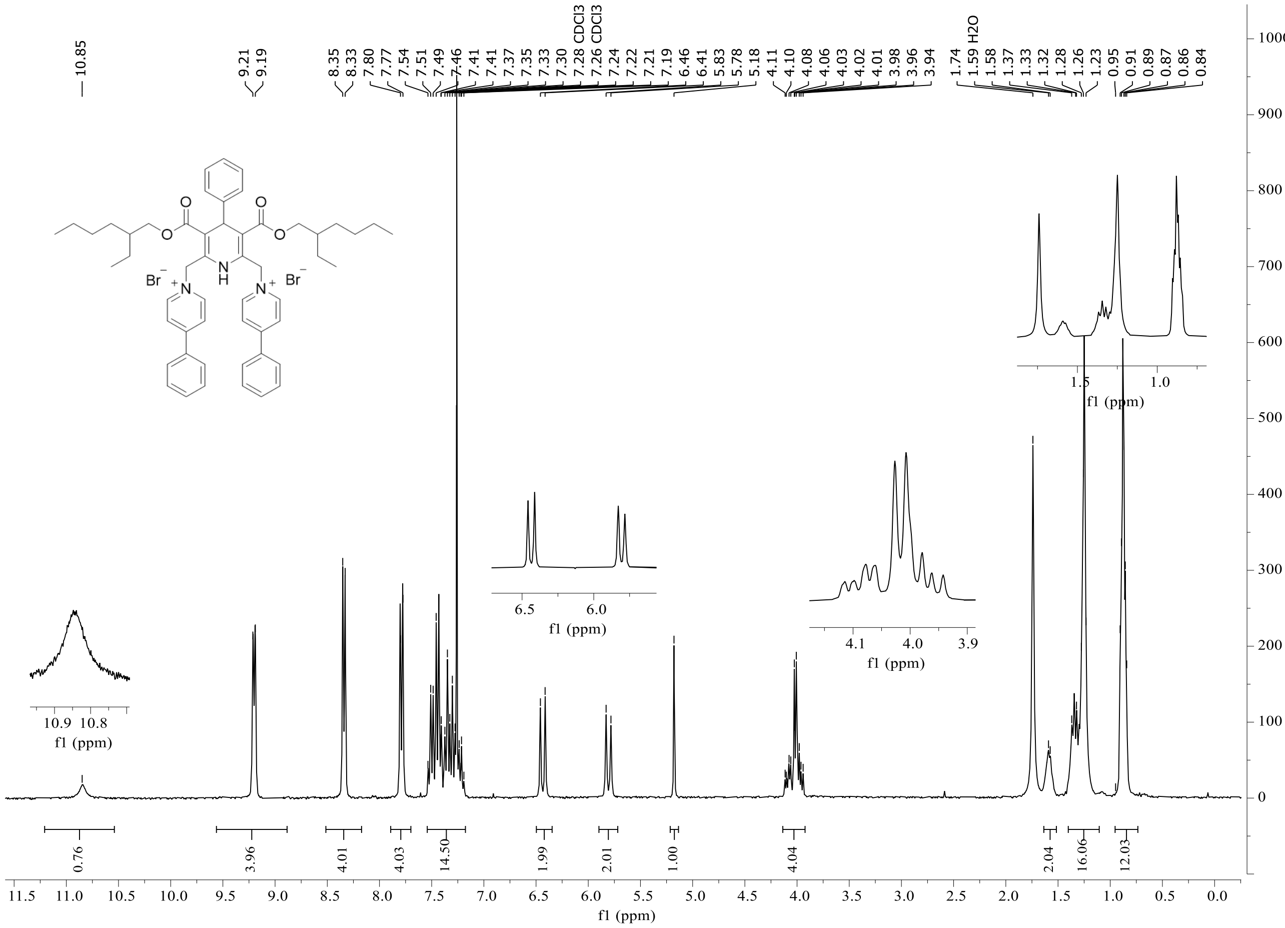

**Figure S33.** $^{1}$H-NMR spectrum of 1,1'-((3,5-bis(((2-ethylhexyl)oxy)carbonyl)-4-phenyl-1,4-dihydropyridine-2,6-diyl)bis(methylene))bis(4-phenylpyridin-1-ium) dibromide (**17a**) in $CDCl_3$.

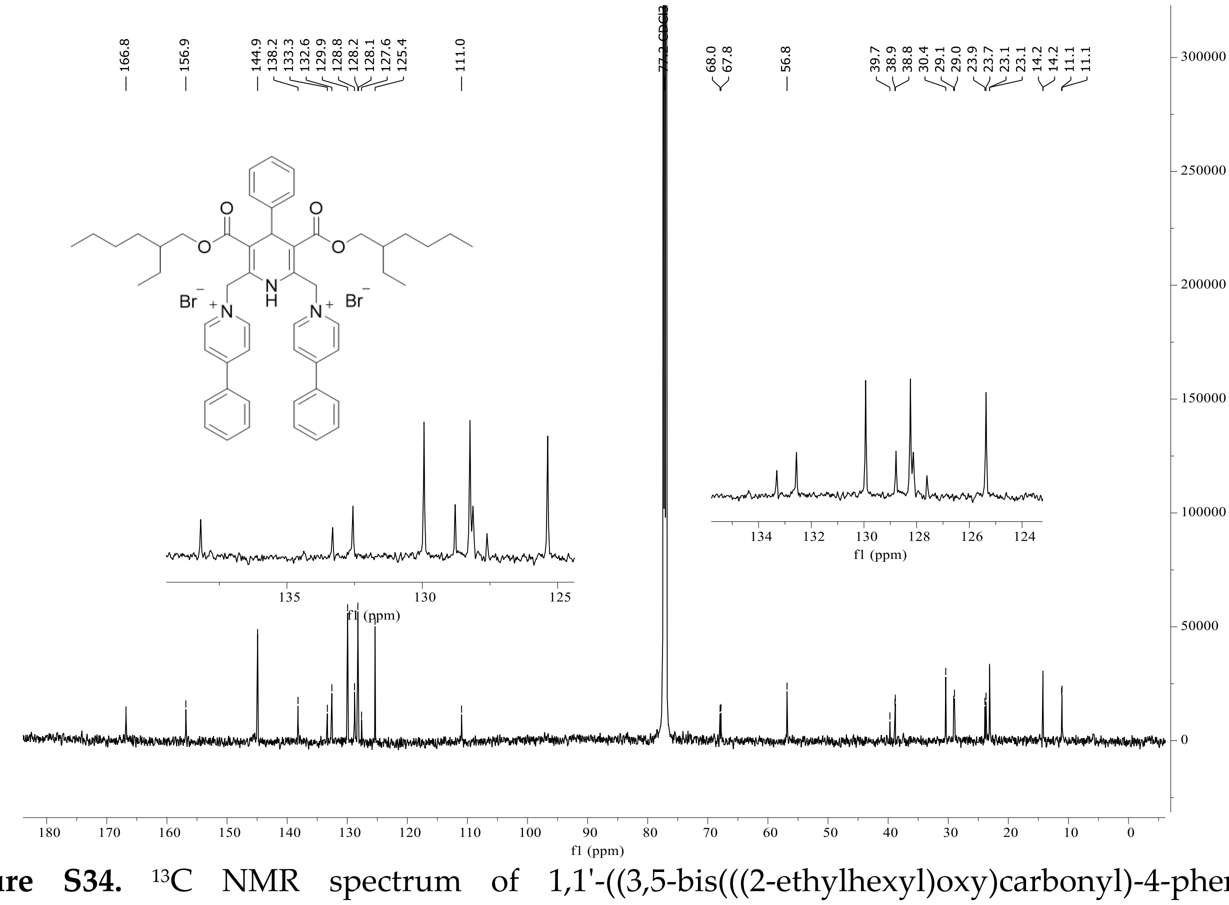

**Figure S34.** $^{13}$C NMR spectrum of 1,1'-((3,5-bis(((2-ethylhexyl)oxy)carbonyl)-4-phenyl-1,4-dihydropyridine-2,6-diyl)bis(methylene))bis(4-phenylpyridin-1-ium) dibromide (**17a**) in $CDCl_3$.

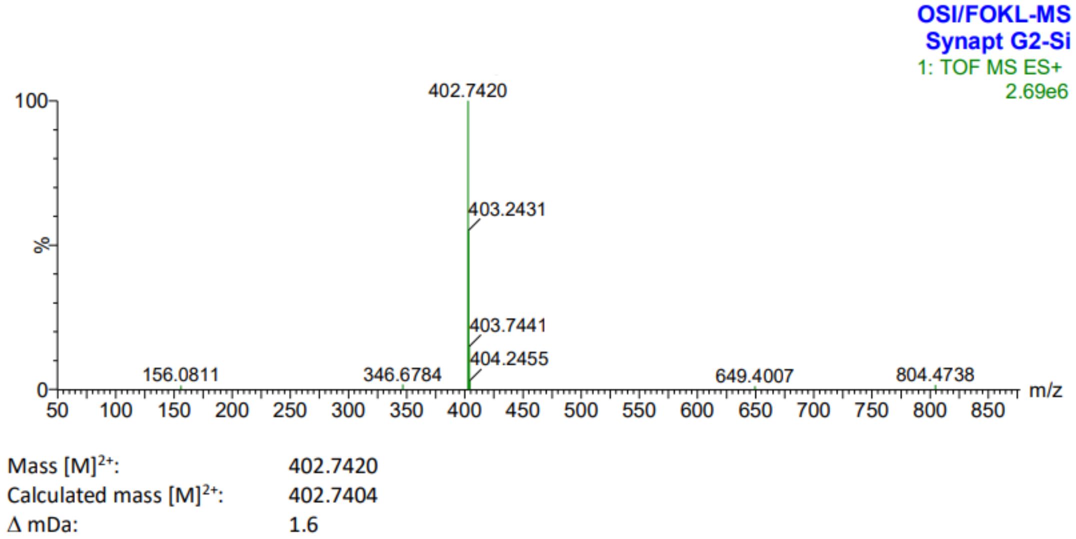


**Figure S35.** HRMS data of 1,1'-((3,5-bis(((2-ethylhexyl)oxy)carbonyl)-4-phenyl-1,4-dihydropyridine-2,6-diyl)bis(methylene))bis(4-phenylpyridin-1-ium) dibromide (**17a**).

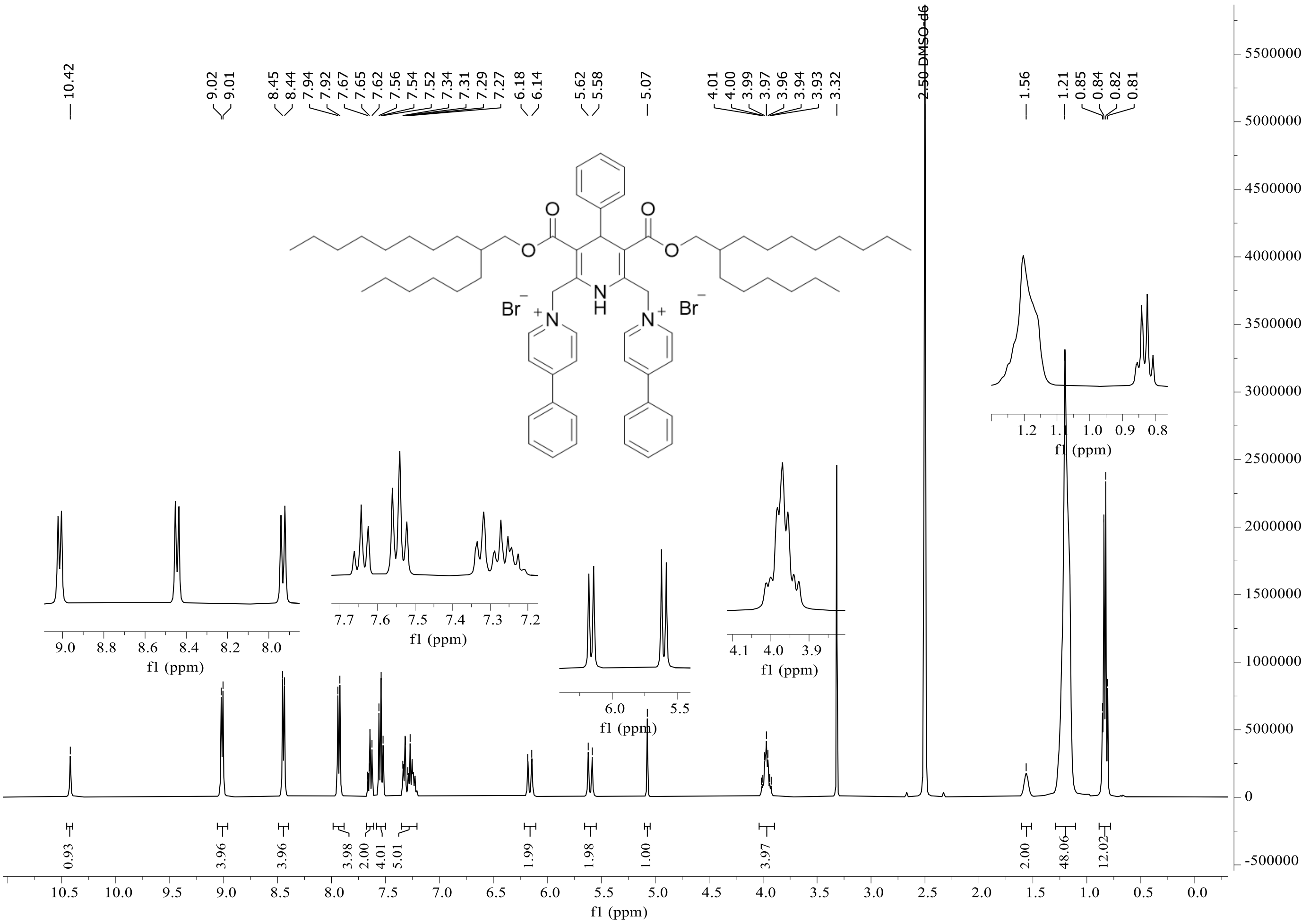


**Figure S36.** $^{1}$H-NMR spectrum of 1,1'-((3,5-bis(((2-hexyldecyl)oxy)carbonyl)-4-phenyl-1,4-dihydropyridine-2,6-diyl)bis(methylene))bis(4-phenylpyridin-1-ium) dibromide (**17b**) in DMSO-$d_6$.

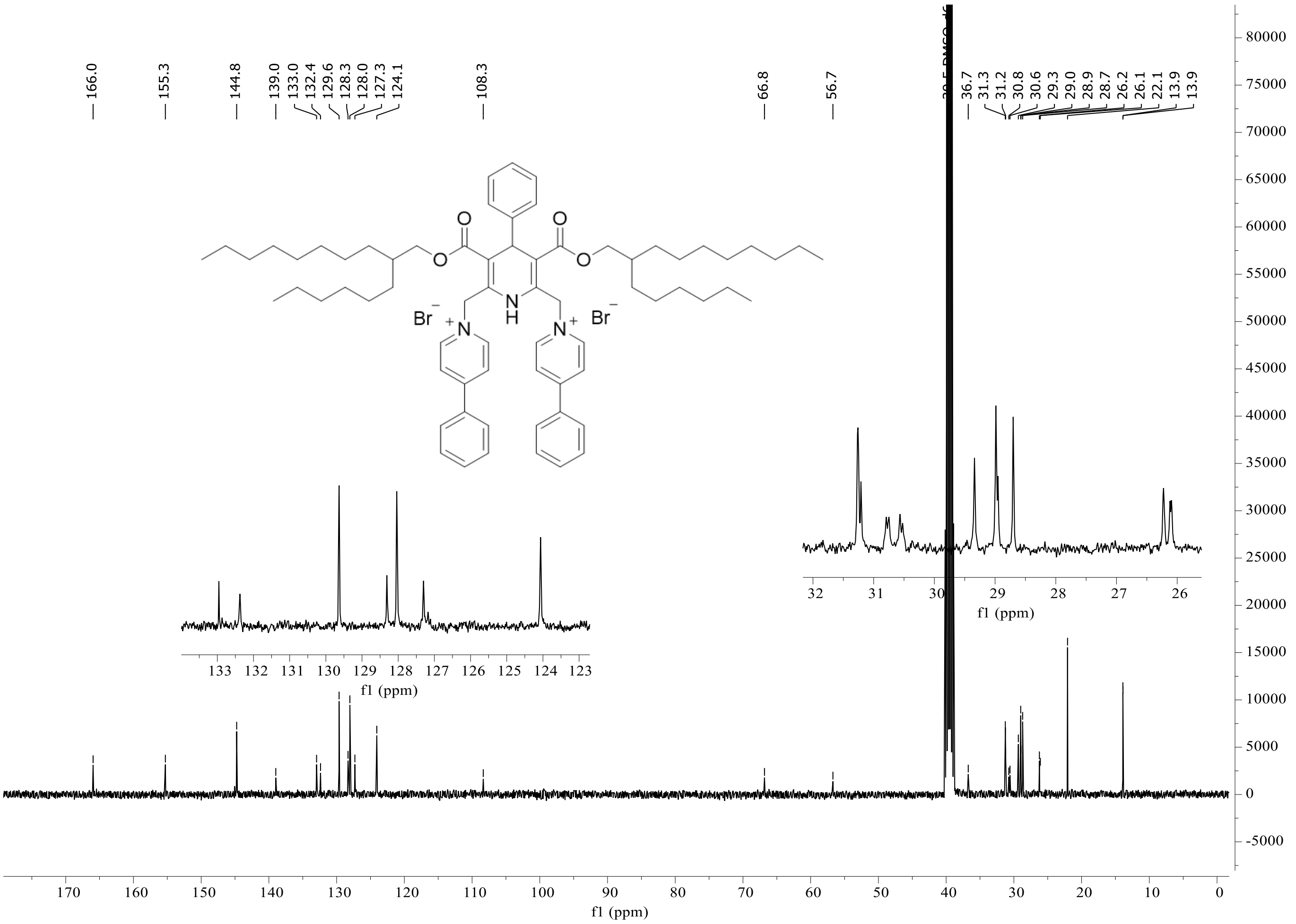

**Figure S37.** $^{13}$C-NMR spectrum of 1,1'-((3,5-bis(((2-hexyldecyl)oxy)carbonyl)-4-phenyl-1,4-dihydro-pyridine-2,6-diyl)bis(methylene))bis(4-phenylpyridin-1-ium) dibromide (**17b**) in DMSO-$d_6$.

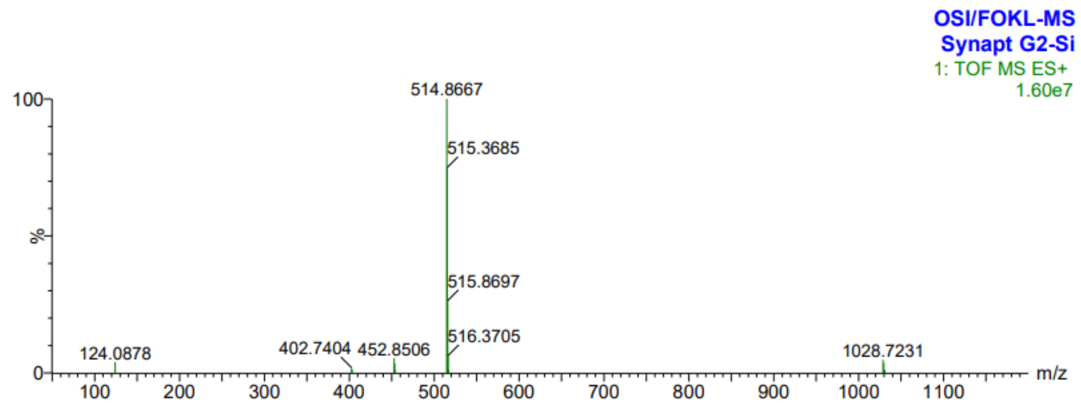


Mass [M]$^{2+}$: 514.8667

Calculated mass [M]$^{2+}$: 514.8659

Δ mDa: 0.8

**Figure S38.** HRMS data of 1,1'-((3,5-bis(((2-hexyldecyl)oxy)carbonyl)-4-phenyl-1,4-dihydropyridine-2,6-diyl)bis(methylene))bis(4-phenylpyridin-1-ium) dibromide (**17b**).

**Table S1.** Values of average diameter ($Z_{av}$) and polydispersity index (PDI) of nanoparticles formed by new 1,4-DHP amphiphiles **1**, **14a**,**b**-**16a**,**b** and **17b** obtained by DLS measurements in water for freshly prepared samples and after storage for 3 and 14 days at r.t., concentration of samples 0.5 mM.

| Entry | Comp. | Freshly prepared | | 3 days | | 14 days | |
|---|---|---|---|---|---|---|---|
| | | $Z_{av}$, nm | PDI | $Z_{av}$, nm | PDI | $Z_{av}$, nm | PDI |
| 1 | **1** | 114 ± 1 | 0.199 ± 0.011 | 130 ± 1 | 0.189 ± 0.010 | 180 ± 1 | 0.231 ± 0.009 |
| 2 | **14a** | 250 ± 10 | 0.397 ± 0.079 | 439 ± 106 | 0.486 ± 0.051 | 308 ± 22 | 0.379 ± 0.047 |
| 3 | **14b** | 52 ± 2 | 0.366 ± 0.028 | 64 ± 1 | 0.194 ± 0.007 | 84 ± 1 | 0.228 ± 0.009 |
| 4 | **15a** | 158 ± 1 | 0.279 ± 0.009 | 157 ± 2 | 0.246 ± 0.023 | 157 ± 4 | 0.259 ± 0.024 |
| 5 | **15b** | 56 ± 4 | 0.527 ± 0.094 | 50 ± 1 | 0.253 ± 0.018 | 67 ± 1 | 0.391 ± 0.017 |
| 6 | **16a** | 337 ± 46 | 0.341 ± 0.046 | 366 ± 38 | 0.346 ± 0.055 | 342 ± 35 | 0.360 ± 0.023 |
| 7 | **16b** | 67 ± 11 | 0.277 ± 0.066 | 59 ± 1 | 0.180 ± 0.007 | 81 ± 1 | 0.197 ± 0.004 |
| 8 | **17b** | 74 ± 1 | 0.194 ± 0.013 | 74 ± 1 | 0.163 ± 0.014 | 78 ± 1 | 0.166 ± 0.011 |

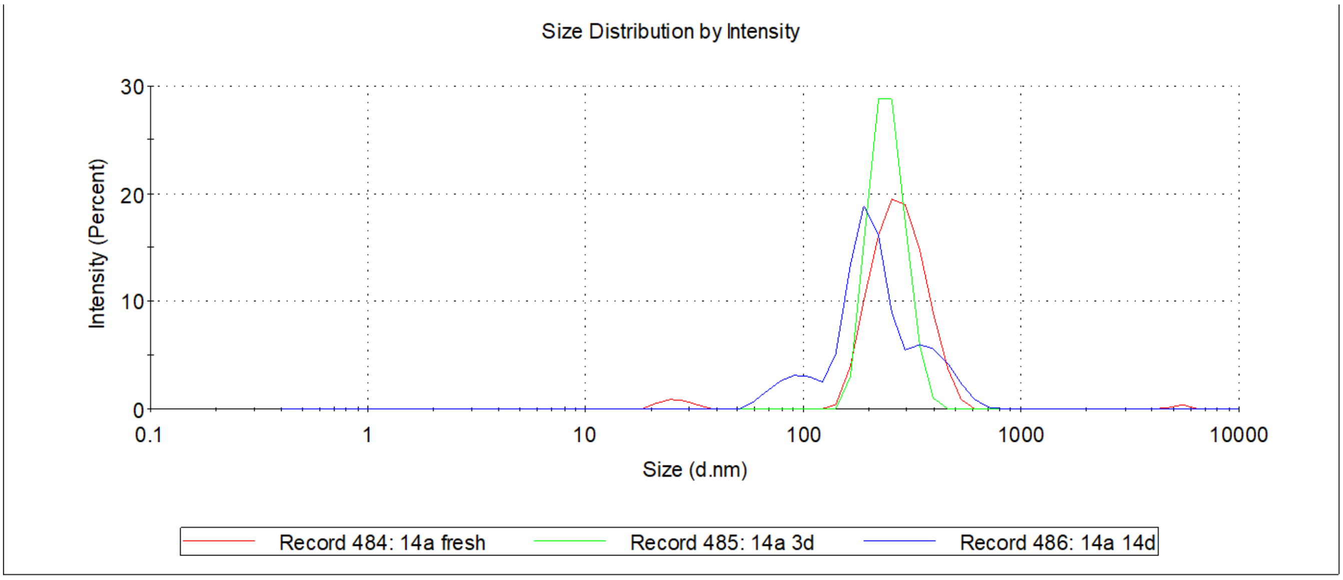


**Figure S39.** DLS size distribution of 1,1'-((3,5-bis(((2-ethylhexyl)oxy)carbonyl)-4-phenyl-1,4-dihydropyridine-2,6-diyl)bis(methylene))bis(pyridin-1-ium) dibromide (**14a**).

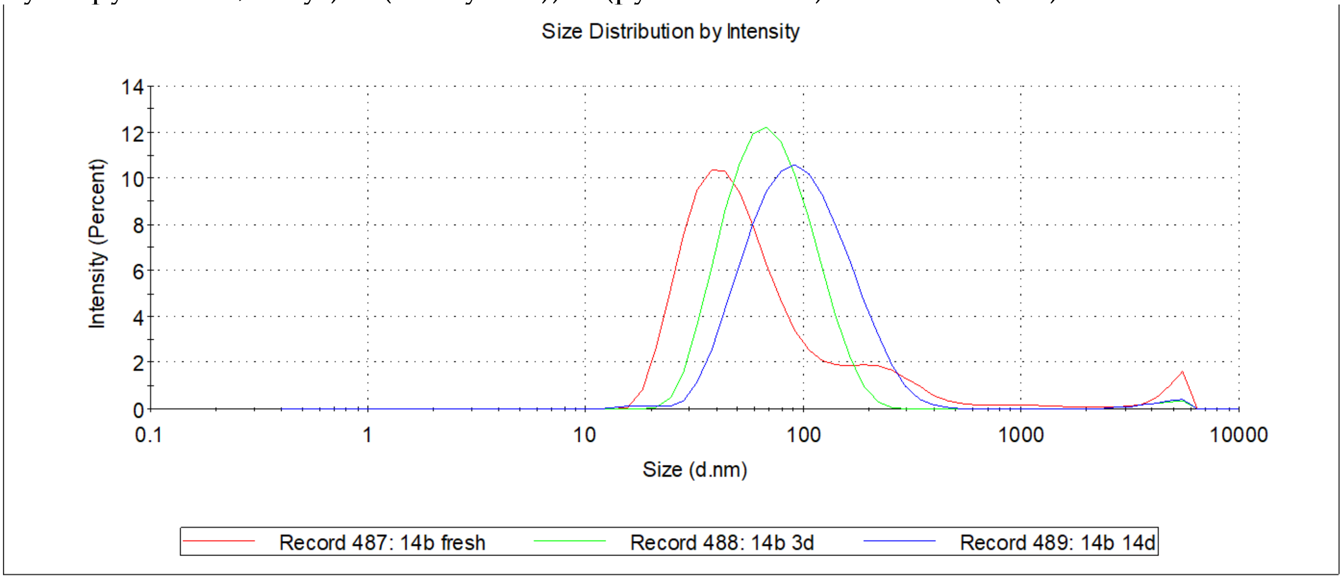


**Figure S40.** DLS size distribution of 1,1'-((3,5-bis(((2-hexyldecyl)oxy)carbonyl)-4-phenyl-1,4-dihydropyridine-2,6-diyl)bis(methylene))bis(pyridin-1-ium) dibromide (**14b**).

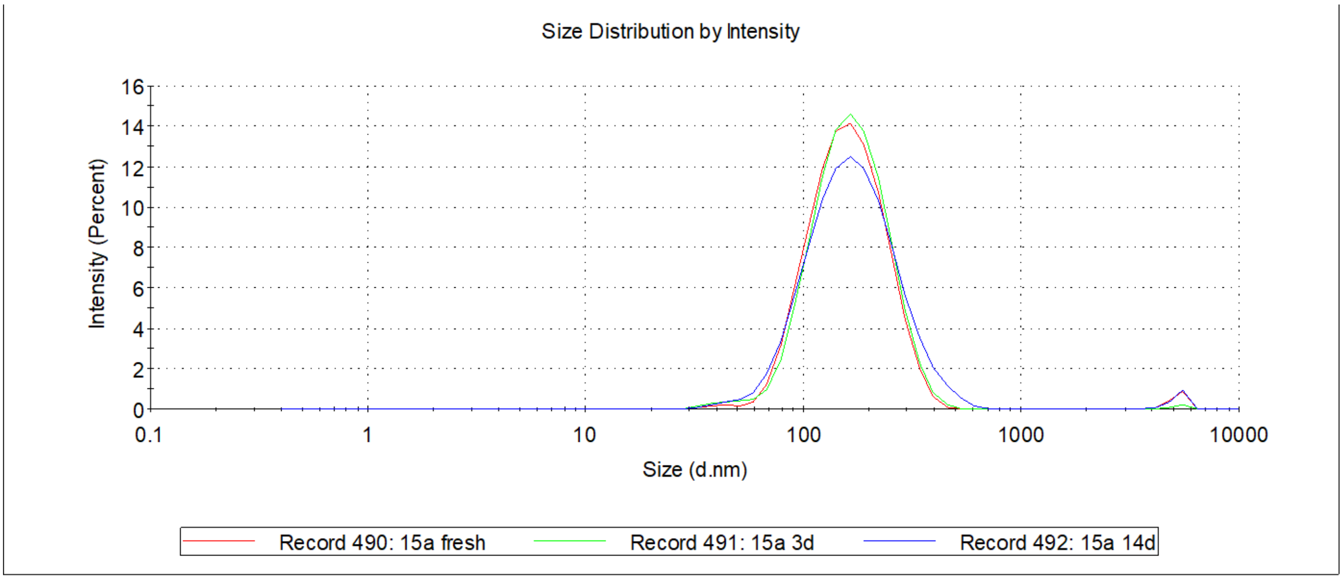


**Figure S41.** DLS size distribution of 1,1'-((3,5-bis(((2-ethylhexyl)oxy)carbonyl)-4-phenyl-1,4-dihydropyridine-2,6-diyl)bis(methylene))bis(4-methylpyridin-1-ium) dibromide (**15a**).

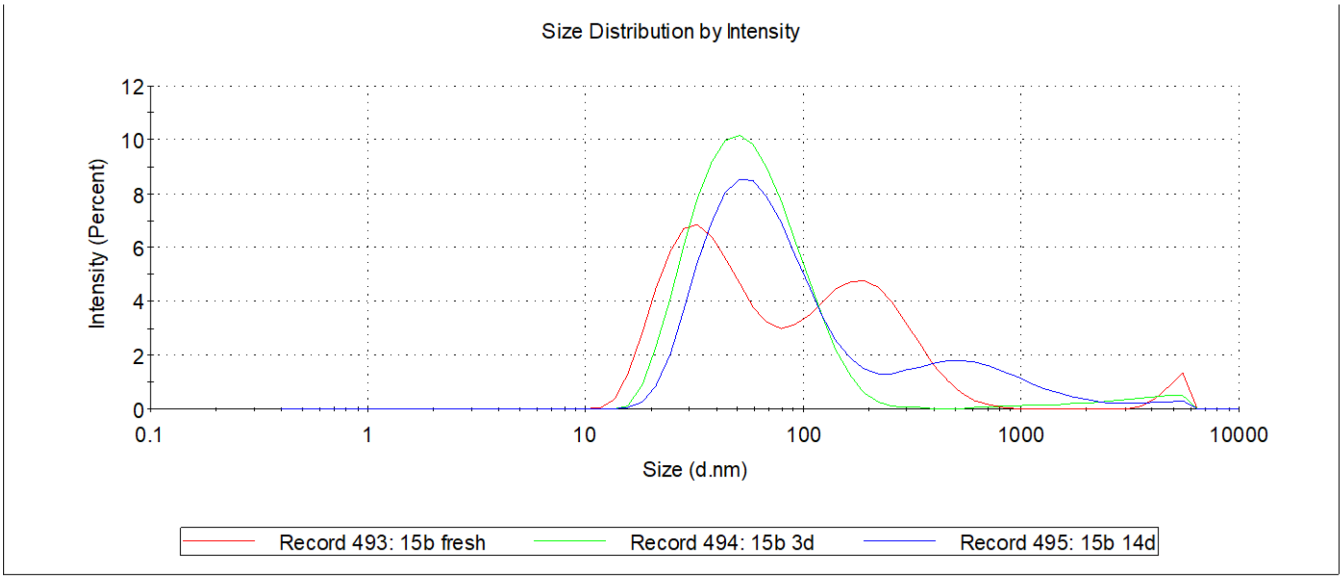


**Figure S42.** DLS size distribution of 1,1'-((3,5-bis(((2-hexyldecyl)oxy)carbonyl)-4-phenyl-1,4-dihydropyridine-2,6-diyl)bis(methylene))bis(4-methylpyridin-1-ium) dibromide (**15b**).

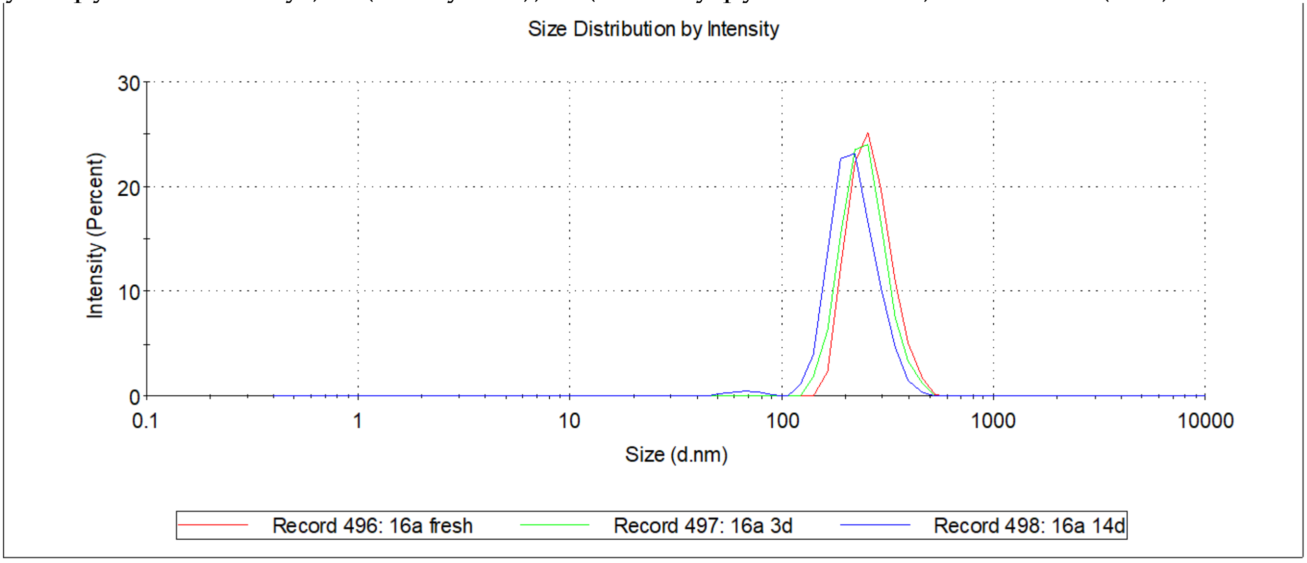


**Figure S43.** DLS size distribution of 1,1'-((3,5-bis(((2-ethylhexyl)oxy)carbonyl)-4-phenyl-1,4-dihydropyridine-2,6-diyl)bis(methylene))bis(4-(dimethylamino)pyridin-1-ium) dibromide (**16a**).

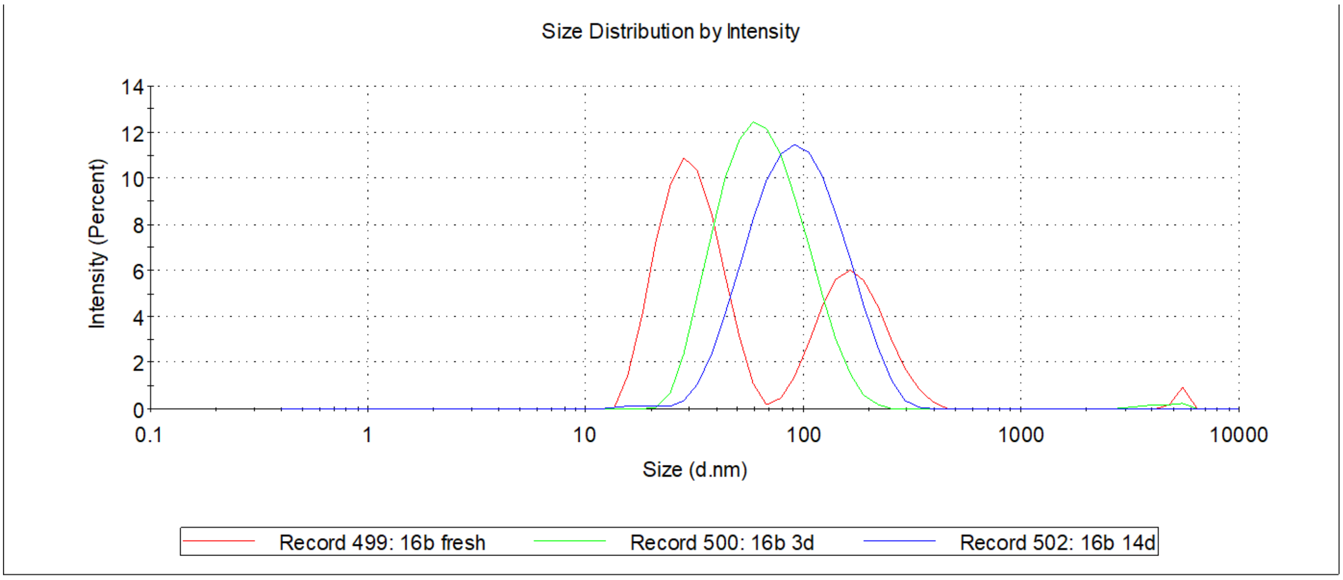


**Figure S44.** DLS size distribution of 1,1'-((3,5-bis(((2-hexyldecyl)oxy)carbonyl)-4-phenyl-1,4-dihydropyridine-2,6-diyl)bis(methylene))bis(4-(dimethylamino)pyridin-1-ium) dibromide (**16b**).

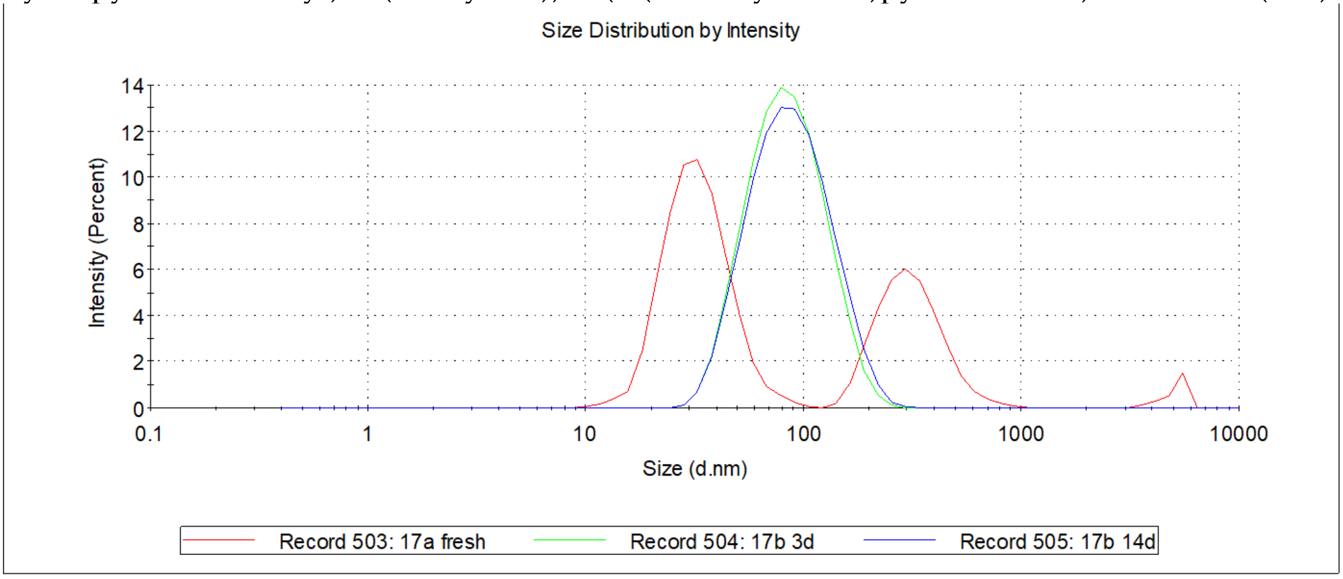


**Figure S45.** DLS size distribution of 1,1'-((3,5-bis(((2-hexyldecyl)oxy)carbonyl)-4-phenyl-1,4-dihydropyridine-2,6-diyl)bis(methylene))bis(4-phenylpyridin-1-ium) dibromide (**17b**).

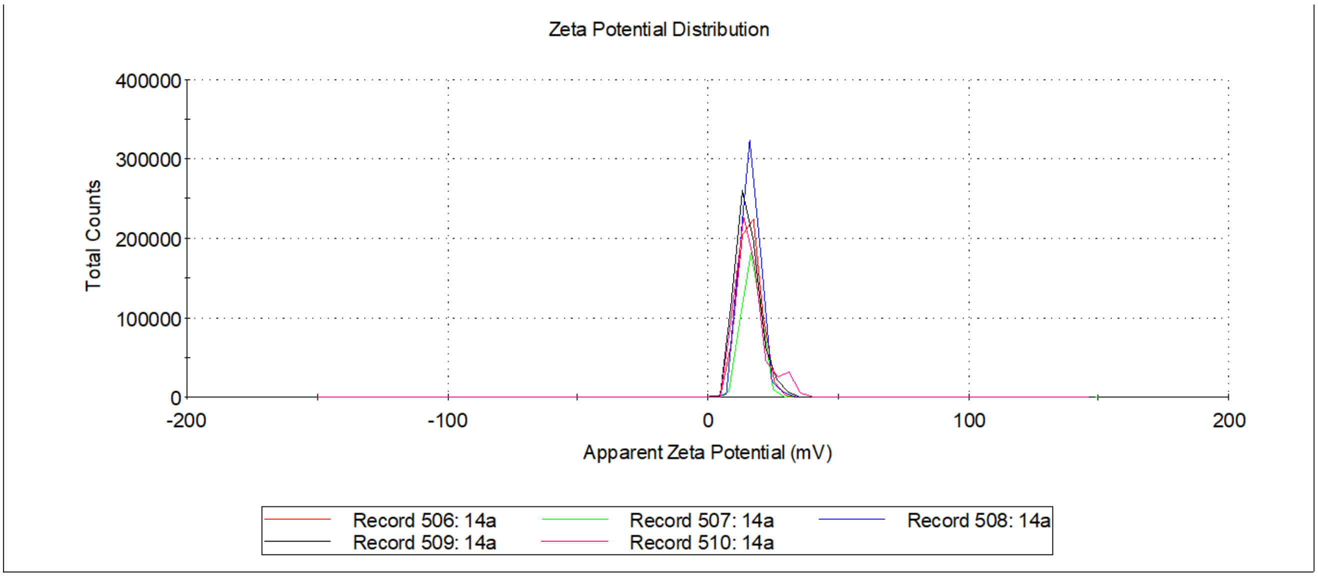


**Figure S46.** Zeta potential of 1,1'-((3,5-bis(((2-ethylhexyl)oxy)carbonyl)-4-phenyl-1,4-dihydropyridine-2,6-diyl)bis(methylene))bis(pyridin-1-ium) dibromide (**14a**).

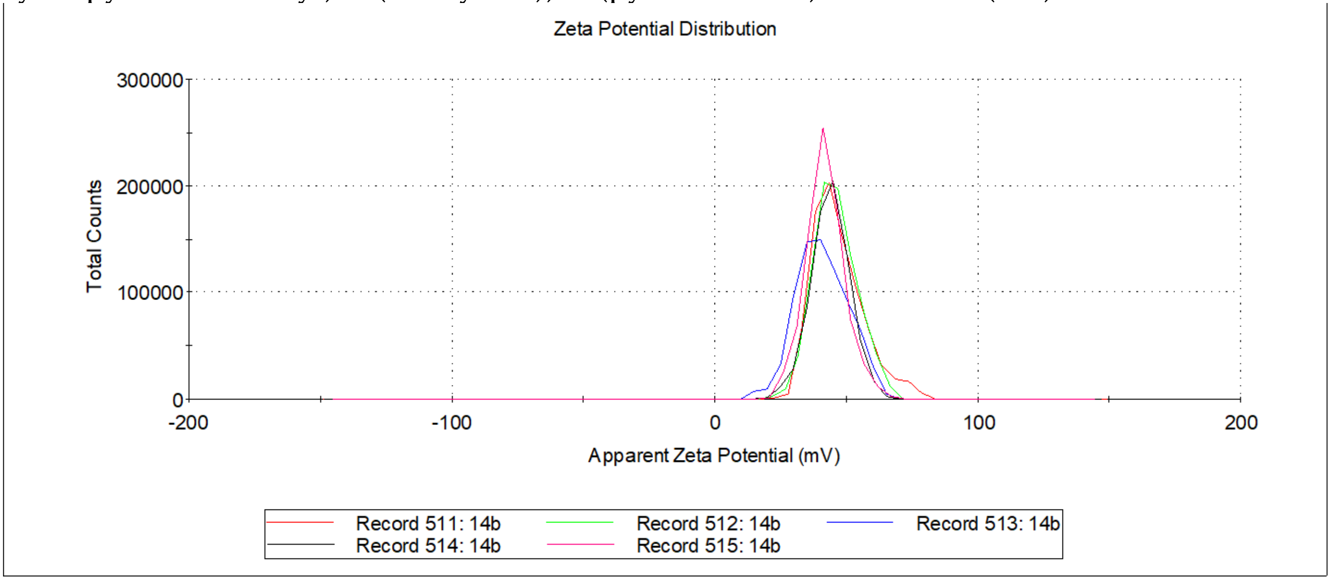


**Figure S47.** Zeta potential of 1,1'-((3,5-bis(((2-hexyldecyl)oxy)carbonyl)-4-phenyl-1,4-dihydropyridine-2,6-diyl)bis(methylene))bis(pyridin-1-ium) dibromide (**14b**).

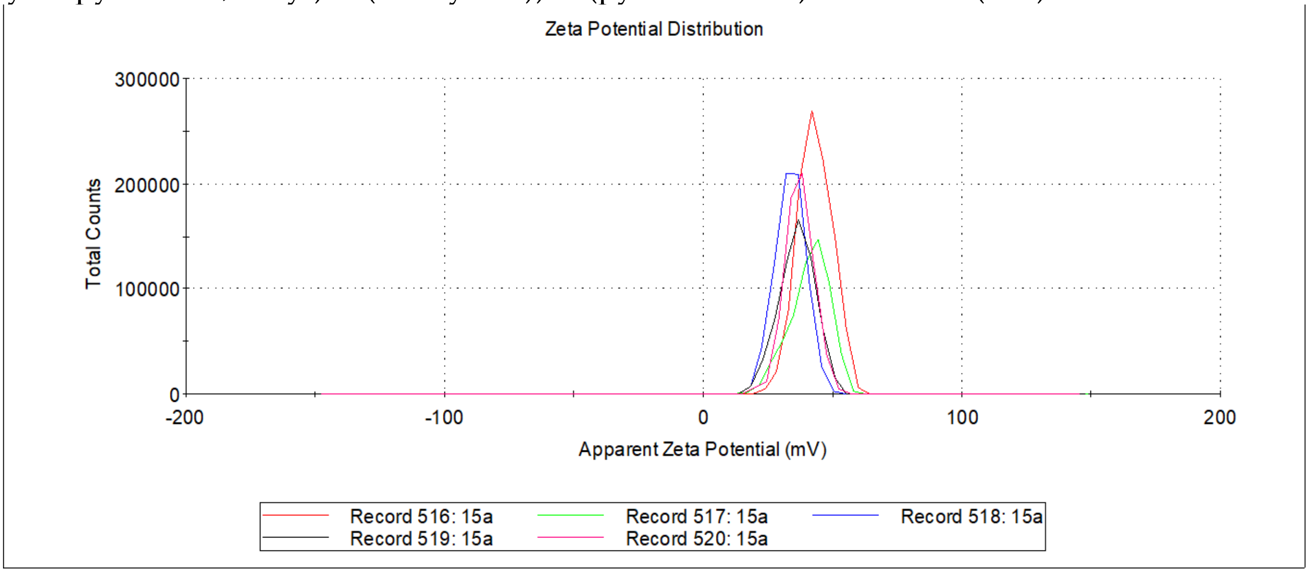


**Figure S48.** Zeta potential of 1,1'-((3,5-bis(((2-ethylhexyl)oxy)carbonyl)-4-phenyl-1,4-dihydropyridine-2,6-diyl)bis(methylene))bis(4-methylpyridin-1-ium) dibromide (**15a**).

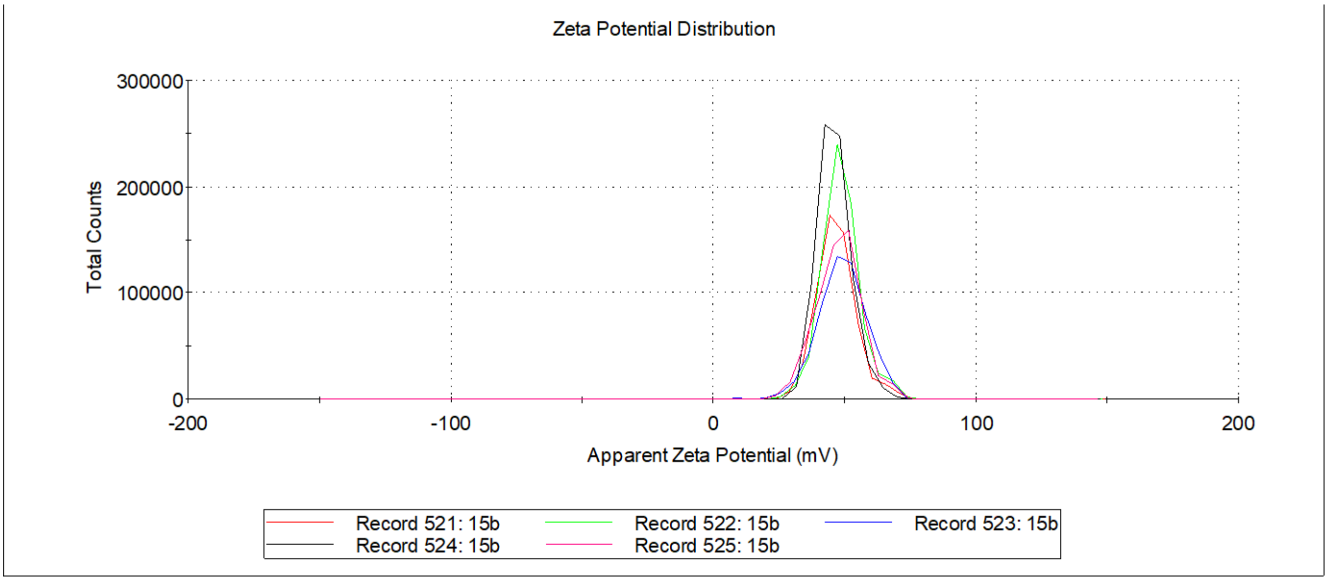


**Figure S49.** Zeta potential of 1,1'-((3,5-bis(((2-hexyldecyl)oxy)carbonyl)-4-phenyl-1,4-dihydro-pyridine-2,6-diyl)bis(methylene))bis(4-methylpyridin-1-ium) dibromide (**15b**).

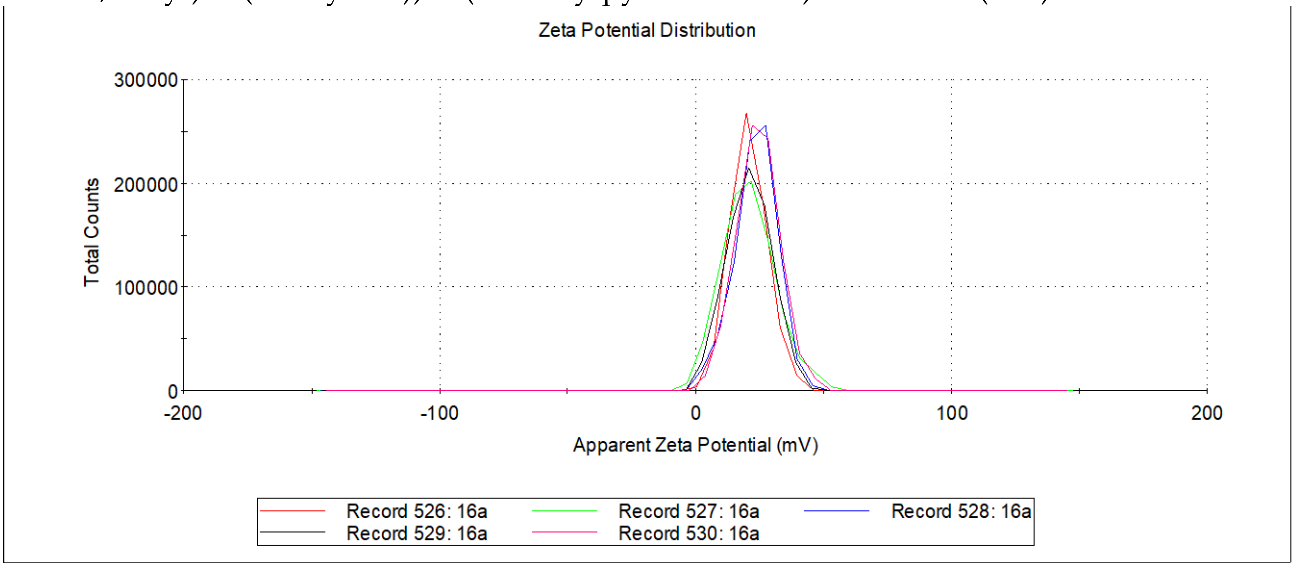


**Figure S50.** Zeta potential of 1,1'-((3,5-bis(((2-ethylhexyl)oxy)carbonyl)-4-phenyl-1,4-dihydro-pyridine-2,6-diyl)bis(methylene))bis(4-(dimethylamino)pyridin-1-ium) dibromide (**16a**).

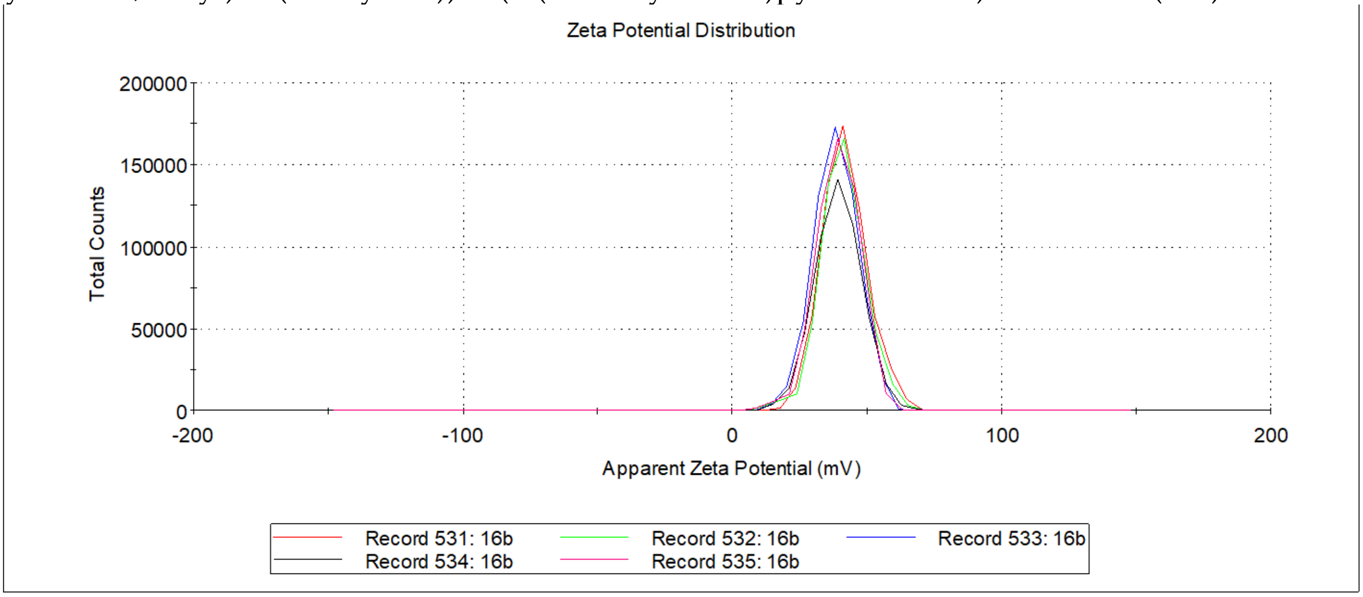


**Figure S51.** Zeta potential of 1,1'-((3,5-bis(((2-hexyldecyl)oxy)carbonyl)-4-phenyl-1,4-dihydro-pyridine-2,6-diyl)bis(methylene))bis(4-(dimethylamino)pyridin-1-ium) dibromide (**16b**).

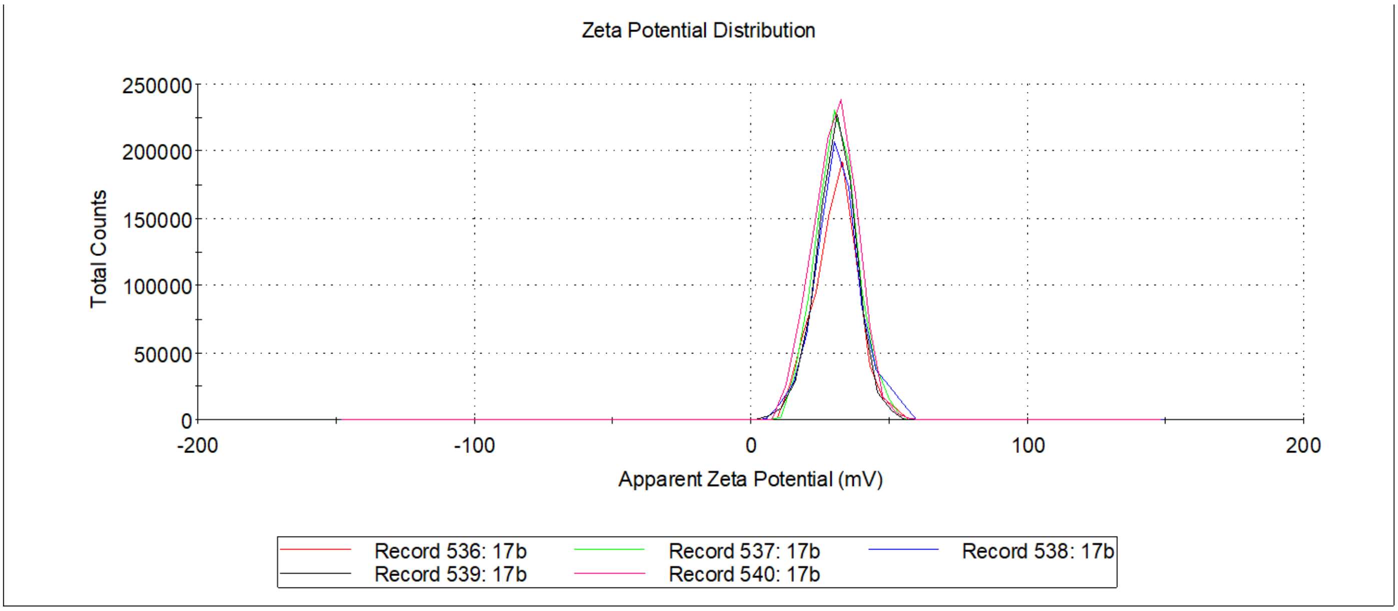


**Figure S52.** Zeta potential of 1,1'-((3,5-bis(((2-hexyldecyl)oxy)carbonyl)-4-phenyl-1,4-dihydropyridine-2,6-diyl)bis(methylene))bis(4-phenylpyridin-1-ium) dibromide (**17b**).

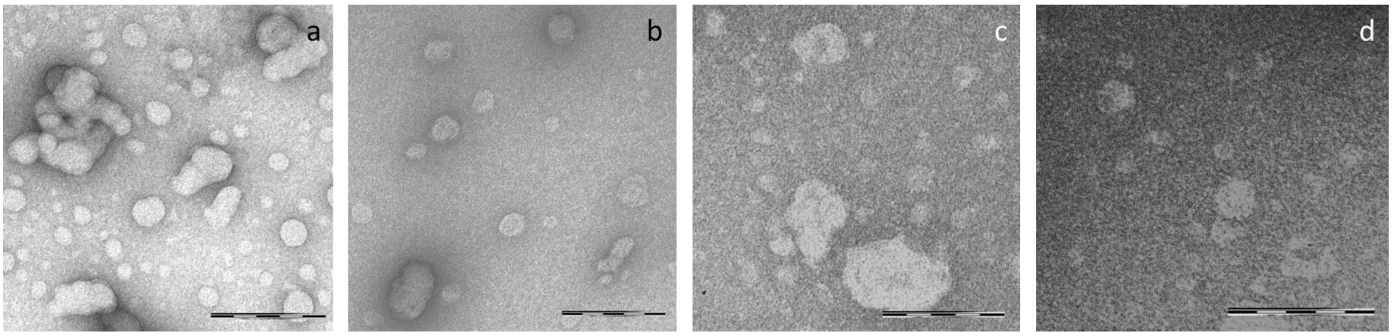


**Figure S53.** TEM images of formed nanostructures for the samples of **a)** amphiphile **14a**; **b)** amphiphile **14b**; **c)** amphiphile **15a**; **d)** amphiphile **15b** adsorbed to carbon-coated grids and negatively stained with freshly prepared 2% uranyl acetate aqueous solution. The stock samples were prepared by the ethanol injection method following sonication; concentrations of samples - 0.5 mM. Bars = 200 nm.
